# Precision genome editing and in-cell measurements of oxidative DNA damage repair enable functional and mechanistic characterization of cancer-associated *MUTYH* variants

**DOI:** 10.1093/nar/gkaf037

**Published:** 2025-03-28

**Authors:** Carlos A Vasquez, Nicola R B Osgood, Marcanthony U Zepeda, Dominika K Sandel, Quinn T Cowan, Malalage N Peiris, Daniel J Donoghue, Alexis C Komor

**Affiliations:** Department of Chemistry and Biochemistry, University of California San Diego, La Jolla, CA 92093, United States; Department of Chemistry and Biochemistry, University of California San Diego, La Jolla, CA 92093, United States; Department of Chemistry and Biochemistry, University of California San Diego, La Jolla, CA 92093, United States; Department of Chemistry and Biochemistry, University of California San Diego, La Jolla, CA 92093, United States; Department of Chemistry and Biochemistry, University of California San Diego, La Jolla, CA 92093, United States; Department of Chemistry and Biochemistry, University of California San Diego, La Jolla, CA 92093, United States; Department of Chemistry and Biochemistry, University of California San Diego, La Jolla, CA 92093, United States; Moores UCSD Cancer Center, University of California San Diego, La Jolla, San Diego, CA 92093, United States; Department of Chemistry and Biochemistry, University of California San Diego, La Jolla, CA 92093, United States; Moores UCSD Cancer Center, University of California San Diego, La Jolla, San Diego, CA 92093, United States; Sanford Stem Cell Institute, University of California San Diego, La Jolla, CA 92037, United States

## Abstract

Functional characterization of genetic variants has the potential to advance the field of precision medicine by enhancing the efficacy of current therapies and accelerating the development of new approaches to combat genetic diseases. MUTYH is a DNA repair enzyme that recognizes and repairs oxidatively damaged guanines [8-oxoguanine (8-oxoG)] mispaired with adenines (8-oxoG·A). While some mutations in the *MUTYH* gene are associated with colorectal cancer, most *MUTYH* variants identified in sequencing databases are classified as variants of uncertain significance. Convoluting clinical classification is the absence of data directly comparing homozygous versus heterozygous *MUTYH* mutations. In this study, we present the first effort to functionally characterize *MUTYH* variants using precision genome editing to generate heterozygous and homozygous isogenic cell lines. Using a MUTYH-specific lesion reporter in which we site-specifically incorporate an 8-oxoG·A lesion in a fluorescent protein gene, we measure endogenous MUTYH enzymatic activity and classify them as pathogenic or benign. Further, we modify this reporter to incorporate the MUTYH repair intermediate (8-oxoG across from an abasic site) and validate it with co-immunoprecipitation experiments to demonstrate its ability to characterize the mechanism by which MUTYH mutants are defective at DNA repair.

## Graphical Abstract

**Graphical Abstract:**
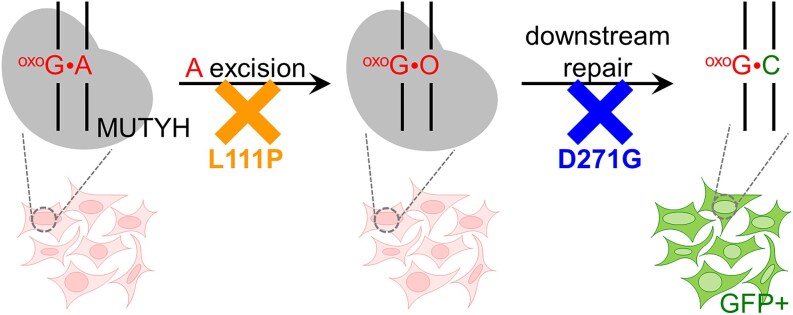

## Introduction

Progress in next-generation sequencing (NGS) technologies has streamlined the detection of human genetic variations [1], and the identification of clinically actionable genes and pathogenic mutations has transformed precision medicine. However, only a small fraction of identified human genetic variants have been assigned a clinical classification. The Genome Aggregation Database has documented 787 million genetic variants (96% of which are single-nucleotide variants, or SNVs) [2–4] but <0.5% possess clinical interpretations in the ClinVar database [5]. Furthermore, 99% of these identified variants are rare or unique to specific ethnic populations, making it particularly challenging to predict their functional impact through genome-wide association studies (GWAS) or computational methods [6–8]. In particular, mutations in genes encoding for DNA repair proteins are linked to such diseases as cancer, premature aging, immune deficiencies, and neurodegenerative disorders [9–11], highlighting the importance of functionally characterizing genetic variants and obtaining mechanistic insight into disease etiology and progression.

The human *MUTYH* gene (which encodes for a DNA repair protein) exemplifies the variant interpretation problem: of the 2517 total SNVs currently listed in ClinVar, 58.2% are listed as a variants of uncertain significance (VUS) or have conflicting reports. However, loss-of-function mutations in this gene are associated with multiple cancer types, the most common of which being MUTYH-associated polyposis (MAP), an increased risk of developing colorectal cancer [12]. Furthermore, while most clinical data suggest that MAP is caused by autosomal recessive (homozygous) point mutations [13], there are still conflicting reports concerning carrier (heterozygous) genotypes [14–19]. Overall, the clinical classification of VUS in the *MUTYH* gene using computational methods has been a major challenge, underscoring the importance of evaluating the functional consequences of *MUTYH* variants using cellular models.

**Figure 1.**
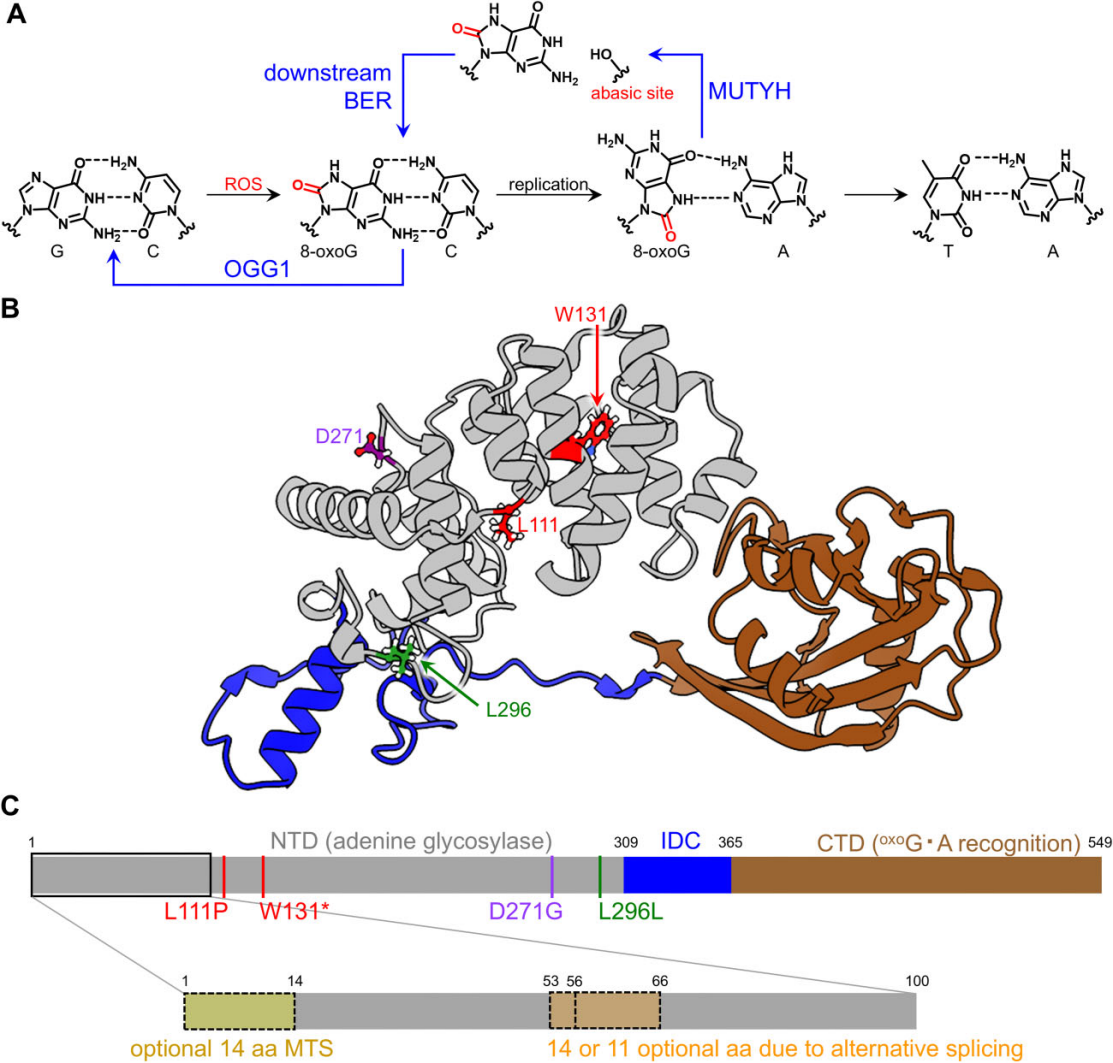
Overview of MUTYH structure and function. (**A**) ROS readily oxidize guanine (G) bases in DNA into 8-oxoG. In mammals, the glycosylases OGG1 and MUTYH initiate the BER process of recognizing, excising, and replacing 8-oxoG lesions. If 8-oxoG is still base-paired with cytosine (8-oxoG•C), OGG1 will recognize the lesion and initiate BER to convert it back to a canonical G•C base-pair. However, if the 8-oxoG•C is not repaired before DNA replication, 8-oxoG will preferentially base-pair with adenine (A) through Hoogsteen interactions to form an 8-oxoG•A mismatch. MUTYH recognizes 8-oxoG•A lesions and excises A to create an apurinic/apyrimidinic site (abasic site, AP site; [O]) across from 8-oxoG (8-oxoG•[O]), which is then further processed by downstream BER proteins back into 8-oxoG•C. If, however, MUTYH does not recognize or cannot repair 8-oxoG•A prior to an additional round of DNA replication, thymine (T) will be incorporated opposite the A, resulting in a permanent G•C to T•A mutation. (**B**) Structural model of MUTYH (AlphaFold model AF-Q9UIF7-F1), colored by domain, and with amino acid residues mutated in this study labeled. Amino acids are colored according to ClinVar classification (L111P and W131* are pathogenic or likely pathogenic, D271G is a variant of uncertain significance, and L296L is benign or likely benign). There are a Zn center and FeS cluster missing from the AlphaFold model, and the first 86 and last 51 amino acids are omitted for clarity, as the model confidence of these regions is low. (**C**) Domain map of isoform 5 of MUTYH is shown according to (B) and with mutations studied in this work indicated. Inset is showing a zoom-in of the first 100 amino acids and the optional regions due to alternative splicing; CTD, C-terminal domain; MTS, mitochondrial targeting signal; NTD, N-terminal domain.

*MUTYH* encodes for the MUTYH protein, an enzyme that repairs damage caused by reactive oxygen species (ROS). The most readily oxidized DNA base is guanine (G), which is converted to 8-oxoguanine (8-oxoG, Fig. 1A) [20, 21]. In mammals, the glycosylases OGG1 and MUTYH initiate the base excision repair (BER) process of recognizing, excising, and replacing 8-oxoG lesions (Fig. 1A) [19, 22–27]. If 8-oxoG is still base-paired with cytosine (8-oxoG•C), OGG1 will recognize the lesion and initiate its repair (Fig. 1A). However, if the 8-oxoG•C is not repaired before DNA replication, 8-oxoG will preferentially base-pair with adenine (A) to form an 8-oxoG•A mismatch (Fig. 1A) [21, 28, 29]. MUTYH recognizes 8-oxoG•A lesions and excises A to create an abasic site (8-oxoG•[O] lesion intermediate), which is then further processed by downstream BER proteins into 8-oxoG•C (Fig. 1A). If, however, 8-oxoG•A is not repaired prior to an additional round of DNA replication, thymine (T) will be incorporated opposite the A, resulting in a permanent G•C to T•A mutation.

Notably, the DNA repair process for 8-oxoG is a tightly coordinated process involving multiple proteins that work together to detect damage and regulate repair before mutations can be generated [30]. MUTYH both performs enzymatic reactions (adenine excision) and interacts with and recruits other enzymes to the site of damage to complete the 8-oxoG•A repair process [31–36]. This highlights the importance of studying MUTYH in its native context of the cell and under endogenous expression levels. APE1 (apurinic/apyrimidinic endodeoxyribonuclease 1) is one such interaction partner of MUTYH, and studies have suggested that it interacts with MUTYH via amino acids 309 to 331 of the interdomain connector (IDC, Fig. 1B and C) [31, 33]. Immediately after excision of A from the 8-oxoG•A lesion by MUTYH to produce 8-oxoG•[O], APE1 incises the phosphodiester backbone at the abasic site. Several additional BER proteins (such as polymerases, endonucleases, ligases, and scaffolding factors) then process and fill in this subsequent intermediate to yield 8-oxoG•C.

While thousands of MUTYH variants have been observed in humans, most are understudied from a mechanistic standpoint. This problem is primarily due to technical challenges in studying DNA repair proteins in a physiologically relevant context, especially in high-throughput. When comparing patient-derived cell lines that harbor different *MUTYH* variants to each other, differences in background genetic variation can impact cellular function and thus convolute the determination of whether variants of interest are pathogenic or merely co-inherited benign mutations [37]. Previous work to functionally characterize *MUTYH* variants in live cells with identical genetic backgrounds has involved the introduction of an exogenous expression construct of *MUTYH* variants (usually using *MUTYH* cDNA [complementary DNA]) into cells that may (or may not) have the endogenous *MUTYH* gene knocked out [27,38–48]. Notably, the use of exogenously supplied MUTYH ignores its natural expression levels, which can alter the biology of the protein and how it interacts with its fellow BER proteins. Further, due to multiple transcription initiation sites and alternative splicing, more than nine different isoforms of MUTYH exist in human cells, which are not faithfully recapitulated with cDNA overexpression experiments [29]. Finally, these types of experiments cannot properly model heterozygous genotypes. Because of the intricacies of the BER pathway, it is of the utmost importance to study MUTYH in its native environment within a living cell and without altering its native expression level or those of its interacting partners, as this can alter the stoichiometry with which they interact. Introducing mutations into the endogenous MUTYH gene of living cells would provide the most accurate model to study such variants mechanistically, enabling clinical classification and elucidating disease mechanisms. However, the introduction of SNVs into the genome of mammalian cells using traditional genome editing methods (such as CRISPR–Cas9) suffers from low efficiencies and high byproduct formation, making the generation of cellular models of variants time- and resource-intensive [49–52].

Here, we report using base editing to overcome many of the limitations of traditional genome editing and generate both homozygous and heterozygous isogenic cell lines of four *MUTYH* SNVs. To date, these are the first reports of successful generation of isogenic cell lines containing *MUTYH* SNVs. We adapt a fluorescent reporter for 8-oxoG•A repair activity for our system and directly measure the MUTYH repair efficiencies of each mutant compared to wild-type in our cell lines. We are able to define thresholds in the signal from this assay using known pathogenic (L111P and W131*) and benign (L296L) variants (according to the guidelines established by the Clinical Genome [ClinGen] Resource) [53–56], which enable the putative classification of a VUS (D271G) as pathogenic. We further find only homozygous mutations to have reduced 8-oxoG•A repair capacity. We expand on this fluorescent reporter to measure 8-oxoG•[O] repair activity in live cells, which reports on the ability of MUTYH to coordinate downstream BER of its substrate, and find the D271G mutant to be defective at coordinating repair of this intermediate. We then use co-immunoprecipitation (co-IP) to find that the D271G mutation disrupts the MUTYH–APE1 interaction. This work describes a general strategy for studying genetic variants in DNA repair protein genes using a combination of precision genome editing, chemical biology, and biochemical techniques. This strategy enables the identification of variants with reduced DNA repair capacity (and thus eventual clinical classification) and provides mechanistic information on how each defective mutant cannot perform its function.

## Materials and methods

### General methods and cloning

All primers in this study were ordered through Integrated DNA Technologies (IDT). All polymerase chain reaction (PCR) reactions were performed with Phusion DNA Green High-Fidelity Polymerase (Thermo Fisher Scientific, #F534L) or Phusion U (Thermo Fisher, #F556L) where appropriate. Guide RNA (gRNA) plasmids were cloned by site-directed mutagenesis using a 5′ tail in the forward primer to replace the 20 nt spacer region (Basic Protocol 1 [57]; *Streptococcus pyogenes* Cas9 gRNA vector Addgene plasmid #47511). Primer sequences can be found in Supplementary Table S1. Base editor (BE) plasmids were generated by taking either AncBE4max-P2A-GFP (Addgene plasmid #112100) or ABE7.10max-P2A-GFP (Addgene plasmid #112101) and replacing the C-terminal 300 amino acids of Cas9n with that from SpCas9-NG (Addgene plasmid #117919) using USER (Uracil-Specific Excision Reagent) cloning [58] to produce cytosine base editor (CBE) and adenine base editor (ABE) variants that recognize a relaxed PAM (protospacer adjacent motif) of NG (N = any DNA base; G = Guanine).

The intact mCherry-P2A-EGFP reporter plasmids were generated with USER cloning following New England Biolabs (NEB) protocols, by replacing the ABE gene in the ABE-P2A-EGFP plasmid (Addgene plasmid #112101) with the mCherry gene from the pBAD-mCherry plasmid (Addgene plasmid #54630). The sequence of the mCherry-P2A-EGFP open reading frame used in this work can be found in the “Supplementary Sequences” section. All variations (i.e., point mutations) on this plasmid were cloned using site-directed mutagenesis [59]. The pCAV035 parental DNA Repair reporter plasmid (Addgene plasmid #219807) with a custom Golden Gate site was generated via USER cloning from the pCAV033 plasmid (mCherry_P2A_EGFP construct) to insert a unique DNA sequence containing two BsaI recognition sites at codon 34 of enhanced green fluorescent protein (EGFP). To generate reporter constructs with custom base pairs at codon 34, pCAV035 was digested with BsaI-HFv2 (NEB, #R3733S) according to the manufacturer’s protocol and purified using a gel extraction kit (Qiagen, #28704). Next, preannealed oligos containing synthetic DNA lesions (sequences of which can be found in Supplementary Table S3) and complementary sticky ends matching the digested overhangs were annealed into the digested site using T4 DNA ligase (NEB, #M0202) overnight at 12°C. The ligation product was then purified using columns from the QIAquick PCR purification kit (Qiagen, #28106). The abasic site-containing oligo (for the for the 8-oxoG•[O] reporter) was produced by digestion of the oligo listed in Supplementary Table S3 with uracil–DNA glycosylase (UDG; NEB # M0280S) according to the manufacturer’s protocol prior to ligation into the digested pCAV035 plasmid. All plasmids used in our transfections were endotoxin-free and prepared with the ZymoPURE II Midiprep Kit (Zymo Research, #D4201). Sequences of the pCAV033 and pCAV035 constructs are available in Supplementary Data. The 8-oxoG-containing oligo (listed in Supplementary Table S3) was both obtained and quality controlled by IDT using capillary electrophoresis and electrospray ionization mass spectrometry.

### Cell culture

HEK293T cells (ATCC CRL-3216) were cultured in high glucose Dulbecco’s modified Eagle’s medium (DMEM) supplemented with GlutaMAX (Thermo Fisher Scientific, #10566–016) and 10 % (v/v) fetal bovine serum (Thermo Fisher Scientific, #10437–028) at 37°C with 5% CO_2_. Cells were passaged every 3 days using TrypLE (Thermo Fisher Scientific, #12 605 028).

### Transfections for base editing experiments

HEK293T cells were seeded at 50 000 cells/well in 250 μl of media in a 48-well plate and transfected after 16 h, when they were at ∼70% confluency [57]. Mixtures of plasmids encoding gRNA and appropriate BE were created in a total volume of 12.5 μl with Opti-MEM reduced-serum medium (Thermo Fisher Scientific, #31985–070) using 200 ng gRNA plasmid and 800 ng of CBE or ABE plasmid. These DNA mixtures were then combined with a mixture comprised of 1.5 μl of Lipofectamine 2000 regent (Thermo Fisher Scientific, #11668030) and 11 μl of Opti-MEM, incubated at room temperature for 15 min and added to the plated cells. To measure bulk base editing efficiencies, cells were incubated for 72 h and washed with 150 μl of phosphate-buffered saline (PBS, Gibco #10010–023), and genomic DNA (gDNA) was extracted by adding 100 μl of freshly prepared buffer containing 10 mM Tris–HCl (pH 7.0), 0.05% SDS, and 25 μg/ml of Proteinase K (NEB, #P8107S). Digestion was done at 37°C for 1 h, followed by an 80°C heat treatment for 30 min. The gDNA was then used as a template for Sanger sequencing or NGS.

### Sanger sequencing of the MUTYH locus

To obtain Sanger sequencing of each *MUTYH* variant, a 50 μl of PCR reaction was run, using the primers specified in Supplementary Table S2, to amplify the genomic loci of interest using the Phusion high-fidelity DNA polymerase protocol (NEB, #M0530) along with the thermocycler parameters previously described (Basic Protocol 3) [57]. Once the presence of the predicted PCR product had been confirmed via gel electrophoresis, the samples were sent to the Genewiz (from Azenta Life Sciences) using their unpurified PCR-product Sanger sequencing service. In instances in which Sanger sequencing results from the unpurified PCR-product service failed, the purified PCR-product service was used. For this service, PCR products were purified using the QIAquick PCR purification kit (Qiagen, #28106), and then the appropriate amount of PCR product (according to the Genewiz sample submission guidelines) was pre-mixed with 5 μl of either the forward or reverse primer (5 μM).

### Fluorescence-activated cell sorting

For the generation of isogenic cell lines, transfections were repeated as described above. After 72 h, cells were washed with 150 μl of PBS and detached with 30 μl of Accumax (Innovative Cell Technologies, #AM-105) at room temperature. After 1 min, cells were resuspended with 170 μl of cold PBS, passed through a fluorescence-activated cell sorting (FACS) tube containing a 35 μm cell strainer (Falcon, #352235) and kept on ice. EGFP-positive cells were sorted into either individual wells of a 96-well plate containing 100 μl of DMEM with 50% FBS and 1% Pen/Strep or sorted in bulk (at least 5000 cells) for dilution plating as previously described [57]. FACS was performed on a FACSAria II. Example gating schemes used for these experiments are shown in Supplementary Fig. S1. Cells were expanded for 1–2 weeks and then genotyped with Sanger sequencing as described above or NGS as described below.

### Next-generation sequencing of isogenic cell lines

After isogenic cell line generation, on-target and potential off-target (see Supplementary Table S4 for sequences of off-target loci) genome editing loci of isogenic cell lines were sequenced via targeted amplicon NGS [57, 60]. Briefly, gDNA was extracted from ∼70 000 cells after washing the cells with 150 μl of PBS and lysing in 100 μl of digestion buffer as described above in “Transfections for base editing experiments” section. Genomic loci of interest were amplified via two rounds of PCR. Round 1 PCRs were completed using a 25 μl of PCR reaction with Phusion polymerase (Thermo Fisher Scientific, #F534L) comprised of 1 μl of gDNA, GC buffer, 3% DMSO (dimethyl sulfoxide) and 25% of the recommended primer amount (to reduce the amount of primer dimers; NGS primers are listed in Supplementary Tables S2 and S5; these locus-specific primers were designed to contain an adapter sequence, allowing for sample barcoding with a second round of PCR) and amplified for 24–27 cycles (minimal amount to avoid PCR bias) using an annealing temperature of 62°C and an extension time of 25 s. After confirmation of the accurately sized product on a 2% agarose gel, Round 2 PCR was performed to barcode samples in 10  μl of total reaction volume, comprised of 0.10 μl of Phusion polymerase, 5.95 μl of water, 2 μl of 5× HF buffer, 0.2 μl of dNTPs, 1.25 μl of each primer (0.0625  μM), and 0.5  μl of Round 1 PCR product, and amplified for 8–12 cycles using an annealing temperature of 65°C and an extension time of 25 s. Round 2 PCR products were pooled together based on amplicon size and purified from a 2% agarose gel using a gel extraction kit (Qiagen, #28704). The resulting purified libraries were then quantified with the Qubit dsDNA High Sensitivity Kit (Thermo Fisher Scientific, #Q32854). Samples were then diluted to 1.4 pM following Illumina’s sample preparation guidelines. The final library was mixed with 1.4 pM PhiX sequencing control (10% v/v) and then sequenced on an Illumina MiniSeq via paired end sequencing (2 × 151 paired end reads).

### Transfections for flow cytometry

HEK293T cells were seeded at 100 000 cells/well in 250 μl of media in a 48-well plate and transfected 16 h after plating, when they were at ∼70% confluency. Five hundred nanograms of mCherry-P2A-EGFP reporter plasmid (intact plasmids or with custom inserts) was diluted to a total volume of 12.5 μl with Opti-MEM reduced-serum medium. The DNA mixture was then combined with a solution of 1.5 μl of Lipofectamine 2000 regent in 11 μl of Opti-MEM and added to the plated cells. Cells were then incubated for 24 h before harvesting for the flow cytometry.

### Flow cytometry analysis of DNA repair with EGFP reporter vectors

For all DNA reporter fluorescence measurements, the medium was removed from each well, and each well was washed with 150 μl of PBS. To detach cells, 30 μl of Accumax was added to each well. Cells were counted and diluted to a concentration of 1 × 10^6^ cells/ml in PBS, then strained into 5 ml tubes through a cell strainer cap (Corning, #352235) and kept on ice. Flow cytometry data were collected using a Bio-Rad S3e cell sorter equipped with 488, 561, and 640 nm lasers, and analyzed using FlowJo v10.8.1 Software (BD Life Sciences). Scatter gates were applied to remove nonviable cells and doublets. For reporter experiments, gates were applied based on cells transfected with mCherry only or EGFP only plasmids. mCherry fluorescence was detected using FL3 (602–627 nm) and a PMT voltage of 360. EGFP fluorescence was detected using FL1 (510–540 nm). A total of 10 000 cells (after scatter gating) were collected for each sample. Example gating schemes used for these experiments are shown in Supplementary Fig. S2.

### Preparation of cell extracts and western blotting

Roughly 20 × 10^6^ cells were harvested from a 150 mm dish by first removing the medium and washing with 2 ml of cold PBS. To detach cells, 1 ml of RIPA (radioimmunoprecipitation assay) buffer (1% Triton X-100, 0.5% DOC, 0.1% SDS, and 50 mM of Tris, pH 7.4) plus Halt Protease and Phosphatase Inhibitor Cocktail (Thermo Fisher Scientific, #78 440) was used. The resulting suspension was then transferred into a precooled microcentrifuge tube. The cells were then maintained at constant agitation for 30 min at 4°C. The cell suspension was then centrifuged for 10 min at 16 000 × r.c.f at 4°C. After centrifugation, the tubes were placed on ice and the supernatant was aspirated and placed in a fresh tube kept on ice. Protein concentrations were determined using the Pierce BCA Protein Assay Kit (Thermo Fisher Scientific, #23225). Equal amounts of total protein from clarified cell lysate solutions (40 μg of total protein) were mixed with NuPAGE LDS Sample Buffer (Invitrogen #NP0007) plus 10 mM dithiothreitol (DTT, Thermo Fisher Scientific, #R0861) to a total volume of 80 μl and heated for 10 min at 95°C, then 40 μl of each sample was loaded and electrophoresed on a 7.5% Criterion™ TGX™ Precast Midi Protein Gel (Bio-Rad, #5671024). The protein was then transferred to a 0.45-μm PVDF (polyvinylidene fluoride) transfer membrane (Thermo Fisher Scientific, #88 585) using the Mini Trans-Blot^®^ Cell (Bio-Rad, #1703930; 50 V, 60 min). The membrane was then incubated with Revert Total Protein Stain (LI-COR #926–11 010) and washed according to the manufacturer’s instructions. The membrane was then blocked with 5 ml of 5% milk in Tris-buffered saline with 0.1% Tween 20 (TBST; 20 mM Tris, 150 mM NaCl, and 0.1% Tween 20 from Thermo Fisher Scientific, #85114) for 1 h and then incubated overnight at 4°C with MUTYH (C-6) primary antibody (Santa Cruz Biotechnology #sc-374571) diluted 1:500. The membrane was washed three times with 5 ml of TBST for each wash, then incubated with HRP-anti-mouse IgG (Cell Signaling #7076) diluted 1:2000 in 10 ml of TBST for 1 h at room temperature. The membrane was then washed again three times with 5 ml of TBST, then soaked in 200 μl of chemiluminescent substrate (Bio-Rad #1705061) for 5 min and imaged with a Syngene G:Box Chemi XX6 imager.

### Co-immunoprecipitation experiments

The homozygous cell lines and wild-type HEK293T cells were seeded into six-well plates at a density of 5 × 10^5^ per well in 2 ml of media and incubated overnight. Prior to harvesting, the cells were treated with 0.5 mM hydrogen peroxide (H_2_O_2_) for 1 h. The cells were then harvested by first removing the medium and washing with 2 ml of cold PBS. To detach cells, 200 μl of TrypLE (Thermo Fisher Scientific, #12605028) was used. The cells were then transferred to a precooled microcentrifuge tube and then centrifuged at 500 × r.c.f. for 5 min at 4°C. The cells were then washed by suspending the cell pellet with 1 ml of cold PBS. The cells were then transferred to another precooled microcentrifuge tube and then centrifuged again at 500 × r.c.f. for 3 min at 4°C. The supernatant was then carefully removed and discarded. NE-PER Nuclear and Cytoplasmic Extraction Reagent (Thermo Fisher Scientific #78833) plus Halt Protease and Phosphatase Inhibitor Cocktail was used to lyse the cell pellets. Briefly, 500 μl of cytoplasmic extraction reagent I (CER I) was added to each tube and vortexed vigorously for 15 s. After a 10-min incubation on ice, 27.5 μl of cytoplasmic extraction reagent II (CER II) was added, vortexed for 5 s, and then incubated on ice for 1 min. The tube was then vortexed again for 5 s and centrifuged for 5 min at 16 000 × *g*. The supernatant (cytoplasmic extract) was then immediately transferred to a clean pre-chilled tube and stored at −80°C. The pellet faction, which contained the nuclei, was then resuspended with 250 μl of ice-cold nuclear extraction reagent (NER) and vortexed vigorously for 15 s. The tube was then placed on ice and vortexed for 15 s in 10 min increments for a total of 40 min. The tube was then centrifuged at 16 000 × *g* at 4°C for 10 min. The supernatant (containing the nuclear extract) was then transferred to a clean prechilled tube and placed on ice. Meanwhile, 50  μl of Dynabeads™ Protein G (Thermo Fisher Scientific #10004D) was washed in a microcentrifuge tube twice with 200 μl of lysis buffer (4000 × *g* for 1  min at 4°C). Ten micrograms of either MUTYH (C-6) (Santa Cruz Biotechnology, sc-374571) or Normal Mouse IgG**_3_** (Cell Signaling, 75952) antibody diluted in 200 μl of PBS (Gibco #10010–023) mixed with 0.1% Tween 20 (Thermo Fisher Scientific, #85114; to make PBST) was then added to beads and incubated at 10 min at room temperature. The supernatant was removed, and the beads were gently washed with 200 μl of PBST. Approximately 250 μl of the clarified nuclear extracted cell lysate and 750 μl of PBST were then added to the prewashed beads and incubated for 1  h on a rotation wheel at 4°C. After incubation, the supernatant was removed using a magnet, and the beads were gently washed three times with 500 μl of PBST after each wash. After the last wash step, 50  μl of 2× Laemmli sample buffer (Bio-Rad, #1610737) was added to the beads, and the bound complexes were eluted by boiling for 5  min at 95°C. The boiled samples were then loaded and electrophoresed on a 7.5% Criterion™ TGX™ Precast Midi Protein Gel (Bio-Rad #5671024). The protein was then transferred to a 0.45-μm PVDF transfer membrane (Thermo Fisher Scientific, #88585) using the Mini Trans-Blot^®^ Cell (Bio-Rad, #1703930; 50 V, 60 min). The membrane was then washed with ultrapure water for 2 min with shaking and then treated with SuperSignal Western Blot Enhancer (Thermo Fisher Scientific #46640). Briefly, the membrane was immersed with 10 ml of Antigen Pretreatment Solution and incubated at room temperature for 10 min with shaking. The solution was then discarded, and the membrane was then rinsed five times with 5 ml of ultrapure water. The membrane was then blocked with 5 ml of 5% nonfat dried milk in Tris-buffered saline with 0.1% Tween 20 (TBST; 20 mM Tris, 150 mM NaCl, and 0.1% Tween 20 from Thermo Fisher Scientific, #85114) for 1 h, and then incubated overnight at 4°C with the following primary antibodies diluted in 10 ml of TBST: MUTYH (C-6) (Santa Cruz Biotechnology #sc-374571, diluted 1:2500), APE1 (Thermo Fisher Scientific #PA5-29157, diluted 1:2500), and Normal Mouse IgG**_3_** (Cell Signaling #75952, diluted 1:3000). The membrane was washed three times with 5 ml of TBST for each wash, then incubated with VeriBlot for IP Detection Reagent (Abcam, #ab131366, diluted 1:10 000) in 10 ml of TBST for 1  h at room temperature. The membrane was then washed again three times with 5 ml of TBST and soaked in 200 μl of chemiluminescent SuperSignal West Atto Ultimate Sensitivity Substrate (Thermo Fisher Scientific, #A38554) and imaged with a Syngene G:Box Chemi XX6 imager.

### Data analysis and statistics

NGS data were collected, demultiplexed, and trimmed with Illumina Local Run Manager Generate FASTQ analysis module (v2.0) and MiniSeq control software (v2.2.1). The FASTQ files were analyzed with CRISPResso2 (version 2.0.20b) on batch mode (parameters: –base_edit -wc -10 -w 10 -q 30) to assess genomic base editing efficiencies [61]. All editing efficiencies values were reported as nucleotide percentages around the gRNA (from CRISPResso2). Sanger sequencing was analyzed by aligning the .ab1 trace file to a reference amplicon on Benchling (RRID:SCR_013955). To analyze data obtained from flow cytometers, the .fcs files were analyzed and quadrant plots were made using the FlowJo™ Software Version 10.8.1. by Becton, Dickinson and Company; 2021, 2022. Unpaired, one-tailed, parametric *t*-tests were performed when comparing unedited cells with homozygous *MUTYH* mutations using GraphPad Prism Version 10.2.1, GraphPad Software, Boston, Massachusetts USA (www.graphpad.com). Plots were also made using GraphPad Prism.

## Results

### Engineering isogenic cell lines with MUTYH SNVs

To study *MUTYH* variants in their native context and with the BER pathway intact, we chose to generate isogenic cell lines with endogenously mutated *MUTYH* loci. The generation of heterozygous and homozygous isogenic cell lines requires high genome editing efficiency and precision, and we expected this could be more easily achieved using base editing (Supplementary Fig. S3) rather than traditional, double-strand break (DSB)-mediated methods. Base editing is a precision genome editing strategy that uses the programmability of CRISPR systems but employs deaminase enzymes to chemically modify targeted nucleobases rather than install DSBs (Supplementary Fig. S3A). Two main classes of BEs currently exist: CBEs, which install C•G to T•A SNVs via cytosine deamination (Supplementary Fig. S3B), and ABEs, which install A•T to G•C SNVs via adenosine deamination (Supplementary Fig. S3C). With both classes of editors, the BE is programmed to edit a base of interest by a guide RNA (gRNA), which matches the DNA locus sequence of interest and enables Cas9 binding via base-pairing rules (Supplementary Fig. S3A). We initially designed BE:gRNA combinations to install 18 clinically relevant *MUTYH* SNVs (Supplementary Fig. S4), guided primarily by three considerations:

1. We sought to include both pathogenic and benign variants to serve as positive and negative controls, and VUS so our studies could provide new clinical insights. Clinical classifications were obtained from the ClinVar database.
2. We focused on mutations that correspond to amino acids in the N-terminal domain of the MUTYH protein (amino acid residues 1–308 of isoform 5), as this domain of human MUTYH has been structurally determined [62]. Therefore, we could use structural information to guide experimental interpretations of such variants.
3. Technical considerations were taken into account, such as predicted base editing efficiencies and potential bystander mutations (bystander editing occurs when the deaminase enzyme inadvertently edits additional Cs or As near the target base).

We chose to use HEK293T cells as they possess a wild-type *MUTYH* gene [63, 36], are easy to maintain and transfect, and have been used previously for MUTYH activity studies [43–45, 64, 65]. We transfected HEK293T cells with plasmids expressing either ancBE4max-NG (a CBE, hereafter referred to as BE4) [66, 67] or ABE7.10max-NG (an ABE, hereafter referred to as ABE7.10) [68] and a custom-designed gRNA (Supplementary Fig. S5A), allowed 3 days for editing to occur, lysed the cells, amplified the *MUTYH* loci of interest, and sequenced the resulting amplicons with Sanger sequencing to assess bulk editing efficiencies and precision (Supplementary Fig. S5B). We proceeded to generate isogenic cell lines using the ten BE:gRNA combinations indicated in Supplementary Fig. S4 (gray rows), as these introduced the desired mutations with high editing efficiencies (see Supplementary Fig. S6 for bulk editing traces of BE:gRNA combinations with no or low editing efficiencies).

**Figure 2.**
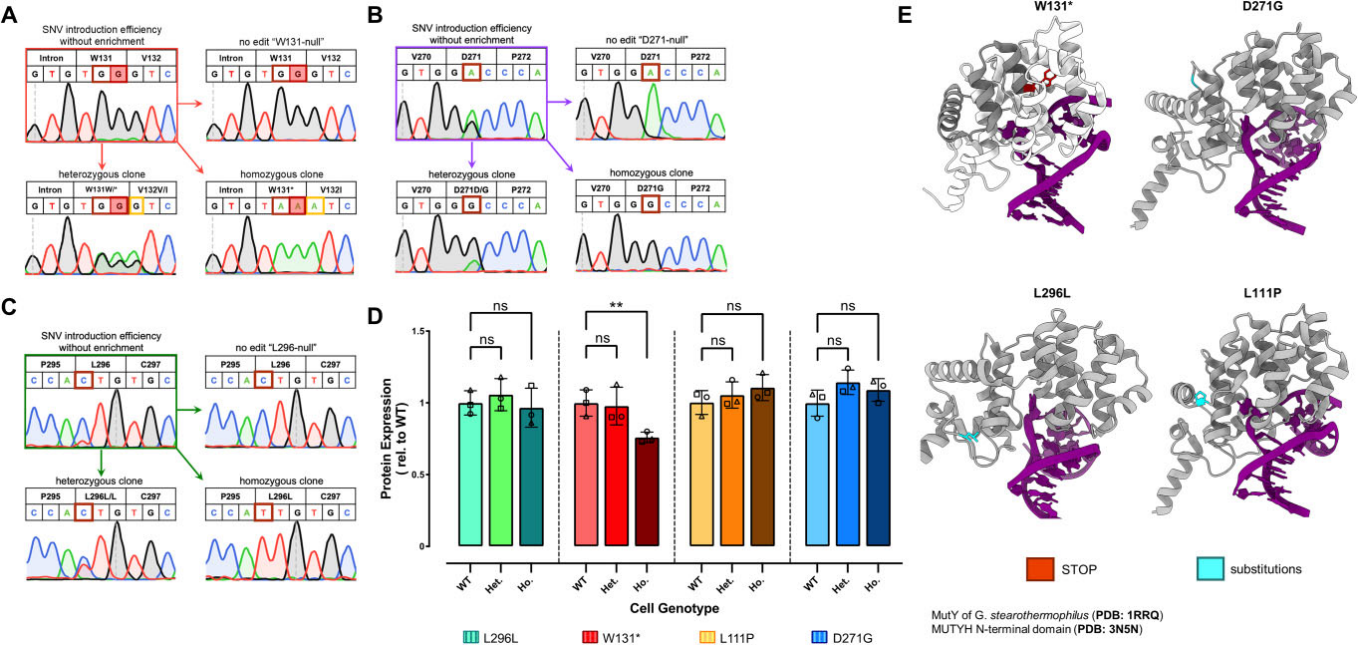
Generation of isogenic cell lines harboring clinically relevant *MUTYH* SNVs. (**A–** **C**) HEK293T cells were subjected to the work flow shown in Supplementary Fig. S5. Shown are Sanger sequencing traces of the SNV loci after bulk editing (top left) and after isogenic cell line generation, with one representative isogenic cell line per genotype (null/unedited, heterozygous, and homozygous). See Supplementary Fig. S5 for the L111P mutation. For sequencing data of all 36 lines (three per genotype, for each of the four SNVs), see Supplementary Figs S8–S15. Note for the W131* variant, the intended target nucleotide is indicated by shading. (**D**) Quantification of western blot data (from Supplementary Figs S20–S22) of isogenic cell line lysate for MUTYH. Bars represent the grand average of *n* = 2 technical replicates of *n* = 3 biological replicates (each data point represents the average of two technical replicates for each clone, with circles showing clone 1, triangles showing clone 2, and squares showing clone 3). Error bars represent the standard deviation of the grand averages of the three biological replicates repeated in duplicate experiments. Data were analyzed with unpaired, one-tailed, parametric *t*-tests (in which the heterozygous or homozygous lines were compared to their wild-type counterparts), and *P*-values are marked as follows: ns = *P* ≥ .05, not significant; *P ≤ .05, and **P ≤ .01 are significant. (**E**) Mutants of interest in this study and their location within the N-terminal domain. The structures were generated by superimposing the crystallized N-terminal domain of MUTYH (PDB: 3N5N) and the MutY bacterial homolog structure (PDB: 1RRQ). The DNA harboring an 8-oxoG•A substrate is also shown. SNVs leading to amino acid changes and the premature stop codon for W131* are indicated. For W131*, amino acids after the STOP codon are transparent.

We then again transfected HEK293T cells with the ten BE:gRNA combinations identified above, allowed 3 days for editing to occur, used FACS to plate one transfected cell (EGFP is co-expressed with the BE) per well in a 96-well plate format, and allowed the cells to expand and generate clonal colonies (Supplementary Fig. S5C). We then lysed a subset of cells for each established clone, and the *MUTYH* loci of interest were sequenced with NGS and/or Sanger sequencing to evaluate the genotype of each clone (Supplementary Fig. S5D). We did not move forward with six of the BE:gRNA combinations, as we observed editing at one or more bystander bases in addition to the target base (Supplementary Fig. S7). We proceeded with the W131* variant despite bystander edits (one of which was in the W131 codon but retained its identity as a stop codon, and one of which caused a conservative V132I substitution in the neighboring codon, Supplementary Fig. S8), as this variant was initially included as a knockout control, and we reasoned that additional mutations past the stop codon would not impact knockout. We successfully generated a full suite of isogenic cell lines for the four *MUTYH* variants listed in Fig. 2: L296L (c.802C > T), W131* (c.309G > A), L111P (c.248T > C), and D271G (c.728A > G). Note that we are using the MUTYH amino acid numbering scheme of isoform 5, as this isoform includes all possible amino acids (see Supplementary Sequences and Fig. 1C) [29]. This numbering system differs from that of isoform 4 (the most abundant nuclear isoform, which is used in ClinVar) by 28 for the majority of the protein (Fig. 1C). The L296L variant has been detected eight times in patients, with seven likely benign interpretations and one VUS interpretation. The D271G variant has been observed in one patient and is a VUS. This mutation occurs at the interface of the catalytic and IDC domains (Figs 1B and 2E). As mentioned previously, the W131* variant was included as a knockout control, despite a neighboring codon bystander mutation. The W131* single G > A point mutation (Fig. 2A, red highlighted base) has been detected six times in patients, with four pathogenic and two likely pathogenic classifications. Finally, the L111P variant has been detected twice with both being classified as likely pathogenic. This mutation occurs in the middle of the catalytic domain (Figs 1B and 2E). Figures 1B and 2E demonstrate the location of these N-terminal amino acid changes within the MUTYH protein.

We proceeded with these variants as we successfully generated three wild-type (null) genotype clones, three heterozygous genotype clones, and three homozygous genotype clones for each. Null clones were those subjected to the base editing workflow and clonal expansion but resulted in no editing at the *MUTYH* locus, thus serving as wild-type controls. The sequencing validation for all 36 isogenic clonal cell lines is shown in Supplementary Figs S8–S15. Because relative peak heights of Sanger sequencing data at mixed-base sites (such as SNVs) are not quantitatively representative of the proportion of each base [69], we performed NGS to quantify the relative number of alleles that were wild-type versus mutant for each heterozygous cell line. Genotyping of the heterozygous clones through NGS revealed each genotype (wild-type or mutant) to be either roughly 33% (ranging from 22% to 36%, which we define as “one allele”) or 66% (ranging from 64% to 78%, which we define as “two alleles”), suggesting three *MUTYH* alleles (chromosome 1 p34.3–p32.1). These allele ratio thresholds of 33% and 66% were defined based on karyotype experiments on HEK293T cells, which report three copies of this allele, consistent with these sequencing data [70]. In our interpretations, we define cells as heterozygous if at least one allele copy of *MUTYH* was unedited while one allele was edited. Therefore, for the W131* variant, clone 1 possesses two edited alleles and one unedited allele, whereas clones 2 and 3 possess only one edited allele and two unedited alleles (Supplementary Fig. S9). For the L111P heterozygous variant, all three clones contain two unedited alleles and one edited allele (Supplementary Fig. S11). For the D271G heterozygous variant, all three clones contain two edited alleles and one unedited allele (Supplementary Fig. S13). For the L296L heterozygous variant, all three clones contain two unedited alleles and one edited allele (Supplementary Fig. S15). We will note there is always the possibility that some heterozygous clones may be a mixture of heterozygous and homozygous cells. However, given our later experiments demonstrate only the homozygous clones to differ phenotypically from wild-type, we believe this to be unlikely. All homozygous clones demonstrated all *MUTYH* alleles to be edited.

To control for “background” mutations that may have resulted from the clonal expansion processes (in which case, mutations would occur in different locations in different clones), we established three independent cell lines for each genotype. Each cell line also served as biological replicates for all experiments. We additionally analyzed our cell lines for potential off-target base editing, in which case mutations would occur in in the same sites (those with homology to the gRNA spacer sequence) across multiple clones and may convolute interpretation of mechanistic results. To do this, we identified the top two coding and two noncoding predicted off-target sites per gRNA (listed in Supplementary Table S4) using the algorithm developed by Hsu *et al.* and sequenced these loci in each of our 36 isogenic cell lines [71, 72]. This algorithm identifies sequences in the human genome with homology to the on-target site and assigns an off-target score to each one that correlates with the likelihood of editing occurring at that site (ranging from 0 to 100, where 100 is a perfect match). These off-target scores take into account the number, location within the spacer, and identity of mismatches between the putative off-target site and the gRNA used. Notably, we observed no off-target mutations at any of these sites (Supplementary Figs S16–S19). Overall, these data demonstrate our ability to use base editing to obtain both homozygous and heterozygous isogenic models of *MUTYH* SNVs with no significant off-target mutations.

### Analysis of protein expression levels in isogenic cell lines

We next evaluated the expression levels of the MUTYH protein in the isogenic cell lines by performing western blot analyses. All cell lines except the homozygous W131* mutant showed MUTYH expression levels within error or slightly higher than unedited HEK293T cells (Fig. 2D and Supplementary Figs S20–S22). In the homozygous W131* mutant cell lines, we observed an average 24.1 ± 4.3% reduction in MUTYH protein levels (Supplementary Figs S20–S21). We expected to observe reduced protein levels in the heterozygous cell lines and a complete knockout of protein levels in the homozygous cell lines due to nonsense-mediated mRNA decay (NMD) from the premature termination codon. Indeed, the ClinVar database proposes that this variant is pathogenic due to NMD of the *MUTYH* transcript [73], which is based on studies of other frameshifting variants in *MUTYH* [40, 74]. However, not only did we detect MUTYH protein in these lines, but the molecular weight of the detected protein is consistent with full-length MUTYH (Supplementary Fig. S21). This suggests that the mechanism of pathogenicity of the W131* *MUTYH* variant may be independent of protein truncation or knockout, although functional investigation in additional cell types would be necessary to confirm this.

Given the lack of protein knockout of the W131* homozygous line, we elected to generate an additional homozygous line with a more significant perturbation to the MUTYH protein. We used the algorithm developed by Doench *et al.* [71] to select a gRNA with a high predicted on-target score that targeted the gene in a central exon. We paired this gRNA with wild-type Cas9 and repeated the transfection and clonal expansion process, aiming to knockout *MUTYH* via frameshifting insertion or deletion sequences (indels). We obtained three clones that were observed to be edited at all three alleles and used NGS to sequence the locus of interest (Supplementary Fig. S23A). While all three clones were confirmed to possess three edited alleles, at least one chromosome in each case harbored a deletion that was a multiple of three. Due to this possibility, we had targeted the gRNA to a location within *MUTYH* that encodes for amino acids 107–108, an essential region in MUTYH that plays a role in DNA binding and catalysis [75]. In this manner, we would expect the resulting mutations to significantly impact the overall structure and/or function of MUTYH despite not knocking out the gene. We found clone 1 to have two alleles with a 15-bp deletion (corresponding to a deletion of amino acids 106–110) and one allele with a 13-bp deletion, clone 2 to have one allele with the same 15-bp deletion, one allele with the same 13-bp deletion, and one allele with a large (∼200 bp) insertion, and clone 3 to have one allele with an 18-bp deletion (corresponding to a deletion of amino acids 104–109), one allele with a 1-bp deletion, and one allele with a 7-bp deletion (Supplementary Fig. S23A and B). Consequently, following western blot analysis, we found all three deletion clones (which we call *MUTYH*Δ) to still possess MUTYH protein at similar levels to wild-type HEK293T cells (Supplementary Fig. S23C). Nevertheless, with these cell lines in hand, we next sought to develop an assay to evaluate the DNA repair capacity of each MUTYH mutant in its endogenous context.

### Constructing fluorescent reporter plasmids to measure repair of 8-oxoG•A, 8-oxoG•C, and 8-oxoG•[O] within live cells

**Figure 3.**
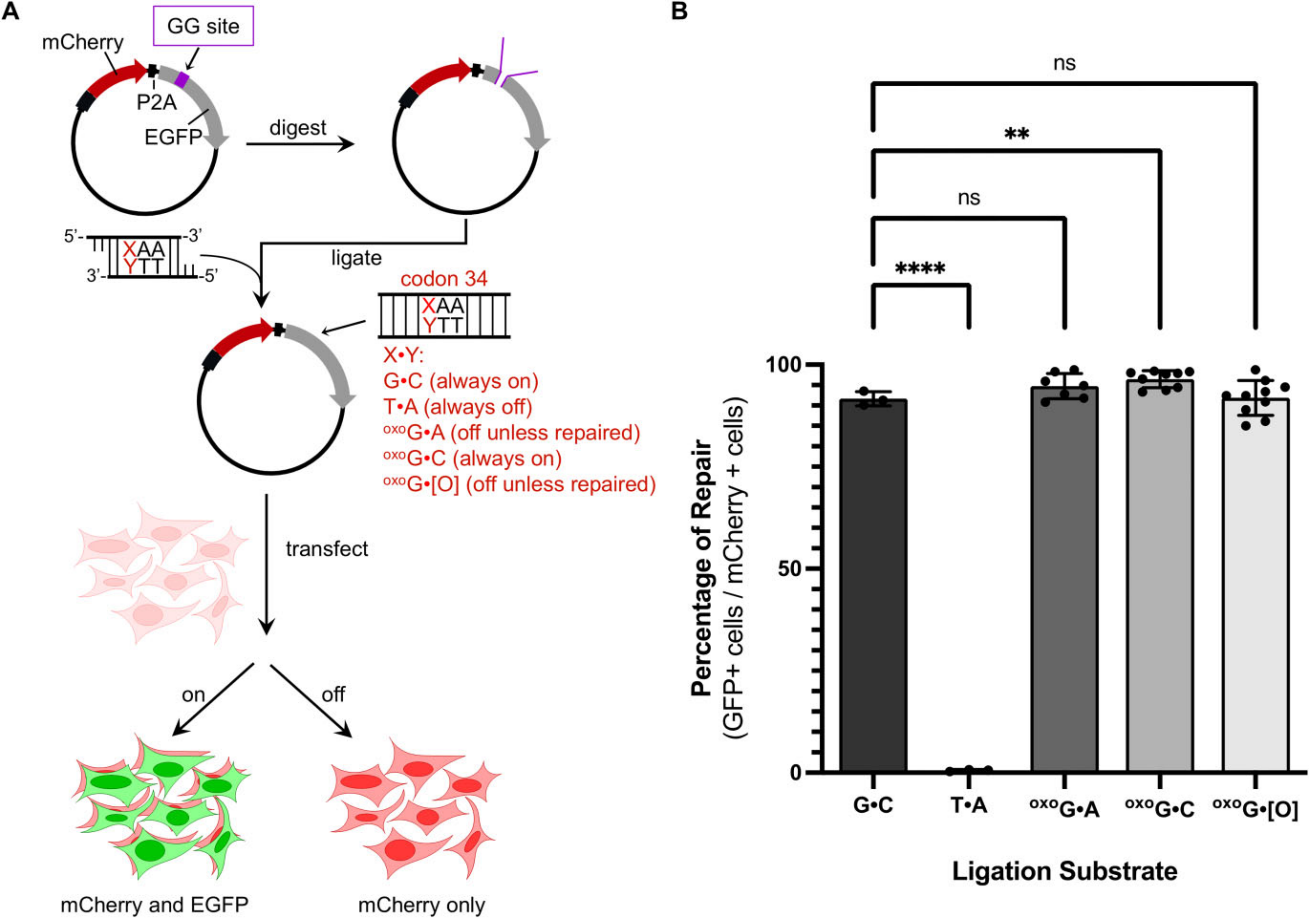
Validation of 8-oxoG•A and 8-oxoG•[O] lesion-specific plasmid reporters. (**A**) Schematic diagram of generation and use of lesion-containing plasmids. The plasmid-based fluorescent reporters contain both a red fluorescent protein, mCherry, and an inactive EGFP. EGFP is inactive due to a sequence of DNA that frameshifts the EGFP (Supplementary Fig. S24) and harbors an incorporated dual Type IIS restriction enzyme (BsaI) recognition site (referred to as a Golden Gate site or GG site), which allows restriction digestion to produce custom sticky ends (Supplementary Fig. S24). This construct enables various inserts with compatible sticky ends to be ligated into the digested plasmid backbone and produce an intact mCherry-P2A-EGFP plasmid with custom base-pairs at codon 34 of EGFP. To evaluate MUTYH DNA repair activity, an 8-oxoG•A or 8-oxoG•[O] lesion is incorporated into codon 34. Control plasmids in which G•C, T•A, or 8-oxo-G•C are incorporated into codon 34 were generated as well. Once transfected into live cells harboring either WT or mutated *MUTYH*, both genes are transcribed in a single mRNA transcript, but are translated into unique and separate fluorescent proteins via the self-cleaving P2A linker. mCherry fluorescence acts as a transfection marker, and EGFP fluorescence occurs only there is repair of 8-oxoG•A or 8-oxoG•[O] to 8-oxoG•C or G•C by MUTYH and downstream BER proteins. (**B**) Percentage of repair in living cells harboring wild-type *MUTYH* for the five different base pairs indicated in (A) are shown. Repair is quantified by calculating the percent of EGFP+ cells divided by the transfected, or mCherry+, cells. Bars represent the average of *n* = 3–10 biological replicates. Error bars represent the standard deviation of the biological replicates. Data were analyzed with unpaired, one-tailed, parametric *t*-tests (compared to the G•C control sample), and *P*-values are marked as follows: ns = *P* ≥ .05, not significant; **P ≤ .01, and ****P ≤ .0001 are significant.

Next, we sought to develop a method to quantify the enzymatic activities of the MUTYH mutants within their native cellular environments. As mentioned, WT MUTYH recognizes the 8-oxoG•A lesion and excises the A to produce an abasic site (8-oxoG•[O]). It then coordinates with downstream BER proteins (most notably APE1) to convert this lesion into 8-oxoG•C (Fig. 1A). To quantify 8-oxoG•A repair by MUTYH in our isogenic cell lines, we adapted a previously described reporter system to measure the DNA repair activity of overexpressed MUTYH using flow cytometry [43]. We generated an mCherry-P2A-EGFP construct in which mCherry and EGFP are transcribed on the same mRNA transcript but translated into separate proteins due to ribosomal skipping of the P2A linker during translation. Within the EGFP gene, we incorporated dual Type IIS restriction enzyme (BsaI) sites (which we call a Golden Gate site or GG site), which allow restriction digestion of the plasmid to produce custom sticky-ends (Supplementary Fig. S24A and B). This construct enabled us to ligate various inserts with compatible sticky ends into the digested backbone and produce an intact mCherry-P2A-EGFP plasmid with custom base pairs at codon 34 (Supplementary Fig. S24C–E). In particular, an 8-oxoG•A mismatch could be incorporated at codon 34 to generate a non-fluorescent EGFP protein with a premature stop codon at position 34 (E34*, Fig. 3A). Notably, the 8-oxoG lesion would be on the coding strand, with the mispaired A base on the template strand (Fig. 3A). Therefore, repair of the 8-oxoG•A to 8-oxoG•C by MUTYH and downstream BER proteins (or back to G•C by MUTYH, OGG1, and downstream BER proteins) would restore EGFP fluorescence, with mCherry fluorescence reporting on transfection efficiency (Fig. 3A). Additionally, the plasmid does not contain a mammalian-compatible origin of replication, so DNA replication does not interfere with the assay.

As positive and negative controls, we cloned mCherry-P2A-EGFP constructs with either Glu34 (wild-type EGFP; pCAV033, Supplementary Fig. S25A) or Stop at codon 34 (pCAV034, Supplementary Fig. S25B). We transfected either plasmid into wild-type HEK293T cells, waited 24 h, and analyzed the cells by the flow cytometry. As expected, 99.4 ± 0.5% of transfected cells (as determined by cells with mCherry fluorescence above background levels) were EGFP+ when using the Glu34 plasmid (Supplementary Fig. S25E), and only 0.07 ± 0.13% of transfected cells were EGFP + when using the Stop34 plasmid (Supplementary Fig. S25F), suggesting our strategy for using EGFP fluorescence to differentiate C (repaired) versus A (unrepaired) on the template strand was viable. We also transfected wild-type HEK293T cells with the GG site-containing plasmid (pCAV035, Addgene plasmid #219807, Supplementary Fig. S25C), as well as the BsaI-digested pCAV035 plasmid (Supplementary Fig. S25D) and analyzed cells by flow cytometry after 24 h. We observed 0.05 ± 0.05% (pCAV035, Supplementary Fig. S25G) and 0.06% (digested pCAV035, Supplementary Fig. S25H) of transfected cells to be EGFP+ in these two samples, demonstrating that background EGFP fluorescence from either DNA sample is negligible.

Next, we took digested pCAV035 plasmid and ligated four different inserts into the digested site (Fig. 3A): one containing a T•A base-pair, which would match the pCAV034 Stop34 plasmid sequence (Supplementary Fig. S26A), one containing a G•C base-pair, which would match the pCAV033 Glu34 plasmid sequence (Supplementary Fig. S26B), one containing 8-oxoG•C, which would serve as a positive control because C is on the template strand (Supplementary Fig. S26C), and one containing a 8-oxoG•A lesion, which would be a substrate for MUTYH (Supplementary Fig. S26D). Each of these four DNA constructs was transfected into wild-type HEK293T cells, which were then analyzed by flow cytometry after 24 h. The results from the T•A- and G•C-containing base-pairs matched that of their corresponding intact plasmids; only 0.13 ± 0.16% of transfected cells were EGFP+ when using the T•A-containing construct (Fig. 3B and Supplementary Fig. S26F), while 93.7 ± 3.5% of transfected cells were EGFP + when using the G•C-containing construct (Fig. 3B and Supplementary Fig. S26G). Additionally, 96.5 ± 2.2% of transfected cells were EGFP+ when using the 8-oxoG•C-containing construct (Fig. 3B and Supplementary Fig. S26H), and 95.8 ± 3.1% of transfected cells were EGFP+ when using the 8-oxoG•A-containing construct (Fig. 3B and Supplementary Fig. S26I), suggesting efficient repair of 8-oxoG•A back to either 8-oxoG•C or G•C by the endogenous MUTYH and downstream BER proteins. To further validate the reporters, we repeated the 8-oxoG•A and 8-oxoG•C experiments using the *MUTYH*Δ cell lines (Supplementary Fig. S27A and B). In this case, 98.4 ± 0.7% of transfected cells were EGFP+ when using the 8-oxoG•C-containing construct (Supplementary Fig. S27D and G), while only 57.0 ± 8.1% of transfected cells were EGFP+ when using the 8-oxoG•A-containing construct (Supplementary Fig. S27E and H), demonstrating the ability of this strategy to report on MUTYH-specific repair capacity in live cells. In addition to a decrease in the absolute percentage of transfected cells with EGFP fluorescence, we observed a decrease in the median fluorescence intensity (MFI) of EGFP+ cells when using the 8-oxoG•A-containing construct (but not the 8-oxoG•C construct); namely, we observed EGFP+ cells to have an average MFI of 0.09 ± 0.01 in the *MUTYH*Δ samples relative to that of unedited HEK293T cells (Supplementary Fig. S27K). These data suggest that in cells with EGFP signal, fewer 8-oxoG•A lesions are being repaired in the *MUTYH*Δ cells.

Finally, we used this same strategy to site-specifically incorporate an 8-oxoG•[O] lesion (where [O] represents an abasic site, Supplementary Fig. S26E). MUTYH is a monofunctional DNA glycosylase and thus only catalyzes the excision of the adenine opposite the damaged 8-oxoG lesion to produce the 8-oxoG•[O] intermediate. APE1 is then required to cleave the DNA backbone prior to replacement with C. Notably, wild-type MUTYH has an affinity for this lesion in addition to its canonical 8-oxoG•A substrate [48]. We therefore reasoned that EGFP fluorescence levels in cells transfected with the 8-oxoG•[O] substrate might report on how well MUTYH coordinates downstream repair of 8-oxoG•A after it enzymatically processes the lesion to excise A. Specifically, mutants that are defective at interacting with APE1 (or any other MUTYH binding partners) would have reduced abilities to repair the 8-oxoG•[O] lesion. Transfection of this lesion-containing DNA construct into wild-type HEK293T cells followed by the flow cytometry analysis showed high levels of EGFP fluorescence; 91.8 ± 4.3% of transfected cells were EGFP+ (Fig. 3B and Supplementary Fig. S26J). When we repeated this experiment with the *MUTYH*Δ cells, we did not observe a significant decrease in the percent of transfected cells with EGFP fluorescence but did observe a significant decrease in the MFI of EGFP+ cells compared to wild-type (0.55 ± 0.05-fold compared to unedited HEK293T cells, Supplementary Fig. S27F, I, and L). Notably, while versions of this assay have been used with 8-oxoG•A and several 8-oxoG analogs [76], it has never been used to measure repair of the 8-oxoG•[O] intermediate before, which can provide mechanistic details of how pathogenic *MUTYH* variants are defective at DNA repair. Having developed and validated these reporters for MUTYH activity, we next sought to use them to directly evaluate MUTYH-mediated repair in in our isogenic cell lines.

### Evaluation of 8-oxoG•A repair activities of MUTYH mutants within live cells using a fluorescent reporter

We first transfected the 36 *MUTYH* variant isogenic cell lines with the 8-oxoG•C reporter, which we expected should show similarly high levels of EGFP fluorescence across all cell lines. After 24 h, cells were analyzed by flow cytometry (Supplementary Fig. S28). As expected, all cell lines had similar levels of transfected cells with EGFP fluorescence to the parental, unedited HEK293T cells (96.5 ± 2.2%, Fig. 3B). Furthermore, the MFI of EGFP + cells was within error of that of unedited HEK293T cells for all cell lines (Supplementary Fig. S28E–H).

**Figure 4.**
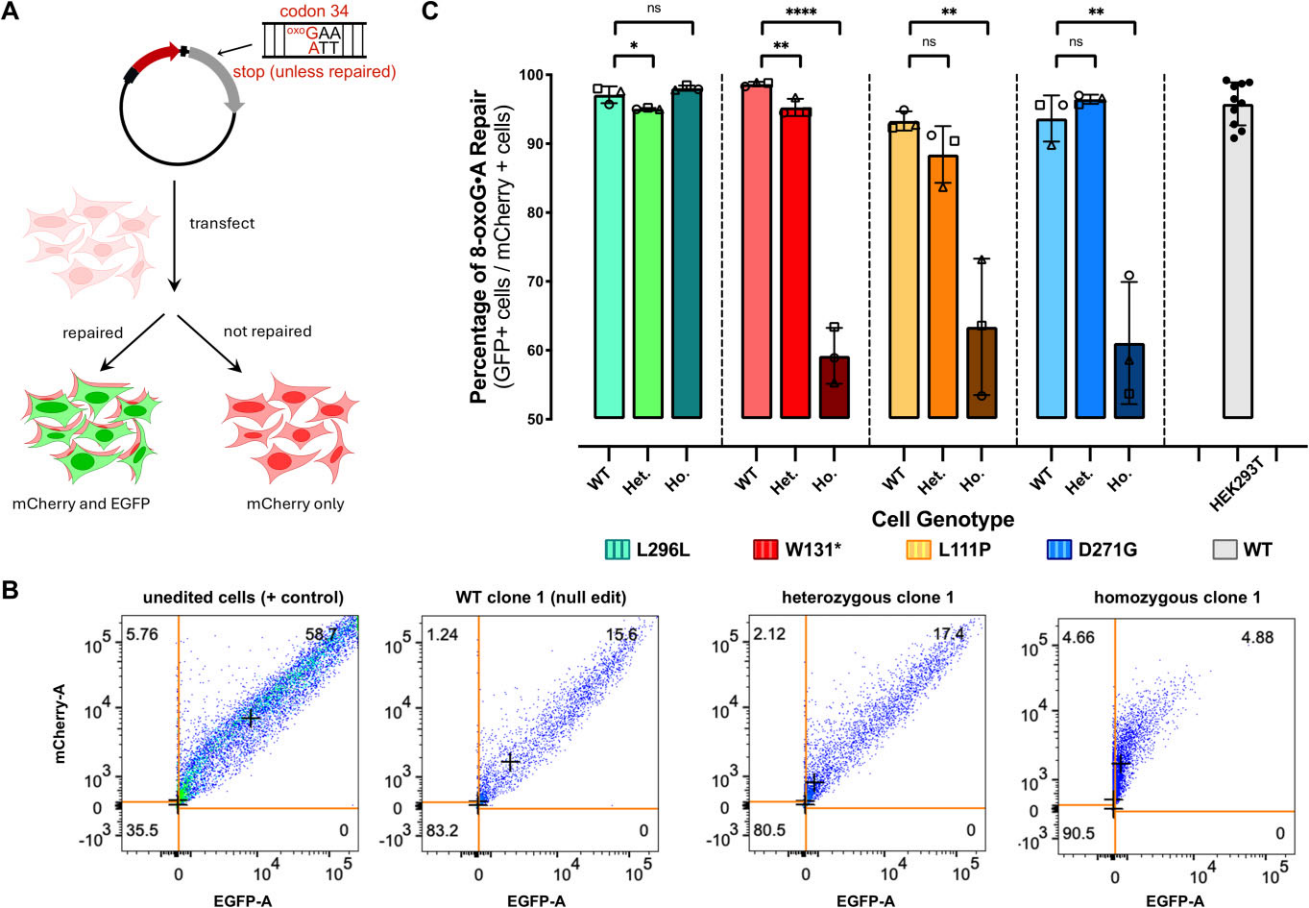
Repair of 8-oxoG•A using a MUTYH lesion-specific plasmid reporter. (**A**) Schematic diagram of the fluorescent reporter for 8-oxoG•A repair. (**B**) Representative flow cytometry plots and gating schemes to quantify 8-oxoG•A repair in live cells using matching L111P MUTYH mutant cell lines as an example. The plots show compensated red fluorescence intensity (*y*-axis) versus compensated EGFP fluorescence intensity (*x*-axis) for four representative samples, left to right: unedited HEK293T cells, HEK293T cells that were transfected with the L111P gRNA, but produced no editing at the target site (null clone), heterozygous L111P MUTYH clone 1, and homozygous L111P MUTYH clone 1. 8-oxoG•A repair is quantified by calculating the percent of EGFP+ cells divided by the transfected, or mCherry+, cells. Scatter gates were applied to remove nonviable cells and doublets as shown in Supplementary Fig. S2. Quadrant boundaries for analysis were set by using unedited HEK293T cells that were transfected with mCherry only or EGFP only plasmids. The numbers in each quadrant represent the percentage of cells within that population. “+’’s in the quadrants indicate the median EGFP fluorescence intensity of EGFP-positive cells. (**C**) Percentage of 8-oxoG•A repair in living cells harboring various MUTYH mutants. Values calculated as described in (B). Bars represent the average of *n* = 3 biological replicates (circles show clone 1, triangles show clone 2, and squares show clone 3). Error bars represent the standard deviation of the three biological replicates. Data were analyzed with unpaired, one-tailed, parametric *t*-tests (in which the heterozygous or homozygous lines were compared to their wild-type counterparts), and *P-*values are marked as follows: ns = *P* ≥ 0.05, not significant: *P ≤ .05, **P ≤ .01, and ****P ≤ .0001 are significant.

The 36 individual isogenic cell lines were then transfected with the 8-oxoG•A fluorescent reporter (Fig. 4A). After 24 h, cells were imaged by fluorescence microscopy and 8-oxoG•A repair was quantified by flow cytometry (Fig. 4B and Supplementary Fig. S29). The L296L cell lines behaved similarly to the parental, unedited HEK293T cells, as expected given the L296L clinical significance of “benign.” Specifically, we observed an 8-oxoG•A repair efficiency (which we define as the percent of transfected, or mCherry+, cells with EGFP fluorescence) of 97.1 ± 1.2% for the WT (null) clones, 95.1 ± 0.12% for the heterozygous clones, and 98.1 ± 0.4% for the homozygous clones (Fig. 4C and Supplementary Fig. S29A). In contrast, we observed a noticeable decrease in 8-oxoG•A repair activity for the homozygous W131* (pathogenic) clones. Specifically, we observed an 8-oxoG•A repair efficiency of 98.7 ± 0.3% for the WT (null) clones, 95.3 ± 1.2% for the heterozygous clones, and 59.2 ± 4.1% for the homozygous clones (Fig. 4C and Supplementary Fig. S29B), representing a 38% decrease compared to the parental, unedited HEK293T cells. Notably, this decrease is similar to what we observed with the *MUTYH*Δ clones, in which MUTYH is significantly perturbed (Supplementary Fig. S27H). This decrease in repair activity is also greater than the decrease in protein expression levels for these cell lines, suggesting a mechanism of repair deficiency involving more than just decreased protein expression levels for this variant. Interestingly, the heterozygous genotype behaved similarly to the parental, unedited HEK293T cells and the WT (null) line, which supports reports that MUTYH-associated cancers are autosomal recessive. The L111P (pathogenic) clones had a similar phenotype to the W131* clones, with an 8-oxoG•A repair efficiency of 93.3 ± 1.4% for the WT (null) clones, 88.4 ± 4.1% for the heterozygous clones, and 63.4 ± 9.9% for the homozygous clones (Fig. 4B and Supplementary Fig. S29C), representing a 34% decrease compared to the parental, unedited HEK293T cells. Again, the WT (null) and heterozygous clones behaved similarly to each other. Finally, when measuring 8-oxoG•A repair for the D271G (VUS) clones, we observed similar 8-oxoG•A repair efficiencies to both pathogenic variants. Specifically, we observed 8-oxoG•A repair efficiencies of 95.9 ± 0.6% for the WT (null) clones, 94.2 ± 3.8% for the heterozygous clones, and 61.1 ± 8.9% for the homozygous clones (Fig. 4B and Supplementary Fig. S29D), representing a 36% decrease compared to the parental, unedited HEK293T cells. Once again, the heterozygous clones had a similar phenotype to their WT (null) counterparts. In all cases where we observed large reductions in 8-oxoG•A repair efficiencies, as defined as the percent of transfected cells with EGFP fluorescence, we also observed significantly reduced MFIs of EGFP+ cells (Supplementary Fig. S29E–H). These data demonstrate the utility of this fluorescent reporter strategy for quantifying the repair capacity of MUTYH variants in live cells. Furthermore, while the sample size is small, we observed reduced MUTYH repair capacity to correlate with clinical pathogenicity.

### Reductions in 8-oxoG•[O] repair efficiency for the W131* and D271G MUTYH mutants suggest defective interactions with downstream BER proteins

**Figure 5.**
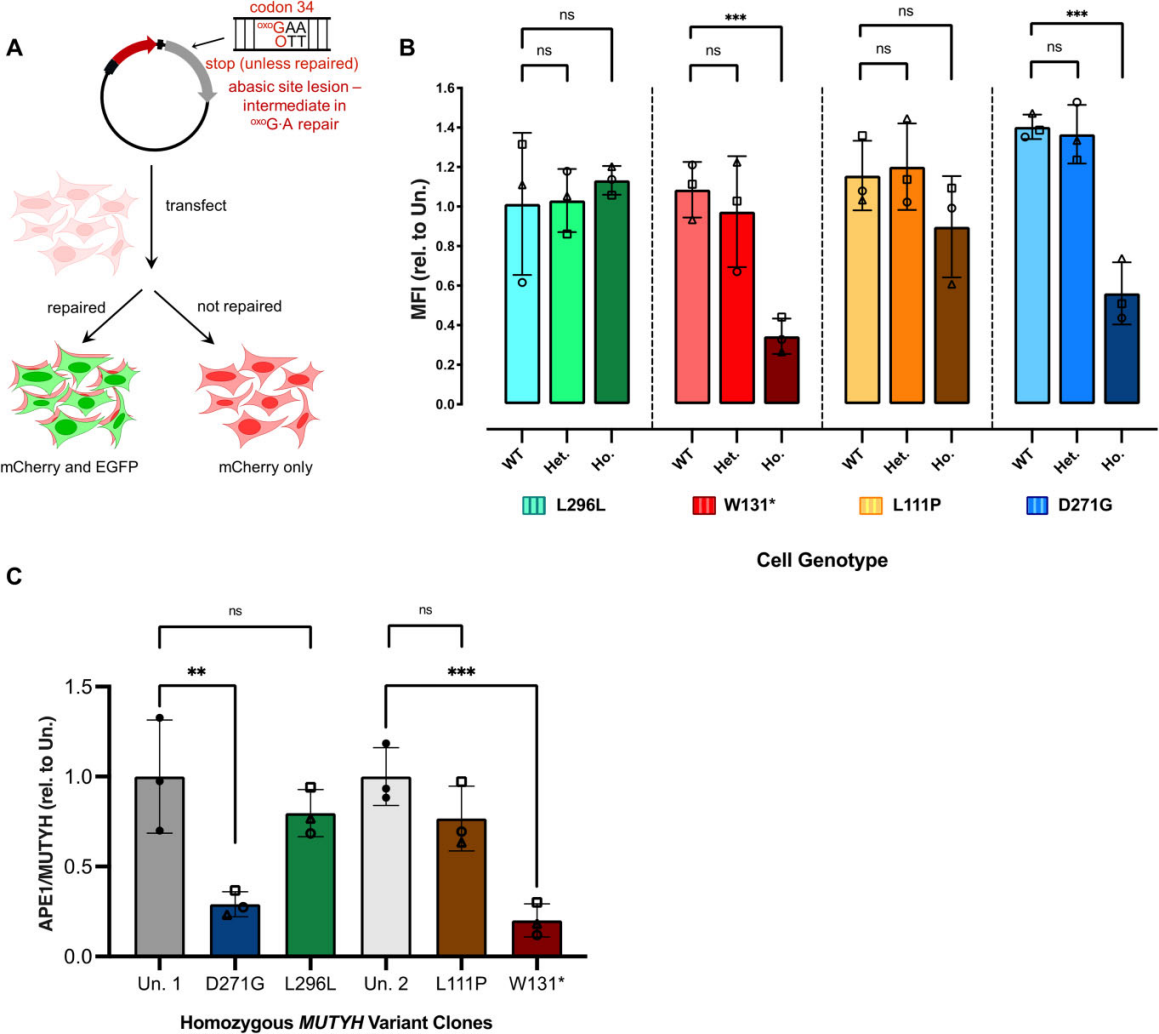
Defective MUTYH-APE1 Interactions Identified by the 8-oxoG•[O] reporter and co-IP experiments. (**A**) Schematic diagram of the fluorescent reporter for 8-oxoG•[O] repair. (**B**) Relative 8-oxoG•[O] repair values in living cells harboring various MUTYH mutants. Values calculated as described in Supplementary Fig. S27J–L. Bars represent the average of *n* = 3 biological replicates (circles show clone 1, triangles show clone 2, and squares show clone 3). Error bars represent the standard deviation of the three biological replicates. Data were analyzed with unpaired, one-tailed, parametric *t*-tests (in which the heterozygous or homozygous lines were compared to their wild-type counterparts), and *P-*values are marked as follows: ns = *P* ≥ .05, not significant; *P ≤ .05, **P ≤ .01, and ***P ≤ 0.001 are significant. (**C**) Plotted are the ratios of the relative amount of APE1 protein to total MUTYH protein in MUTYH immunoprecipitated samples, normalized to that of untreated HEK293T cells (based on the co-IP experiments in Supplementary Fig. S30). Plotted for each experimental condition are *n* = 3 biological replicates containing homozygous genotypes (circles show clone 1, triangles show clone 2, and squares show clone 3). The D271G and L296L homozygous clones were normalized to the average of untreated HEK293T cells from the same blot (Un.1), and L111P and W131* homozygous clones were normalized to the average of untreated HEK293T cells on the same blot (Un.2). Error bars represent the standard deviation of the three biological replicates. Data were analyzed with unpaired, one-tailed, parametric *t*-tests, and *P*-values are marked as follows: ns = *P* ≥ .05, not significant; *P ≤ .05, **P ≤ .01, and ***P ≤ .001 are significant.

To further characterize the DNA repair capacities of our *MUTYH* variants, we next transfected all 36 isogenic lines with the 8-oxoG•[O] fluorescent reporter (Fig. 5A). Again, after 24 h, cells were imaged by the fluorescence microscopy, and 8-oxoG•[O] repair was quantified by flow cytometry. The L296L cell lines again behaved similarly to the parental, unedited HEK293T cells. Specifically, we observed 8-oxoG•[O] repair efficiencies (which we define as the average fold-change in MFI of EGFP+ cells compared to that of unedited HEK293T cells) of 1.01 ± 0.36 for the WT (null) clones, 1.03 ± 0.16 for the heterozygous clones, and 1.13 ± 0.07 for the homozygous clones (Fig. 5B). In the W131* cell lines, we observed 8-oxoG•[O] repair efficiencies of 1.09 ± 0.14 for the WT (null) clones, 0.97 ± 0.16 for the heterozygous clones, and 0.34 ± 0.09 for the homozygous clones (Fig. 5B), indicating a deficiency in coordination of downstream BER for this variant. The L111P mutation within *MUTYH* is near the enzyme’s active site and is predicted by *in silico* methods to be defective in adenine excision [77]. Correspondingly, for the L111P cell lines, we observed 8-oxoG•[O] repair efficiencies of 1.16 ± 0.18 for the WT (null) clones, 1.20 ± 0.21 for the heterozygous clones, and 0.90 ± 0.26 for the homozygous clones (Fig. 5B). This indicates that while this variant is overall deficient in 8-oxoG•A repair, the deficiency is likely at the adenine excision step, as it is able to coordinate repair of the 8-oxoG•[O] intermediate. Finally, for the D271G cell lines, we observed 8-oxoG•[O] repair efficiencies of 1.40 ± 0.06 for the WT (null) clones, 1.37 ± 0.15 for the heterozygous clones, and 0.56 ± 0.16 for the homozygous clones (Fig. 5B), indicating a deficiency in coordinating 8-oxoG•[O] repair by this variant. This suggested to us that the mechanism of 8-oxoG•A repair deficiency of the D271G mutant involves its inability to interact with downstream BER proteins, while the L111P mutant is proficient in coordinating downstream repair of the 8-oxoG•[O] intermediate. Notably, these data are consistent with the respective locations of each mutation (D271G near the IDC, and L111P in the catalytic domain, Fig. 1C). Furthermore, these data demonstrate that this assay is able to report on the capacity of MUTYH to coordinate downstream repair of its substrate following adenine excision.

### Defective MUTYH-APE1 interactions identified by coimmunoprecipitation

The W131* and D271G MUTYH mutants were defective at repair of both 8-oxoG•A and 8-oxoG•[O] lesions, which we hypothesized to be due to deficiencies in interacting with APE1. Notably, the binding site for APE1 has been reported to reside within the IDC of MUTYH (amino acids 309–364) [33], which is close to the D271G mutation. We therefore sought to probe the interaction of MUTYH with APE1 in our cell lines. We examined whether each of our four mutations in MUTYH impacted the corresponding protein’s ability to interact with APE1 by co-IP experiments. Since co-IP requires robust and durable protein association, we temporarily induced elevated (but still physiologically relevant) expression levels of MUTYH by incubating cells (wild-type and homozygous mutant lines) with hydrogen peroxide for an hour to induce oxidative stress [78]. We then performed a nuclear extraction, immunoprecipitated MUTYH from the resulting nuclear lysate, and blotted for APE1. The co-IP experiments were consistent with our data from the 8-oxoG•[O] repair assay (Fig. 5C and Supplementary Fig. S30). Specifically, we detected high levels of APE1 in the immunoprecipitants from all wild-type, L296L, and L111P homozygous cell lines (mutants that displayed 8-oxoG•[O] repair activities similar to wild-type, Fig. 5B and C and Supplementary Fig. S30). However, this interaction was greatly reduced in the immunoprecipitants from the W131* and D271G homozygous cell lines (mutants that displayed greatly reduced 8-oxoG•[O] repair activities compared to wild-type, Fig. 5B and C and Supplementary Fig. S30). APE1 signal was completely absent from IgG control samples (Supplementary Fig. S30). Quantification of APE1 signal relative to that of MUTYH, normalized to unedited clones, is shown in Fig. 5C and supports these observations. We observed APE1 levels similar to wild-type in the L296L (0.80 ± 0.13 relative to wild-type levels; not significantly different, *P* = .180) and L111P (0.82 ± 0.19 relative to wild-type levels; not significantly different, *P* = .085) homozygous lines, but this was greatly reduced in the W131* (0.21 ± 0.10 relative to wild-type levels, *P* = .001) and D271G (0.29 ± 0.07 relative to wild-type levels, *P* = .010) homozygous lines. These data support the notion that the 8-oxoG•[O] repair assay reflects upon MUTYH’s ability to coordinate downstream BER of this intermediate. This also demonstrates that the mechanism of pathogenicity of the D271G mutant involves its failure to interact with APE1.

## Discussion

In this work, we leveraged base editing to generate cellular models of clinically-relevant *MUTYH* variants, allowing for the mechanistic study of the corresponding mutant proteins in their native cellular environment. Notably, studying these mutants by introducing their corresponding mutations into the endogenous genomic locus does not impact their native expression levels, resulting in conditions that more closely resemble their natural environment. To evaluate MUTYH-mediated repair of 8-oxoG•A in these cell lines, we adapted a fluorescence-based adenine glycosylase assay, in which excision of the mispaired adenine opposite the 8-oxoG followed by the installation of C restores EGFP expression. This fluorescent reporter allowed us to directly measure the MUTYH repair efficiencies of each mutant compared to wild-type in our cell lines. Furthermore, we modified this assay to also introduce an 8-oxoG•[O] (abasic site-containing) substrate at the lesion site, enabling us to observe MUTYH’s ability to coordinate downstream repair of this intermediate with other BER factors. Together, these assays allowed us to assess overall 8-oxoG•A repair capacity (which correlated with clinical pathogenicity) as well as provided us with mechanistic information regarding each mutant’s repair deficiency. The results from the 8-oxoG•[O] repair assay were then complemented with co-IP studies of MUTYH and APE1. Notably, we found that only homozygous (and not heterozygous) cell lines demonstrated reduced DNA repair capacity, which supports reports that MAP is autosomal recessive.

We used the L296L and W131* mutants as positive and negative controls, respectively. The L296L mutant, which is clinically classified as likely benign, is caused by a silent C > T mutation, while W131*, which is clinically classified as likely pathogenic, is due to a G > A nonsense mutation. Correspondingly, we found that all L296L cell lines (heterozygous and homozygous) behaved as wild-type across all assays, while the homozygous W131* lines were defective at both 8-oxoG•A and 8-oxoG•[O] repair. While we expected this phenotype to be due to protein knockout, we observed only a partial (average 24 ± 4.3%) reduction in MUTYH expression levels in these cell lines. The MUTYH protein expressed in these cell lines appeared to be full-length as well. While additional mechanistic investigation of this phenomenon is beyond the scope of this work, this observation suggests a pathogenic mechanism for this variant that may be distinct from NMD of the *MUTYH* mRNA.

We additionally studied two missense variants, L111P (clinically classified as likely pathogenic) and D271G (clinically classified as VUS). The homozygous cell lines of both mutants had MUTYH protein expression levels within error of wild-type controls, but were found to be defective at 8-oxoG•A and repair. Interestingly, the L111P homozygous cell lines were proficient at 8-oxoG•[O] repair, suggesting that this mutant is able to bind to this substrate and interact with downstream BER proteins. Furthermore, co-IP experiments on the L111P homozygous cell lines showed the MUTYH-APE1 interaction to be intact and comparable to that in wild-type and L296L cell lines. Taken together, these data suggest that the L111P mutant is likely pathogenic due to defective adenine excision of the 8-oxoG•A substrate.

In contrast, the D271G mutant was defective at both 8-oxoG•A and 8-oxoG•[O] repair, suggesting a deficiency of this variant to interact properly with other BER proteins necessary to complete the repair back to 8-oxoG•C. Consistent with these results, co-IP experiments on the D271G homozygous cell lines confirmed that this mutant no longer interacts with APE1. Studies investigating the physical interaction between the two proteins have suggested the binding site for APE1 on MUTYH to be residues 293–318 [31, 33]. While the D271G mutation is outside this region, the highly unconservative nature of this mutation may be responsible for impacting this interaction. Overall, these data suggest a faulty “baton handoff” (miscoordination) between the D271G MUTYH mutant and WT APE1 necessary to process 8-oxoG•A repair within BER. Since the catalytic activity of the D271G mutation may also be impaired, future studies could consider studying this variant *in vitro*, which is beyond the scope of this work.

Currently, over 1000 MAP-associated missense variants are VUS, which will surely rise with the increasing use of sequencing technologies. This limited understanding of *MUTYH* variant dysfunction can be combated by generating relevant human-derived cell models and complementary assays for elucidating their pathogenicity. We developed here a framework for engineering *MUTYH* variant cell lines using base editing, which, prior to this study, had not been reported, and assessed their ability to repair DNA damage in live cells. It is important to note that the generation of isogenic cell lines is quite laborious and requires particularly high genome editing efficiencies and precision. For these reasons, we only proceeded with the functional investigation of four variants. The expansion of the genome editing toolbox since our generation of these cell lines (including more efficient, precise, and flexible BEs, as well as prime editors) alleviates some of these issues and would now reduce some of these barriers. Furthermore, the functional interrogation of variants in high-throughput does not require the generation of isogenic cell lines, and we thus envision high-throughput variant interpretation of MUTYH using base editing to be feasible [79, 80]. It is also important to note the limitations of using HEK293T cells for these studies. While many studies on MUTYH have used HEK293T cells for their practicality, future functional studies may wish to use a colorectal line or at least a cell line that is proficient for mismatch repair (which HEK293T cells are not), as this repair pathway is capable of also recognizing and repairing 8-oxoG•A lesions [81, 82]. Further, the hypotriploid karyotype of HEK293T cells complicate the interpretation of experiments involving heterozygous mutations. While MUTYH-associated variants appear to only impact MUTYH function when homozygous, heterozygous variants involving haploinsufficient genes should not be modeled in this cell type.

Additionally, we modified a previously developed fluorescent reporter for 8-oxoG•A repair activity to also report on repair of the MUTYH intermediate 8-oxoG•[O]. Importantly, we characterized the DNA repair capacity of the D271G mutant, which is currently classified as a VUS and found that this mutation disrupts crucial protein–protein interactions with APE1. These findings underscore the importance of studying potentially pathogenic variants in relevant cell lines in which the full BER pathway is intact. Future applications of this genome editing framework include the clinical and mechanistic characterization of *MUTYH* variants in high-throughput using pooled base editing in combination with this fluorescent reporter and FACS.

## Supplementary Material

gkaf037_Supplemental_File

## Data Availability

Next-generation sequencing data are available on the NCBI Sequencing Read Archive database under project number PRJNA1100199. Reviewers can access these data at https://www.ncbi.nlm.nih.gov/bioproject/PRJNA1100199.

## Notes

Present address: Department of Physical Sciences, Butte College, Oroville, CA 95965, United States

Present address: Department of Medical Oncology, Dana-Farber Center Institute, Boston, MA 02215, United States
