# Supplementary material for: Precision genome editing and in-cell measurements of oxidative DNA damage repair enable functional and mechanistic characterization of cancer-associated MUTYH variants
Source: Nucleic Acids Res. 2025 Mar 28;53(6):gkaf037. doi: 10.1093/nar/gkaf037 (PMC11952967; doi:10.1093/nar/gkaf037)
Supplement: gkaf037_Supplemental_File [file gkaf037_supplemental_file.pdf]

Supplementary Information for

Precision Genome Editing and In-Cell Measurements of Oxidative DNA Damage Repair Enable Functional and Mechanistic Characterization of Cancer-Associated *MUTYH* Variants

Carlos A. Vasquez, Nicola R.B. Osgood, Marcanthony U. Zepeda, Dominika K. Sandel, Quinn T. Cowan, Malalage N. Peiris, Daniel J. Donoghue, Alexis C. Komor

Supplementary Figure 1	Example scatter and sort gates used to generate isogenic cells.
Supplementary Figure 2	Example gating schemes used for DNA repair fluorescent reporters.
Supplementary Figure 3	Overview of base editing.
Supplementary Figure 4	List of MUTYH mutants evaluated for genome editing.
Supplementary Figure 5	Schematic of experimental work-flow for evaluating BE:gRNA combinations and generating isogenic cell lines.
Supplementary Figure 6	Sanger sequencing of bulk editing efficiencies of BE:gRNA combinations with no or low editing of the target base.
Supplementary Figure 7	Sanger sequencing of isogenic cell lines generated with bystander edits.
Supplementary Figure 8	Sanger sequencing of on-target locus for W131* isogenic cell lines.
Supplementary Figure 9	Next generation sequencing (NGS) of on-target locus for W131* heterozygous isogenic cell lines.
Supplementary Figure 10	Sanger sequencing of on-target locus for L111P isogenic cell lines.
Supplementary Figure 11	NGS of on-target locus for L111P heterozygous isogenic cell lines.
Supplementary Figure 12	Sanger sequencing of on-target locus for D271G isogenic cell lines.
Supplementary Figure 13	NGS of on-target locus for D271G heterozygous isogenic cell lines.
Supplementary Figure 14	Sanger sequencing of on-target locus for L296L isogenic cell lines.
Supplementary Figure 15	NGS of on-target locus for L296L heterozygous isogenic cell lines.
Supplementary Figure 16	NGS of off-target loci for all W131* isogenic cell lines.
Supplementary Figure 17	NGS of off-target loci for all L111P isogenic cell lines.
Supplementary Figure 18	NGS of off-target loci for all D271G isogenic cell lines.
Supplementary Figure 19	NGS of off-target loci for all L296L isogenic cell lines.
Supplementary Figure 20	Quantification of MUTYH protein expression levels in all isogenic cell lines.
Supplementary Figure 21	Western blotting of MUTYH and total protein expression levels of D271G and W131* isogenic cell lines.
Supplementary Figure 22	Western blotting of MUTYH and total protein expression levels of L296L and L111P isogenic cell lines.
Supplementary Figure 23	NGS and western blot analysis of *MUTYH* deletion (*MUTYH*Δ) homozygous isogenic cell lines
Supplementary Figure 24	Schematic and representative agarose gel images of generation of fluorescent reporter plasmids to measure 8-oxoG•A repair.
Supplementary Figure 25	Fluorescence microscopy imaging and flow cytometry plots of positive and negative control intact constructs for the 8-oxoG•A repair reporter in wild-type HEK293T cells.
Supplementary Figure 26	Fluorescence microscopy imaging and flow cytometry plots of the 8-oxoG•A and 8-oxoG•[O] repair reporters, as well as positive and negative controls after insert ligation into the pCAV035 backbone in HEK293T cells.
Supplementary Figure 27	Flow cytometry plots of the 8-oxoG•C, 8-oxoG•A, and 8-oxoG•[O] reporters in *MUTYH*Δ cells
Supplementary Figure 28	Quantification of 8-oxoG•C repair in all isogenic cell lines.
Supplementary Figure 29	Quantification of 8-oxoG•A repair in all isogenic cell lines.
Supplementary Figure 30	Co-immunoprecipitation followed by western blot experiments to probe MUTYH-APE1 interactions in homozygous mutant MUTYH cell lines.
Supplementary Table 1	List of primers used for PCR amplification to produce gRNA plasmids
Supplementary Table 2	List of primers used for Sanger sequencing and Next-generation sequencing (NGS) for *MUTYH* genomic DNA

Supplementary Table 3	List of oligos used to ligate into the fluorescent reporter backbone (pCAV035)
Supplementary Table 4	Off-target loci for the *MUTYH* variants featured in this study.
Supplementary Table 5	List of primers used for NGS of off-target loci
Supplementary Sequences	Sequences of full-length MUTYH (numbering system used in this work) and mammalian reporters used for measuring DNA repair

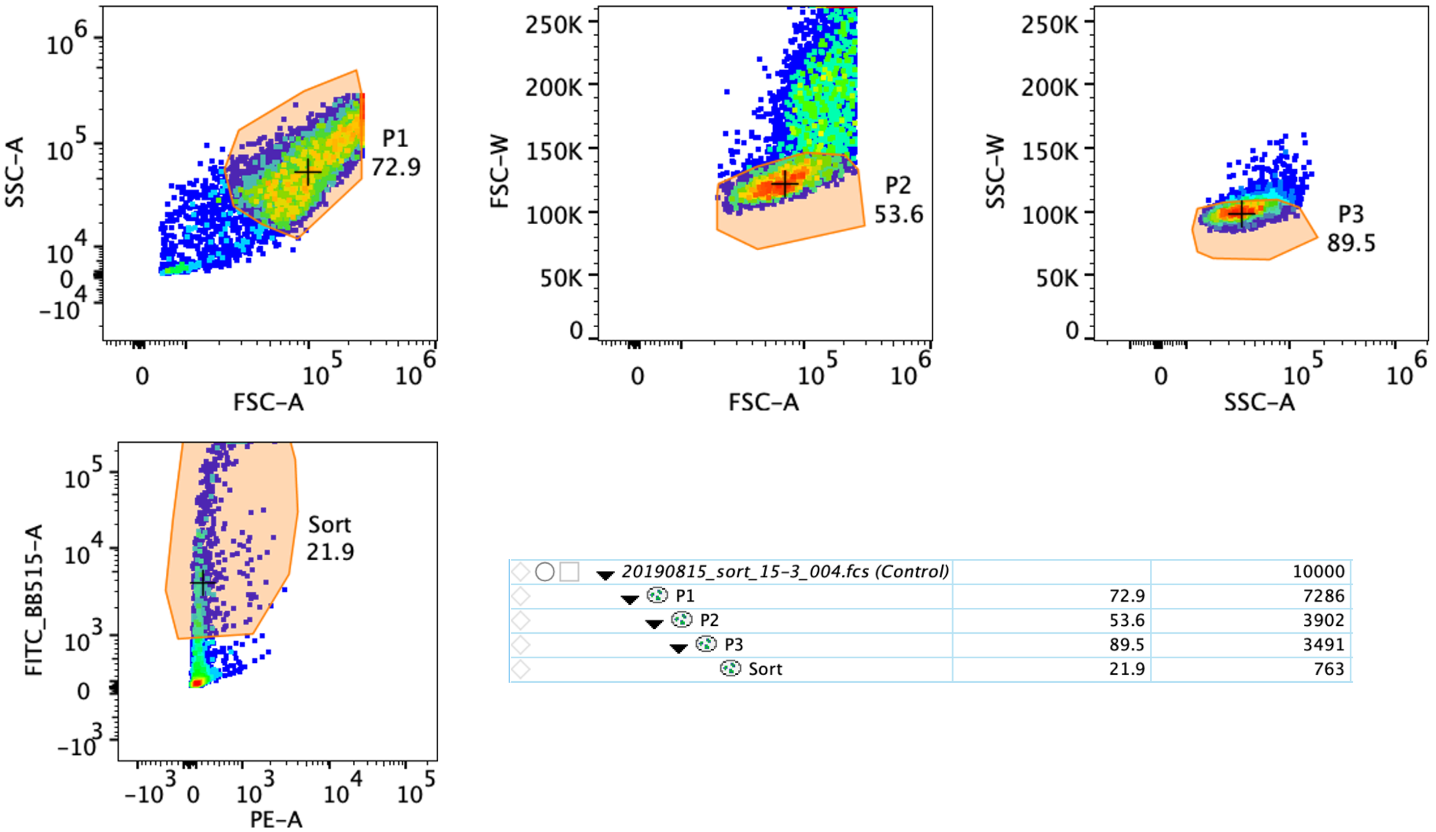

Supplementary Figure 1. Example scatter and sort gates used to generate isogenic cells. Shown are the gating schemes applied for sorting transfected cells based on EGFP fluorescence. Each sample was first gated to remove cell debris using forward scattering area (FSC-A) against side scattering area (SSC-A, P1 gate), then twice gated to remove doublets using forward or side scattering width (FSC-W or SSC-W, respectively) against FSC-A and SSC-A (P2 and P3 gates, respectively). Finally, cells were sorted for EGFP signal (bottom graph, y-axis, Sort gate). “+”’s in the quadrants indicate the median EGFP fluorescence intensity of EGFP-positive cells.

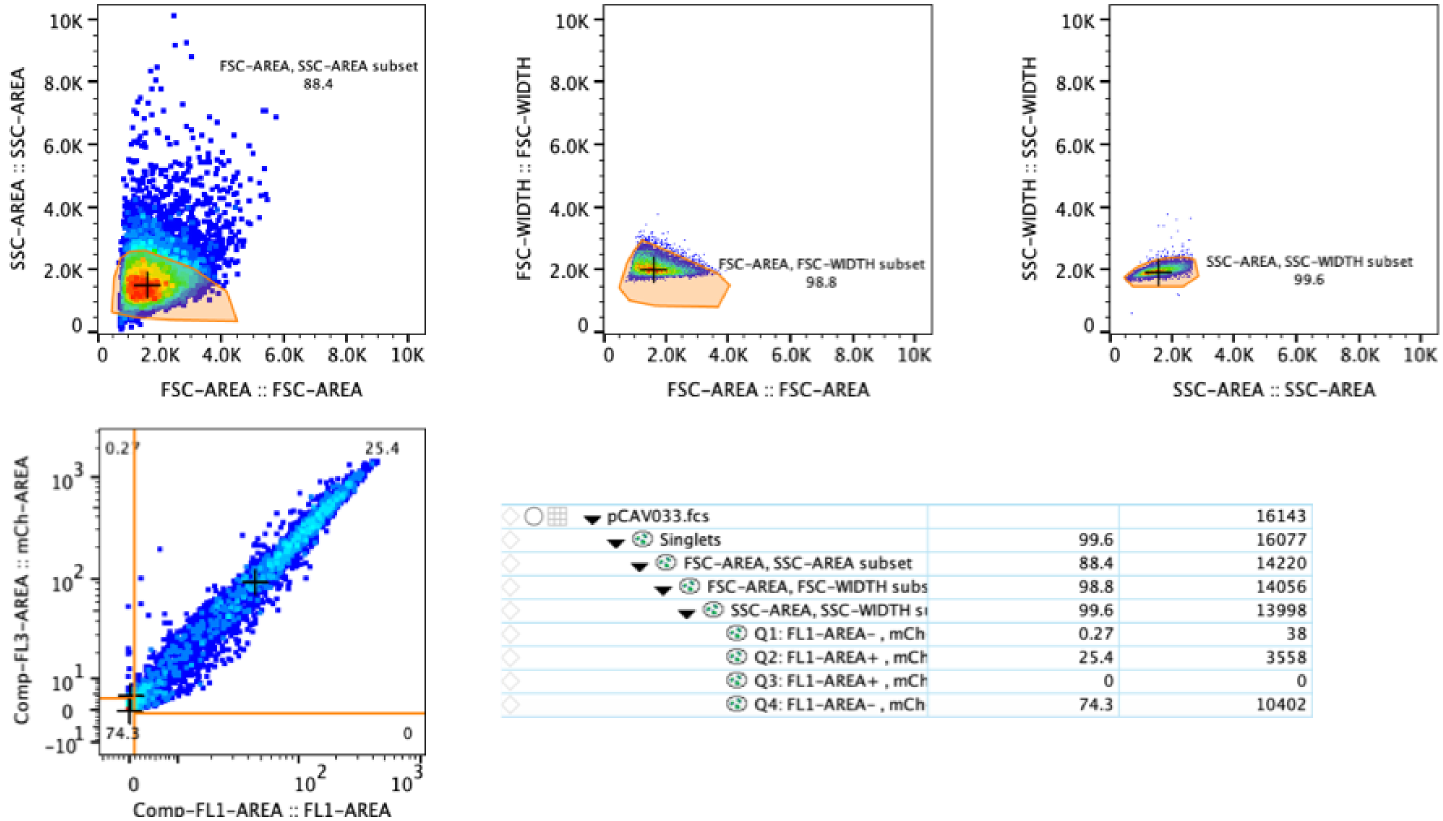

Supplementary Figure 2. Example gating schemes used for DNA repair fluorescent reporters. (Top, left to right) Cell debris was removed using forward scattering area (FSC-A) against side scattering area (SSC-A), and doublets were removed using width (FSC-W or SSC-W) against forward and side scattering area. (Bottom left) Next, EGFP (x-axis) and mCherry (y-axis) fluorescent intensities were used to gate for mCherry+/EGFP- and mCherry+/EGFP+ populations. Fluorescence gates were set based off of cells transfected with mCherry only or EGFP only plasmids. “+”’s in the quadrants indicate the median EGFP fluorescence intensity of EGFP-positive cells.

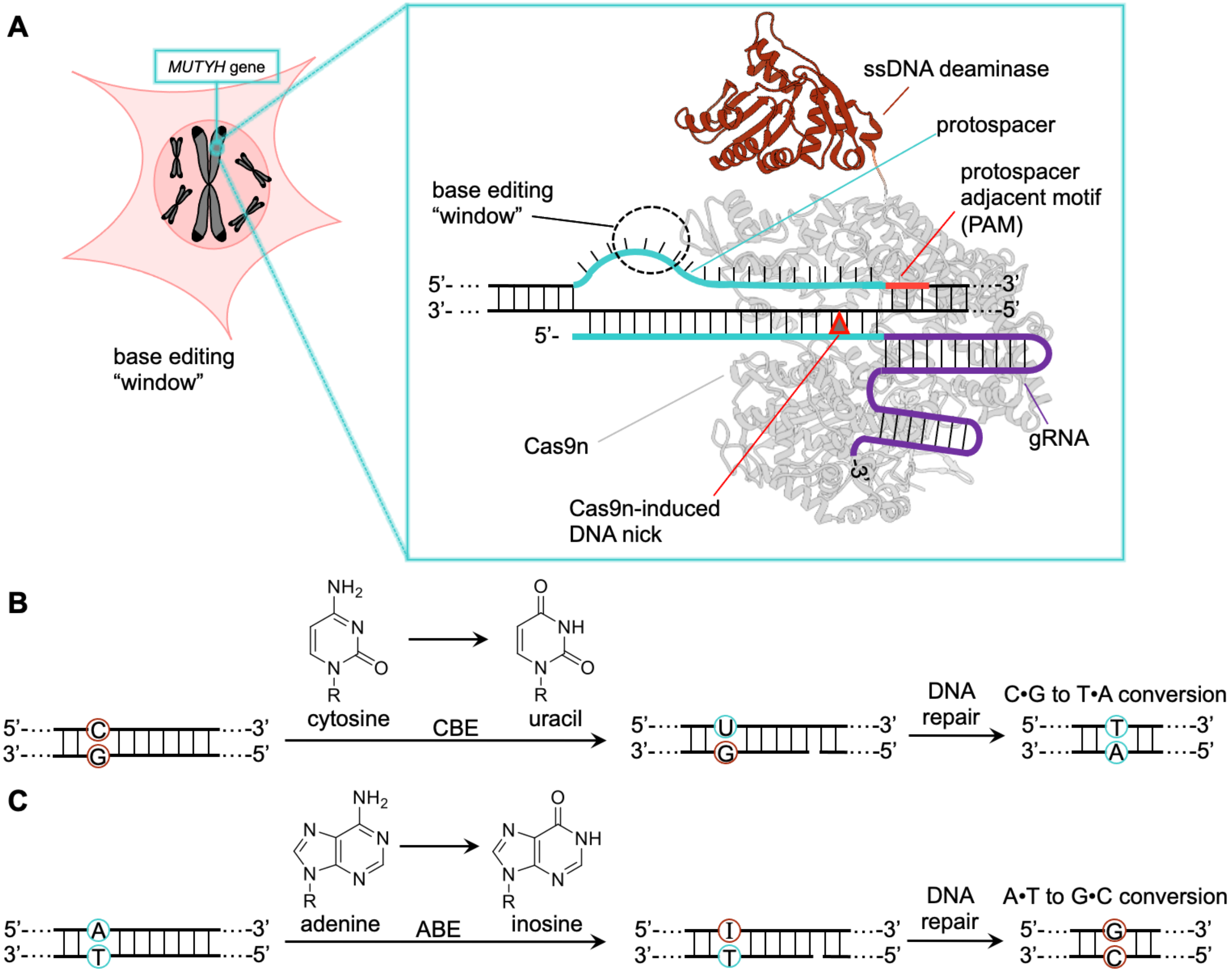

Supplementary Figure 3. Overview of base editing. (A) Base editors are comprised of a catalytically impaired Cas9 protein (Cas9n, grey) fused to a single-stranded DNA (ssDNA) specific deaminase enzyme (orange). The Cas9n protein complexes with a user-defined guide RNA (gRNA), which consists of a scaffold region (purple) that the Cas9n protein recognizes, and a custom spacer sequence (cyan). Binding of Cas9n to the genomic loci of interest is facilitated via base-pairing between the spacer region of the gRNA and the genomic sequence. The region of the genome that matches the spacer sequence is called the protospacer (cyan), and this must also be next to a protospacer adjacent motif (PAM, red), which for the most commonly used Cas9n (from *Streptococcus pyogenes*) is NGG. Cas9n:gRNA binding to the genomic DNA generates an R-loop, which exposes a subset of the protospacer bases to the ssDNA deaminase enzyme (the base editing "window"). While the ssDNA deaminase enzyme modifies bases on the protospacer (B and C), the Cas9n introduces a DNA backbone nick on the DNA strand that is base-paired with the gRNA. (B) The cytosine base editor (CBE) utilizes a cytidine deaminase enzyme to convert cytosines within the base editing "window" into uracils. The Cas9n-induced nick on the G-containing strand promotes replacement of the strand, resulting in an overall C･G to T･A base-pair conversion. (C) The adenine base editor (ABE) utilizes an adenosine deaminase enzyme to convert adenines within the base editing "window" into inosines. The Cas9n-induced nick on the T-containing strand promotes replacement of the strand, resulting in an overall A･T to G･C base-pair conversion. Bystander editing occurs when additional Cs or As are present in the base editing "window" in addition to the target, desired base.

Identifier	MUTYH mutant	Pathogenicity Interpretations	BE	Protospacer Sequence	Editing on Target Base?	Editing on Bystander Base?	Isogenic Cell Lines Generated?
1	W12*	pathogenic (2)	BE4	GTACCCACAGACGACTCAGG	Yes	Yes	Yes; WT(1), Homo (2), Het (0)
2	P18L	benign likely (3), benign (9), VUS (4)	BE4	AGGAAGCCACGAGCAGCCGT	Yes (low)	Yes	No
**3**	**L111P**	likely pathogenic (2)	ABE7.10	ATGGTAGGTCCCGTTTCTCT	Yes	No	Yes; WT(3), Homo (3), Het (3)
4	Y128H	VUS (1)	ABE7.10	CAGCATATGCCCGCCTGTCC	Yes (low)	N/A	No
**5**	**W131***	pathogenic (4) & likely pathogenic (2)	BE4	GAGACCCACACTGGGGGAAA	Yes	Yes	Yes; WT(3), Homo (3), Het (3)
6	Y179C	pathogenic (52), likely pathogenic (2)	ABE7.10	GGGCTACTATTCTCGTGGCC	No	N/A	No
7	R182C	benign (1), pathogenic (10), likely pathogenic (3)	BE4	TTCTCGTGGCCGGCGGCTGC	Yes (low)	Yes	No
8	G189E	VUS (1)	BE4	GAGCTCCCTCCTGCAGCCGC	No	N/A	No
9	I223V	VUS (25)	ABE7.10	GCCATTGCCTCTATCGCCTT	No	N/A	No
10	R241W	pathogenic (11) & likely pathogenic (4)	BE4	CACGGGTGCTGTGCCGTGTC	No	N/A	No
11	R245C	pathogenic (11) & likely pathogenic (1)	BE4	TGCCGTGTCCGAGCCATTGG	Yes	Yes	Yes; WT(1), Homo (0), Het (1)
12	V246I	VUS (7)	BE4	CGGACACGGCACAGCACCCG	Yes	Yes	Yes; WT(1), Homo (1), Het (1)
13	Q260*	pathogenic (2) & likely pathogenic (1)	BE4	TCCCAGCAGCTCTGGTAGGA	No	N/A	No
**14**	**D271G**	VUS (1)	ABE7.10	GTGGACCCAGCCCGGCCAGG	Yes	No	Yes; WT(3), Homo (3), Het (3)
15	P295L	pathogenic (13) & likely pathogenic (4)	BE4	AGCGCCCACTGTGCAGCCAG	Yes	Yes	Yes; WT(1), Homo (1), Het (1)
**16**	**L296L**	likely benign (7) & VUS (1)	BE4	CCACTGTGCAGCCAGTGCCC	Yes	No	Yes; WT(3), Homo (3), Het (3)
17	E303E	benign likely (2)	BE4	ACAGGCTCTCCACAGGGCAC	Yes	Yes	Yes; WT(1), Homo (2), Het (1)
18	S304N	VUS (5)	BE4	ACAGGCTCTCCACAGGGCAC	Yes	Yes	Yes; WT(1), Homo (1), Het (1)

Supplementary Figure 4. List of MUTYH mutants evaluated for genome editing. Listed are the protein mutation (using the MUTYH isoform 5 numbering system), the clinical classification(s) from the ClinVar database, which base editor (BE) was used to install each variant, and the protospacer sequence. The target base within each protospacer is indicated in purple, and bystander bases are indicated in orange. Also listed are whether or not editing of the target base was detected in bulk editing experiments, if bystander editing was also observed, and whether or not isogenic cell lines were generated. The ten grey rows were those in which high enough editing of the target base was observed in bulk experiments to attempt generation of isogenic cell lines. The four mutants that are bolded were those in which three each of null, heterozygous, and homozygous isogenic cell lines were generated.

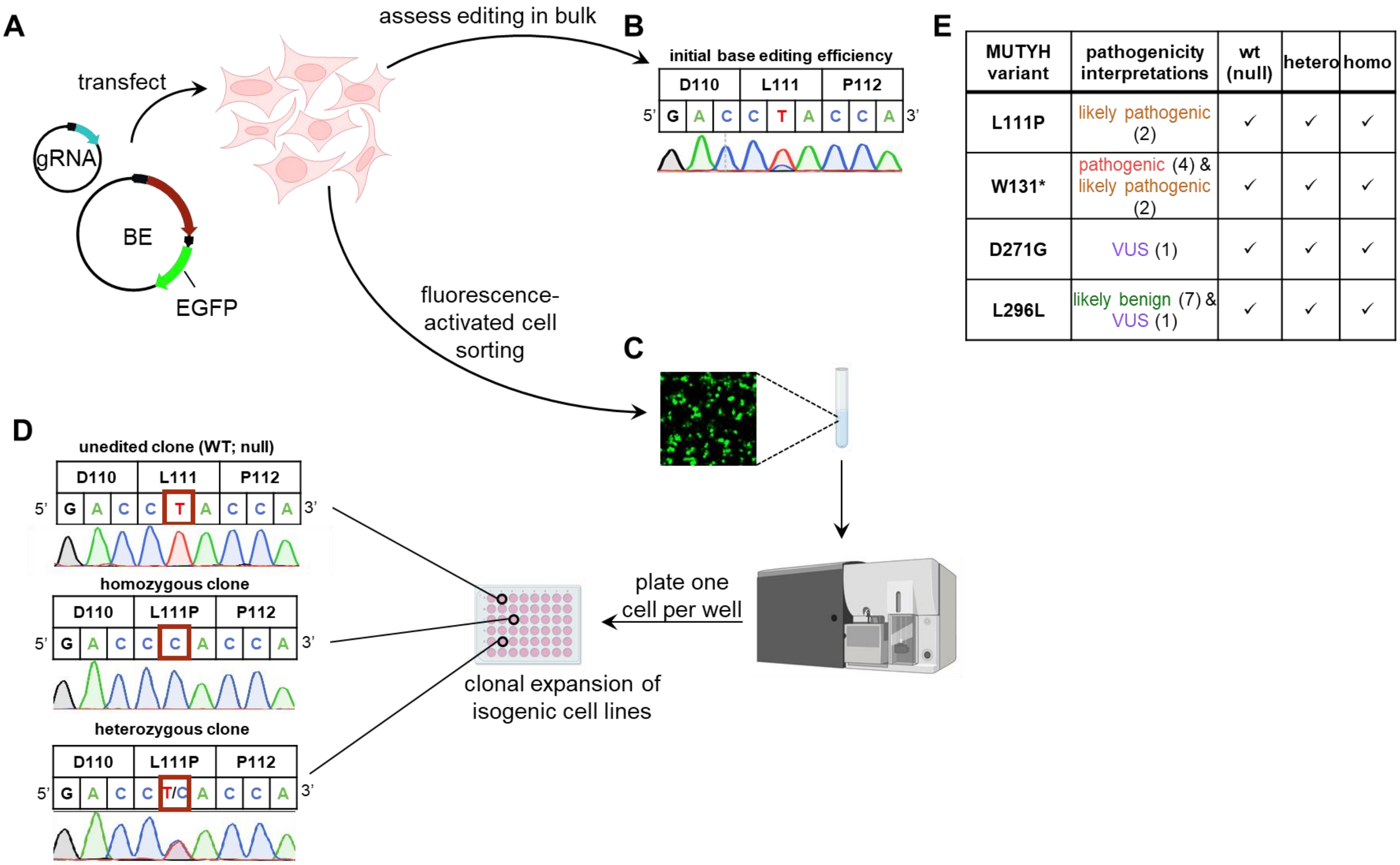

Supplementary Figure 5. Schematic of experimental work-flow for evaluating BE:gRNA combinations and generating isogenic cell lines. (A) Plasmids encoding gRNAs targeted to the protospacers indicated in Supplementary Figure 4 and appropriate base editor (BE) were transfected into HEK293T cells. The BE plasmid also encodes for an EGFP fluorescent marker, which is used in part (C) for isogenic cell line generation. (B) Three days post-transfection, genomic DNA was harvested and genomic loci of interest were amplified via PCR and sequenced with Sanger sequencing to evaluate bulk editing efficiencies. Shown is the Sanger sequencing trace from the L111P sample. (C) For BE:gRNA combinations with efficient (estimated at >20%) editing efficiencies as evaluated in bulk, step A was repeated. Fluorescence activated cell sorting (FACS) was then used to sort single, EGFP-positive cells into individual wells of a 96-well plate. Gating strategies used to isolate single cells with FACS are shown in Supplementary Figure 1. (D) Cells were allowed to clonally expand for 1–2 weeks. A subset of the cells from each colony was harvested and the loci of interest were again sequenced to genotype the cell line. Shown are representative data from each genotype (null, heterozygous, and homozygous) for the L111P mutant. The target nucleotide is highlighted in red. For sequencing data of all nine L111P lines, see Supplementary Figure 8. (E) For each of the four mutations listed, three each of wild-type (null), heterozygous, and homozygous cell lines were generated.

Bulk editing efficiencies – No or low editing of target base			
Identifier	MUTYH mutant	Protospacer Sequence	Sanger Trace
2	P18L	AGGAAGCCACGAGCAGCCG	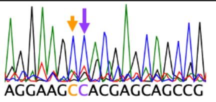
4	Y128H	CAGCATATGCCCGCCTGTCC	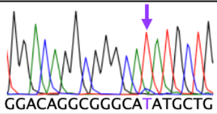
6	Y179C	GGGCTACTATTCTCGTGGCC	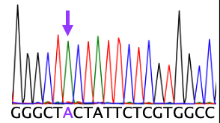
7	R182C	TTCTCGTGGCCGGCGGCTGC	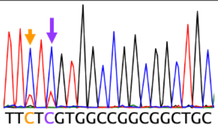
8	G189E	GAGCTCCCTCCTGCAGCCGC	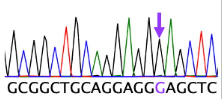
9	I223V	GCCATTGCCTCTATCGCCTT	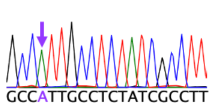
10	R241W	CACGGGTGCTGTGCCGTGTC	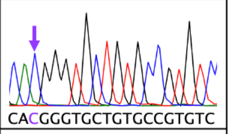
13	Q260*	TCCCAGCAGCTCTGGTAGGA	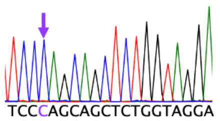

Supplementary Figure 6. Sanger sequencing of bulk editing efficiencies of BE:gRNA combinations with no or low editing of the target base. Cells were treated as shown in Supplementary Figure 5B. Listed are the protein mutation (using the MUTYH isoform 5 numbering system) and protospacer sequence for the MUTYH mutations from Supplementary Figure 4 in which no or low editing of the target base was detected in bulk editing samples. The target base within each protospacer is indicated in purple, and bystander bases are indicated in orange. Also shown are Sanger sequencing traces of the bulk editing experiment, with purple arrows pointing to the target base and orange arrows pointed at bystander bases. The identifying numbers in the left most column corresponds to the identifying numbers in Supplementary Figure 4.

Isogenic cell lines generated with bystander edits				
Identifier	MUTYH mutant	Protospacer Sequence	Isogenic Line #1	Isogenic Line #2
1	W12*	GTACCCACAGACGACTCAGG	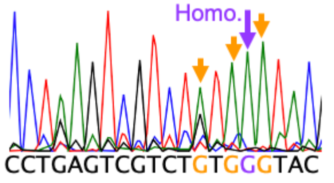	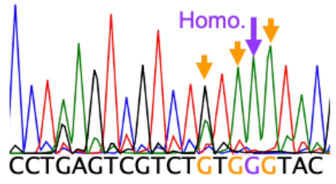
11	R245C	TGCCGTGTCCGAGCCATTGG	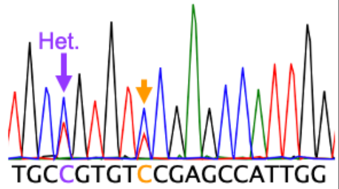	
12	V246I	CGGACACGGCACAGCACCCG	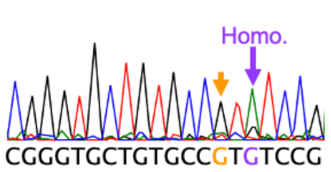	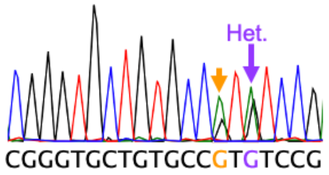
15	P295L	AGCGCCCACTGTGCAGCCAG	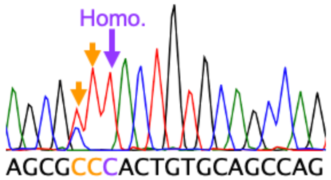	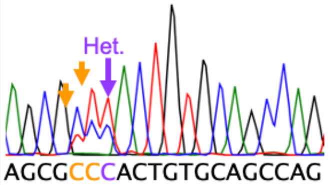
17	E303E	ACAGGCTCTCCACAGGGCAC	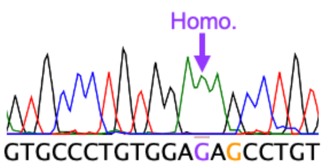	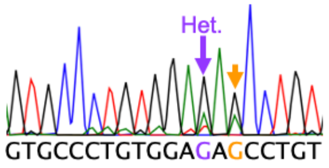
18	S304N	ACAGGCTCTCCACAGGGCAC	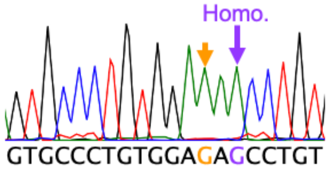	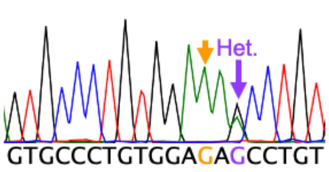

Supplementary Figure 7. Sanger sequencing of isogenic cell lines generated with bystander edits. Cells were treated as shown in Supplementary Figure 5C. Listed are the protein mutation (using the MUTYH isoform 5 numbering system) and protospacer sequence for the MUTYH mutations from Supplementary Figure 4 in which isogenic cell lines were generated with bystander mutations. The target base within each protospacer is indicated in purple, and bystander bases are indicated in orange. Also shown are Sanger sequencing traces of the isogenic cell lines generated, with purple arrows pointing to the target base and orange arrows pointed at bystander bases. The genotype of each cell line ("homo." for homozygous and "het." for heterozygous) is indicated above the purple arrow. The identifying numbers in the left most column corresponds to the identifying numbers in Supplementary Figure 4.

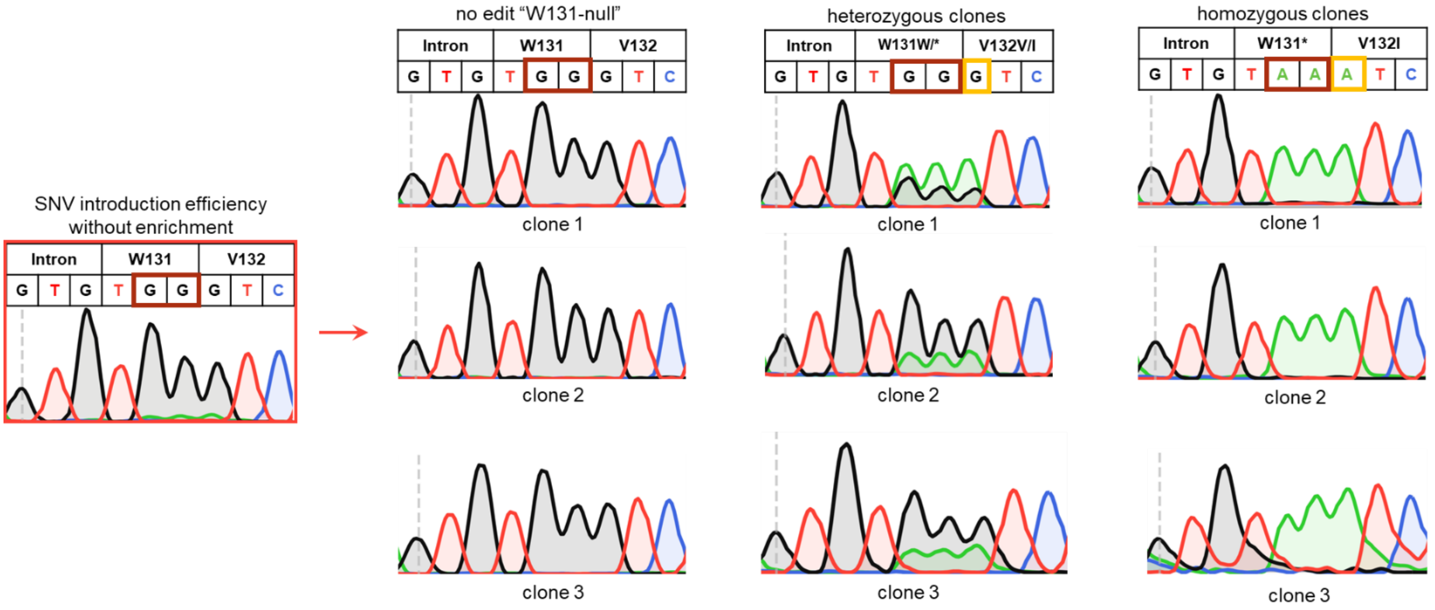

Supplementary Figure 8. Sanger sequencing of on-target locus for W131* isogenic cell lines. Isogenic cell lines harboring the W131* mutation were generated as shown in Supplementary Figure 5C. The *MUTYH* locus surrounding codon 131 was sequenced with Sanger sequencing. Shown are the Sanger sequencing traces, zoomed in on codons 131 and 132 (plus the intronic region before codon 131), of bulk cells prior to FACS (left) and all nine isogenic cell lines (right), labeled with their genotype.

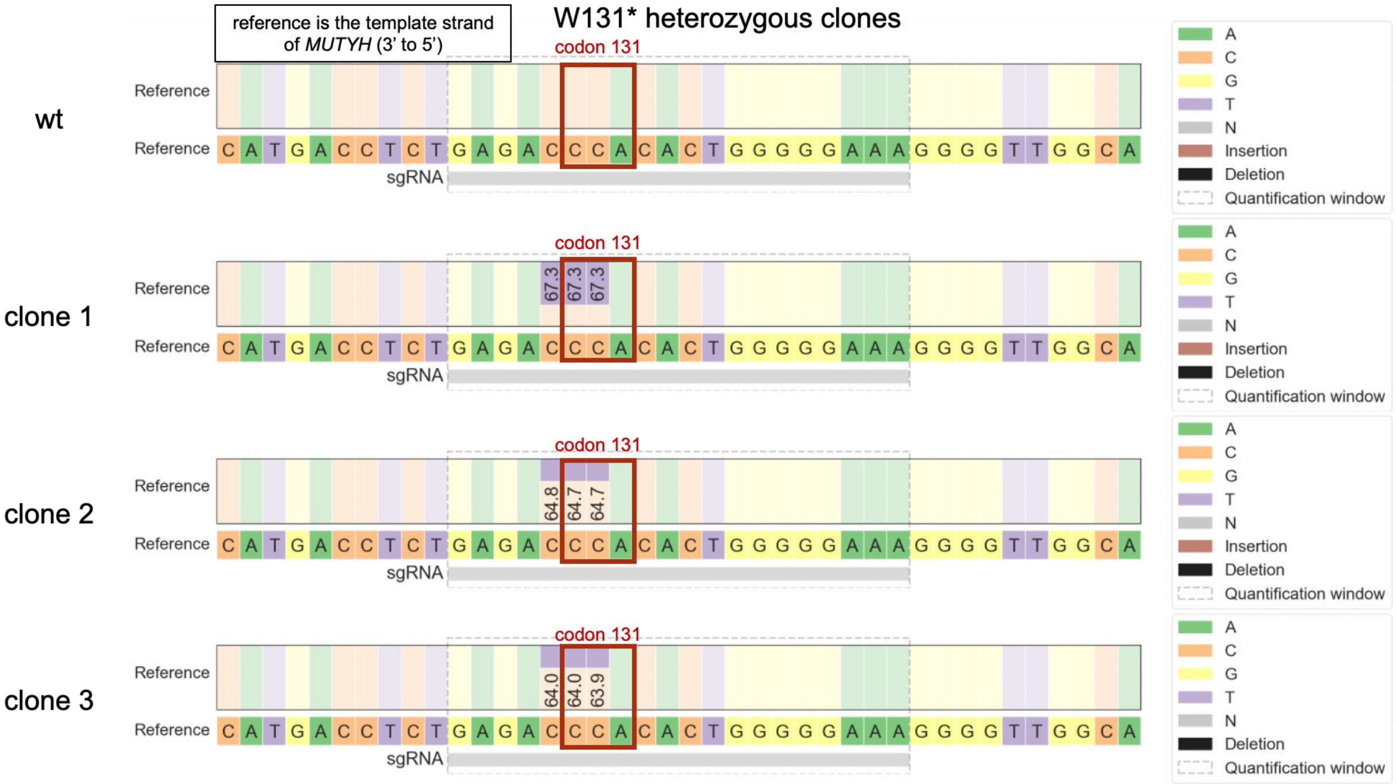

Supplementary Figure 9. Next generation sequencing (NGS) of on-target locus for W131* heterozygous isogenic cell lines. Isogenic cell lines harboring the W131* mutation were generated as shown in Supplementary Figure 5C. The *MUTYH* locus surrounding codon 131 (shown in outlined box) of the heterozygous clones and wild-type HEK293T cells was sequenced with next generation sequencing (NGS). Shown are nucleotide percentage tables of the protospacer (portion within dotted line) and 10 base-pairs on either side. The nucleotide percentage table shows the composition of each base at each position in the amplicon, according to the colors shown on the right. The gRNA sequence shown is in the 5' to 3' orientation, targeting the complimentary template strand of *MUTYH*; reference shown is in the 3' to 5' orientation.

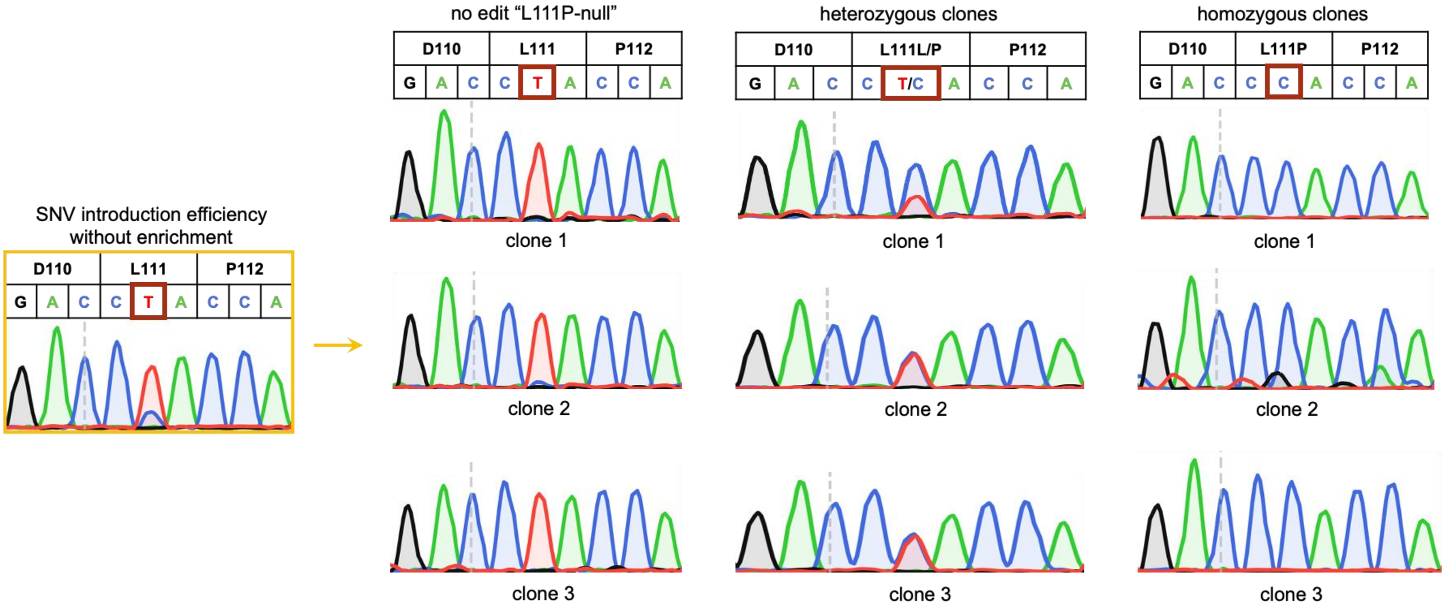

Supplementary Figure 10. Sanger sequencing of on-target locus for L111P isogenic cell lines. Isogenic cell lines harboring the L111P mutation were generated as shown in Supplementary Figure 5C. The *MUTYH* locus surrounding codon 111 was sequenced with Sanger sequencing. Shown are the Sanger sequencing traces, zoomed in on codons 110-112, of bulk cells prior to FACS (left) and all nine isogenic cell lines (right), labeled with their genotype.

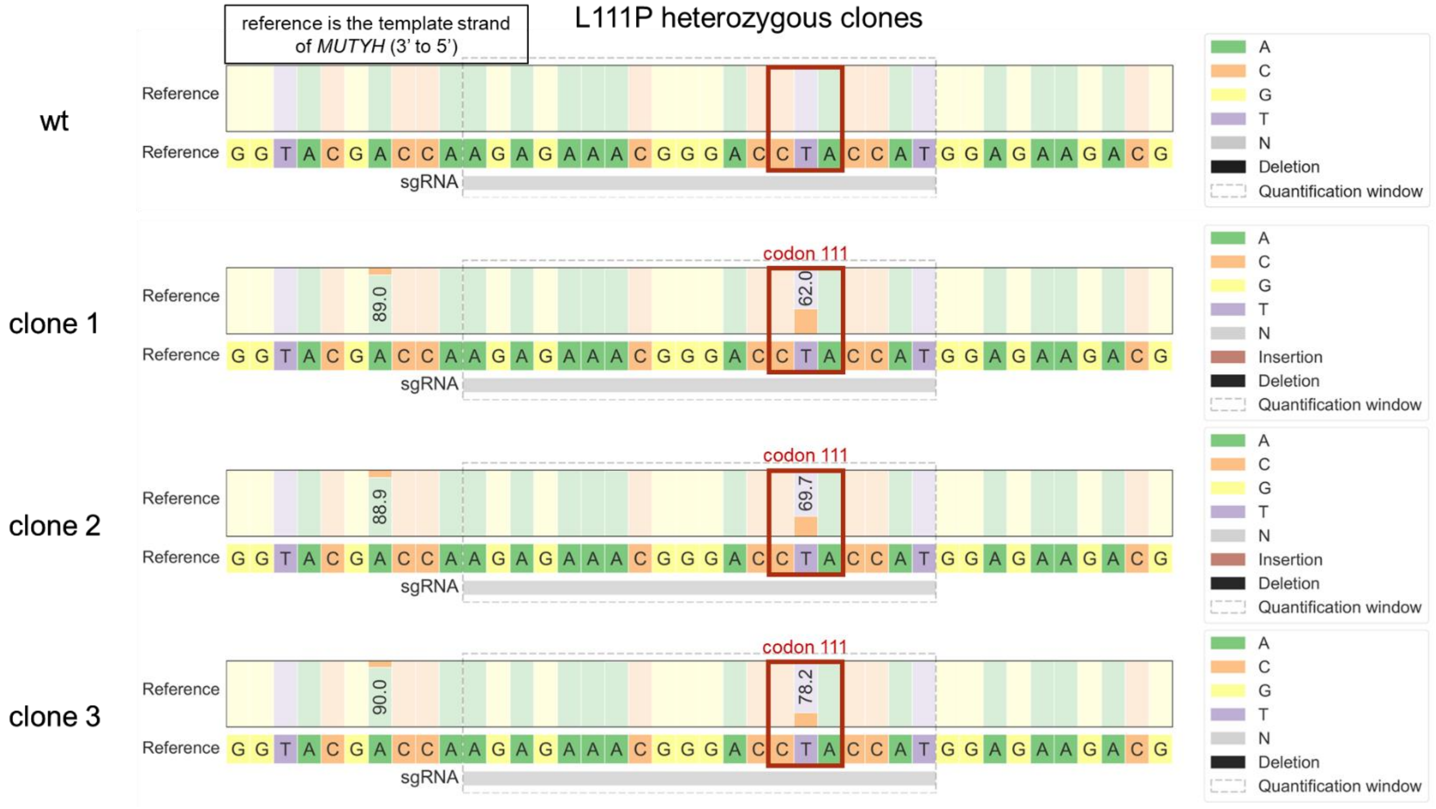

Supplementary Figure 11. Next generation sequencing of on-target locus for L111P heterozygous isogenic cell lines. Isogenic cell lines harboring the L111P mutation were generated as shown in Supplementary Figure 5C. The *MUTYH* locus surrounding codon 111 (shown in outlined box) of the heterozygous clones and wild-type HEK293T cells was sequenced with next generation sequencing (NGS). Shown are nucleotide percentage tables of the protospacer (portion within dotted line) and 10 base-pairs on either side. The nucleotide percentage table shows the composition of each base at each position in the amplicon, according to the colors shown on the right. The gRNA sequence shown is in the 5' to 3' orientation, targeting the complimentary template strand of *MUTYH*; reference shown is in the 3' to 5' orientation.

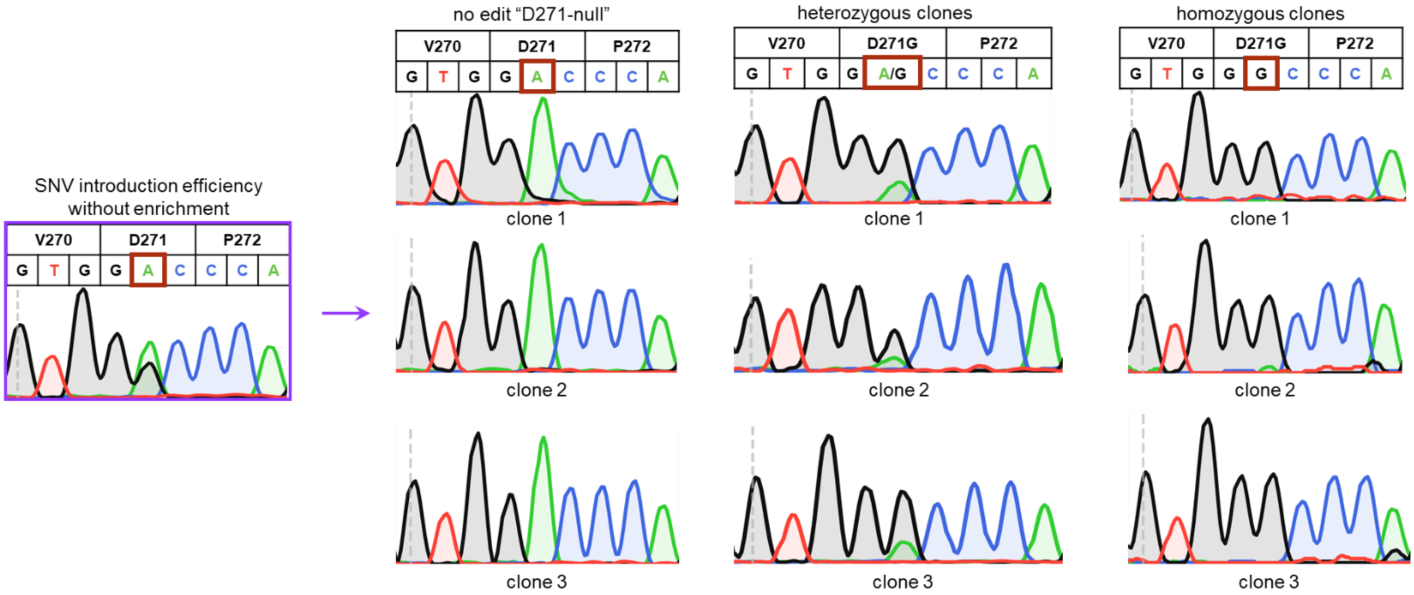

Supplementary Figure 12. Sanger sequencing of on-target locus for D271G isogenic cell lines. Isogenic cell lines harboring the D271G mutation were generated as shown in Supplementary Figure 5C. The *MUTYH* locus surrounding codon 271 was sequenced with Sanger sequencing. Shown are the Sanger sequencing traces, zoomed in on codons 270-272, of bulk cells prior to FACS (left) and all nine isogenic cell lines (right), labeled with their genotype.

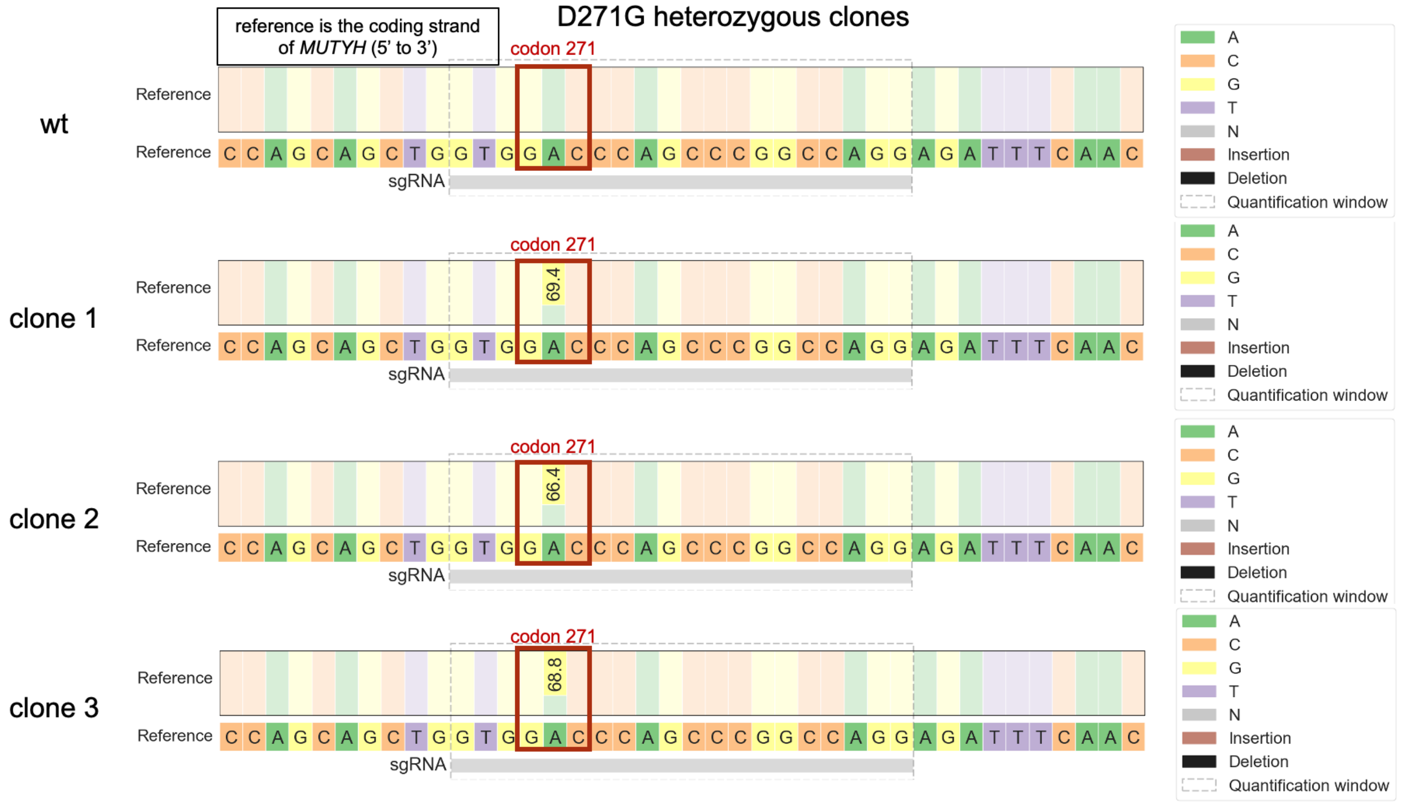

Supplementary Figure 13. NGS of on-target locus for D271G heterozygous isogenic cell lines. Isogenic cell lines harboring the D271G mutation were generated as shown in Supplementary Figure 5C. The *MUTYH* locus surrounding codon 271 (shown in outlined box) of the heterozygous clones and wild-type HEK293T cells was sequenced with NGS. Shown are nucleotide percentage tables of the protospacer (portion within dotted line) and 10 base-pairs on either side. The nucleotide percentage table shows the composition of each base at each position in the amplicon, according to the colors shown on the right. The gRNA sequence shown is in the 5' to 3' orientation, targeting the coding strand of *MUTYH*; reference shown is in the 5' to 3' orientation.

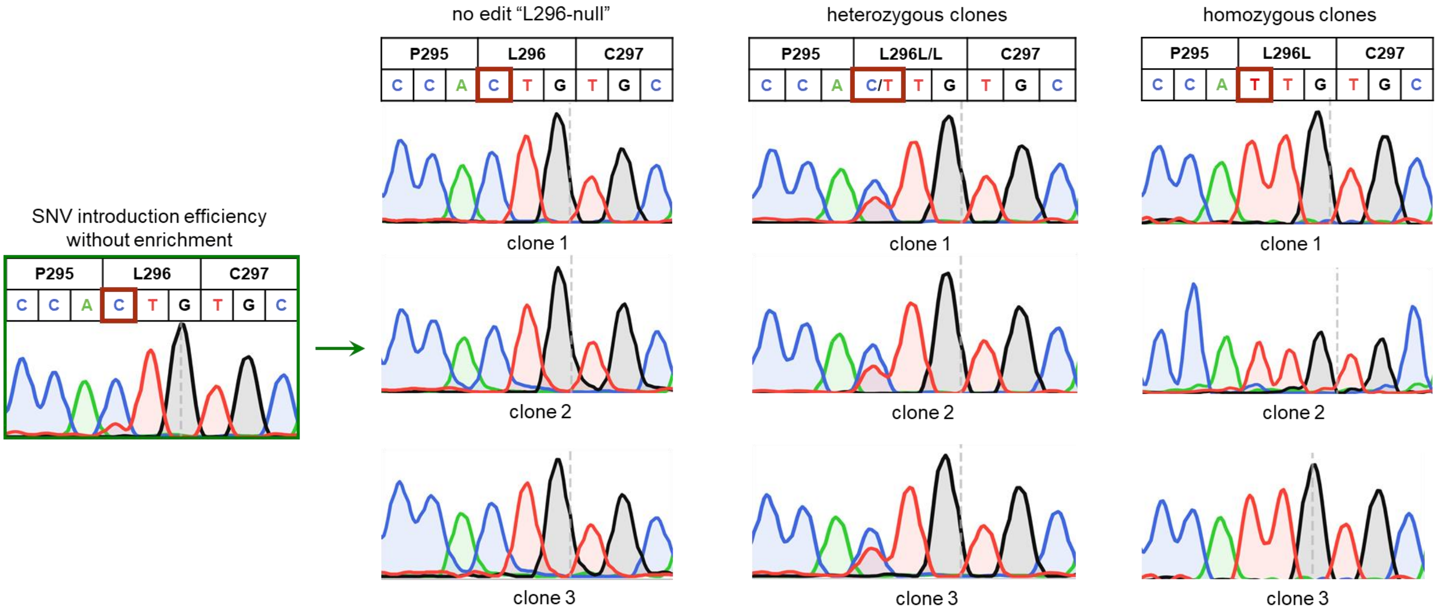

Supplementary Figure 14. Sanger sequencing of on-target locus for L296L isogenic cell lines. Isogenic cell lines harboring the L296L mutation were generated as shown in Supplementary Figure 5C. The *MUTYH* locus surrounding codon 296 was sequenced with Sanger sequencing. Shown are the Sanger sequencing traces, zoomed in on codons 295-297, of bulk cells prior to FACS (left) and all nine isogenic cell lines (right), labeled with their genotype.

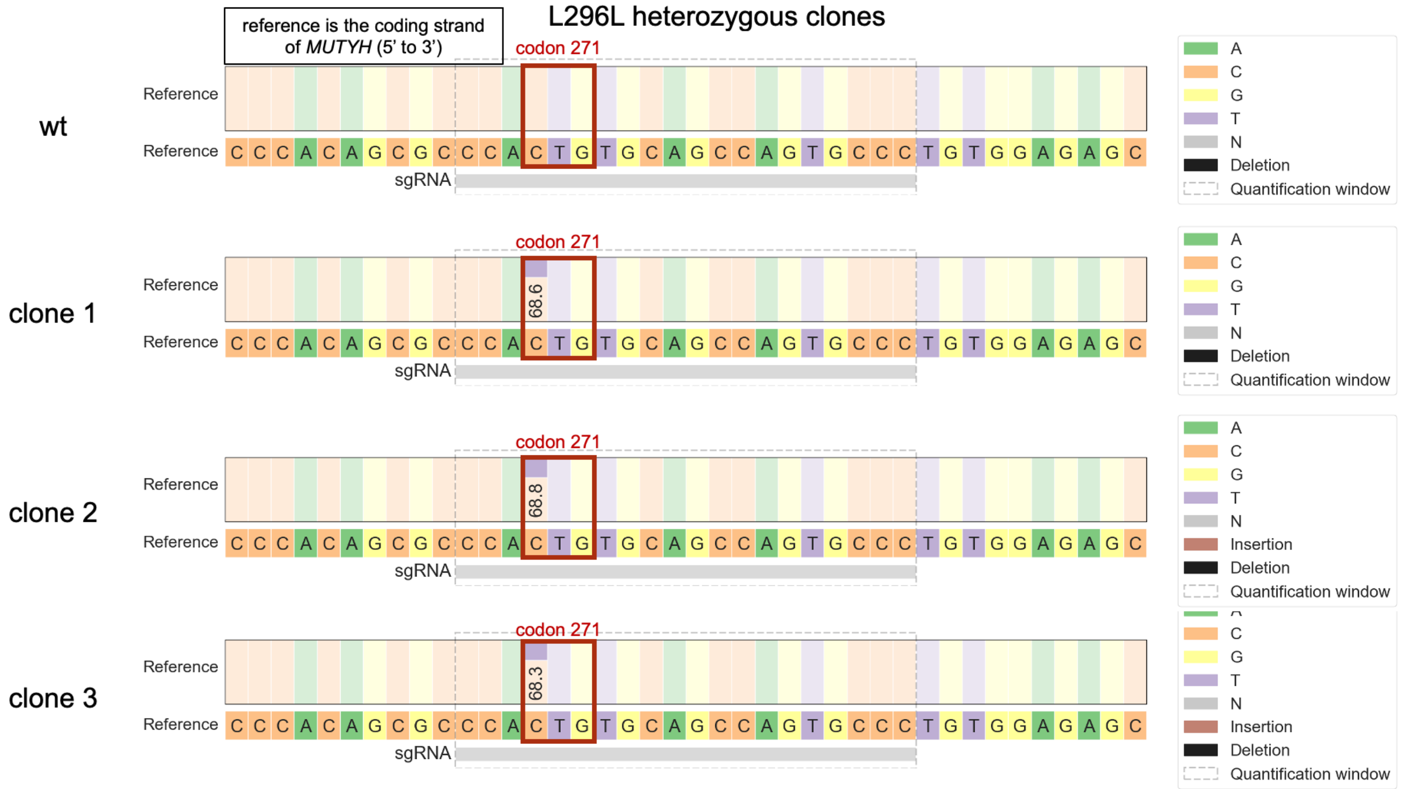

Supplementary Figure 15. NGS of on-target locus for L296L heterozygous isogenic cell lines. Isogenic cell lines harboring the L296L mutation were generated as shown in Supplementary Figure 5C. The *MUTYH* locus surrounding codon 296 (shown in outlined box) of the heterozygous clones and wild-type HEK293T cells was sequenced with NGS. Shown are nucleotide percentage tables of the protospacer (portion within dotted line) and 10 base-pairs on either side. The nucleotide percentage table shows the composition of each base at each position in the amplicon, according to the colors shown on the right. The gRNA sequence shown is in the 5' to 3' orientation, targeting the coding strand of *MUTYH*; reference shown is in the 5' to 3' orientation.

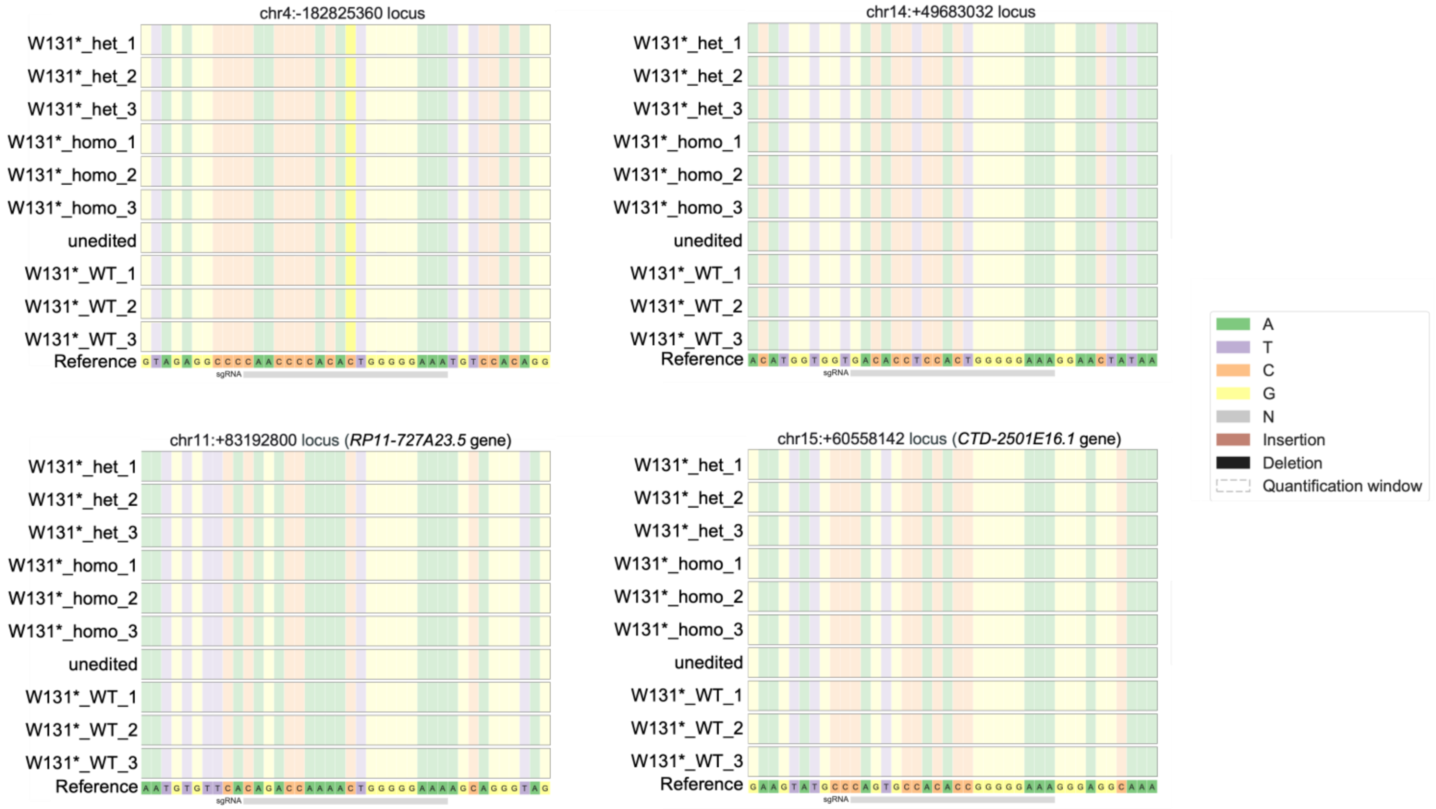

Supplementary Figure 16. NGS of off-target loci for all W131* isogenic cell lines. Isogenic cell lines harboring the W131* mutation were generated as shown in Supplementary Figure 5C. Genomic loci corresponding to the top two coding and two non-coding predicted off-target sites for the W131* protospacer (see Supplementary Table 4) were sequenced for all nine clones plus wild-type HEK293T cells with NGS. Shown are nucleotide percentage tables of the potential off-target protospacers (portion within dotted line) and 10 base-pairs on either side. The nucleotide percentage table shows the composition of each base at each position in the amplicon, according to the colors shown on the left. No editing above wild-type controls were observed at any of the off-target loci.

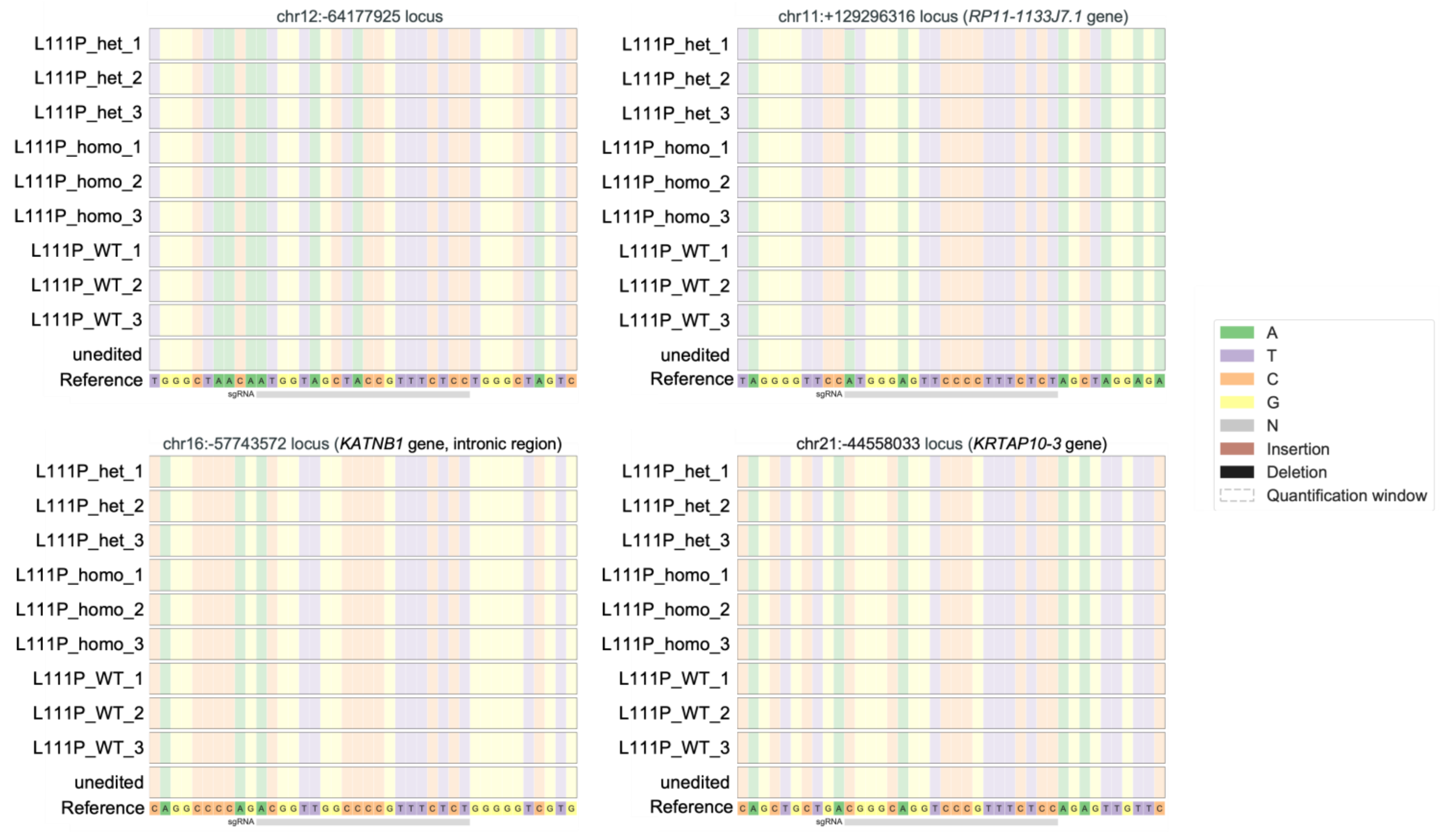

Supplementary Figure 17. NGS of off-target loci for all L111P isogenic cell lines. Isogenic cell lines harboring the L111P mutation were generated as shown in Supplementary Figure 5C. Genomic loci corresponding to the top two coding and two non-coding predicted off-target sites for the L111P protospacer (see Supplementary Table 4) were sequenced for all nine clones plus wild-type HEK293T cells with NGS. Shown are nucleotide percentage tables of the potential off-target protospacers (portion within dotted line) and 10 base-pairs on either side. The nucleotide percentage table shows the composition of each base at each position in the amplicon, according to the colors shown on the left. No editing above wild-type controls were observed at any of the off-target loci.

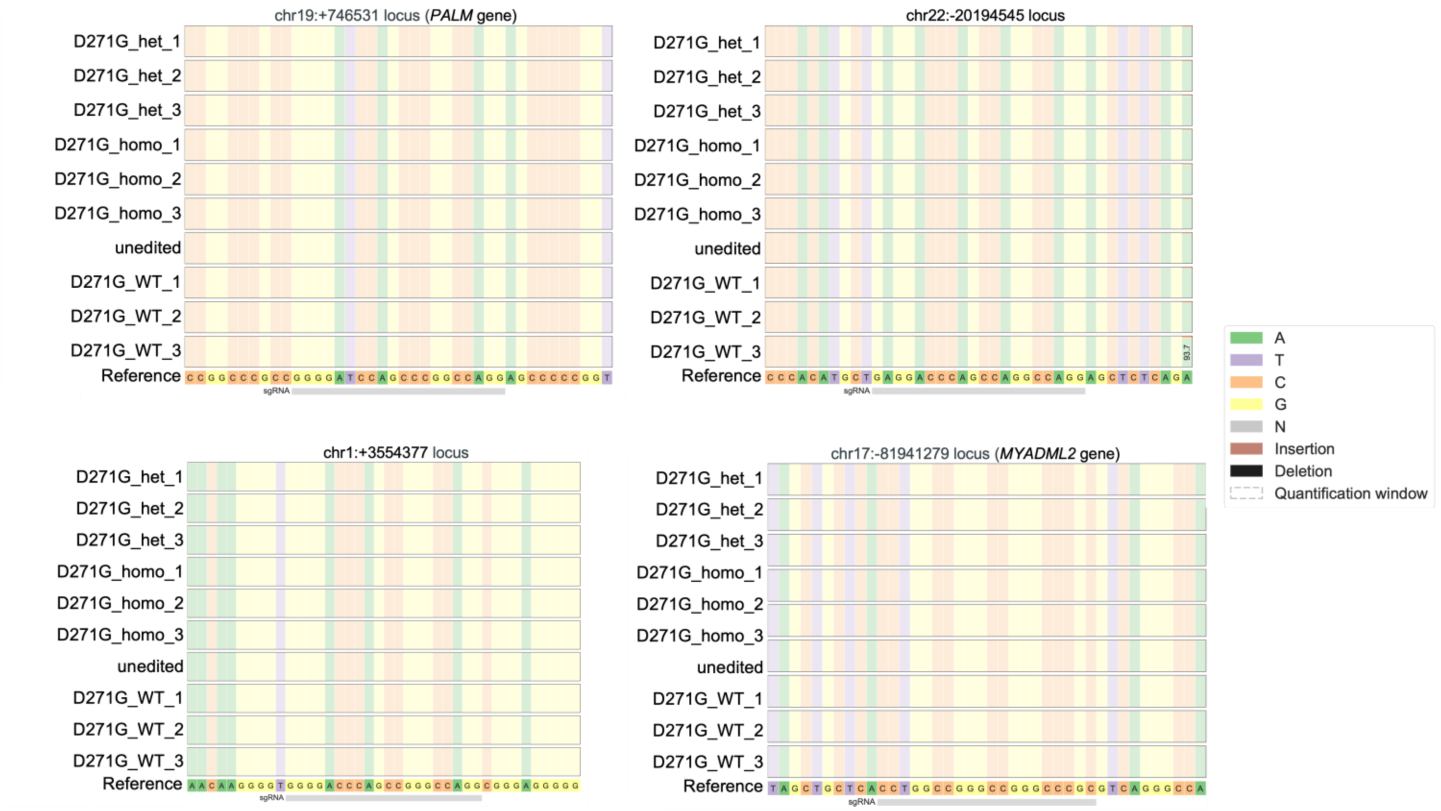

Supplementary Figure 18. NGS of off-target loci for all D271G isogenic cell lines. Isogenic cell lines harboring the D271G mutation were generated as shown in Supplementary Figure 5C. Genomic loci corresponding to the top two coding and two non-coding predicted off-target sites for the D271G protospacer (see Supplementary Table 4) were sequenced for all nine clones plus wild-type HEK293T cells with NGS. Shown are nucleotide percentage tables of the potential off-target protospacers (portion within dotted line) and 10 base-pairs on either side. The nucleotide percentage table shows the composition of each base at each position in the amplicon, according to the colors shown on the left. No editing above wild-type controls were observed at any of the off-target loci.

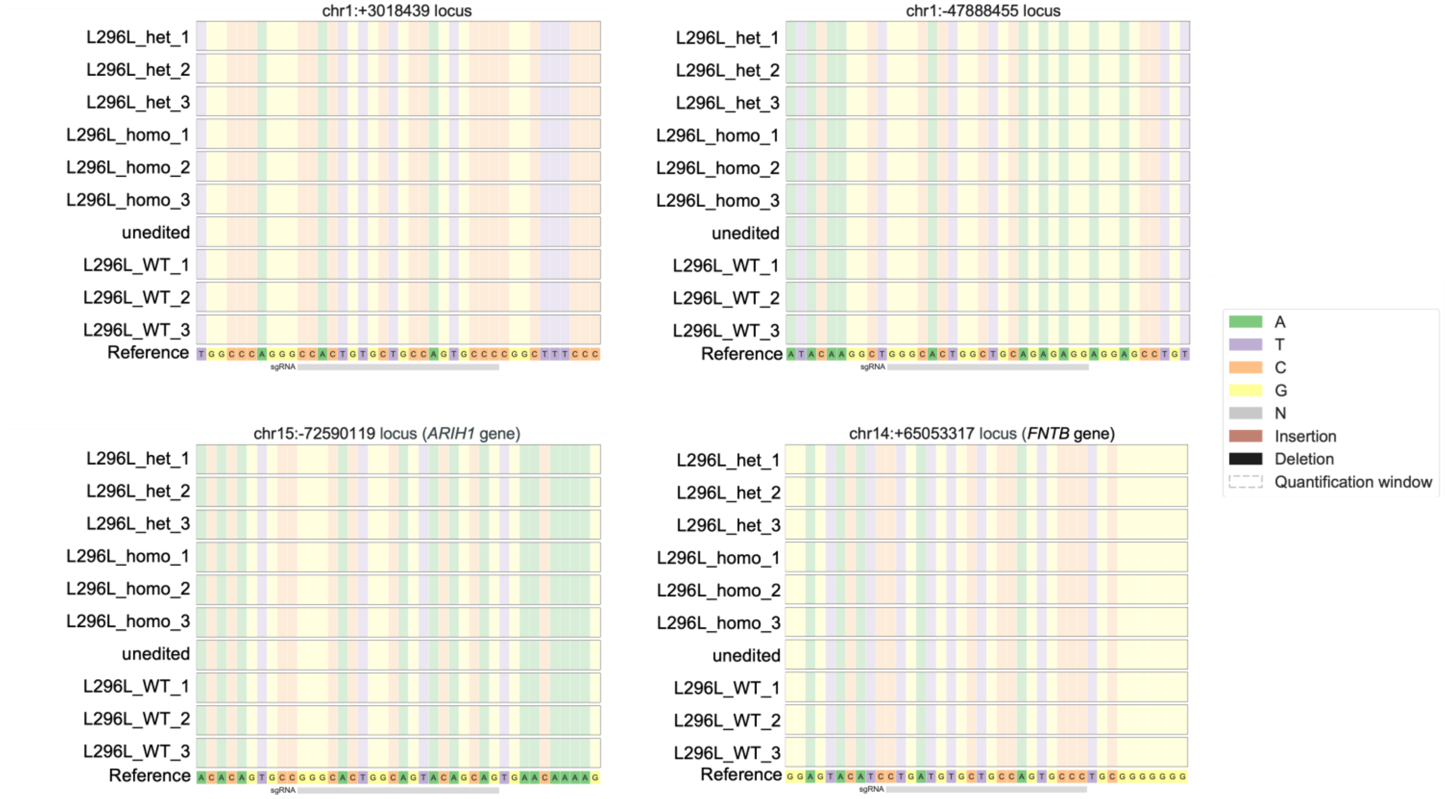

Supplementary Figure 19. NGS of off-target loci for all L296L isogenic cell lines. Isogenic cell lines harboring the L296L mutation were generated as shown in Supplementary Figure 5C. Genomic loci corresponding to the top two coding and two non-coding predicted off-target sites for the L296L protospacer (see Supplementary Table 4) were sequenced for all nine clones plus wild-type HEK293T cells with NGS. Shown are nucleotide percentage tables of the potential off-target protospacers (portion within dotted line) and 10 base-pairs on either side. The nucleotide percentage table shows the composition of each base at each position in the amplicon, according to the colors shown on the left. No editing above wild-type controls were observed at any of the off-target loci.

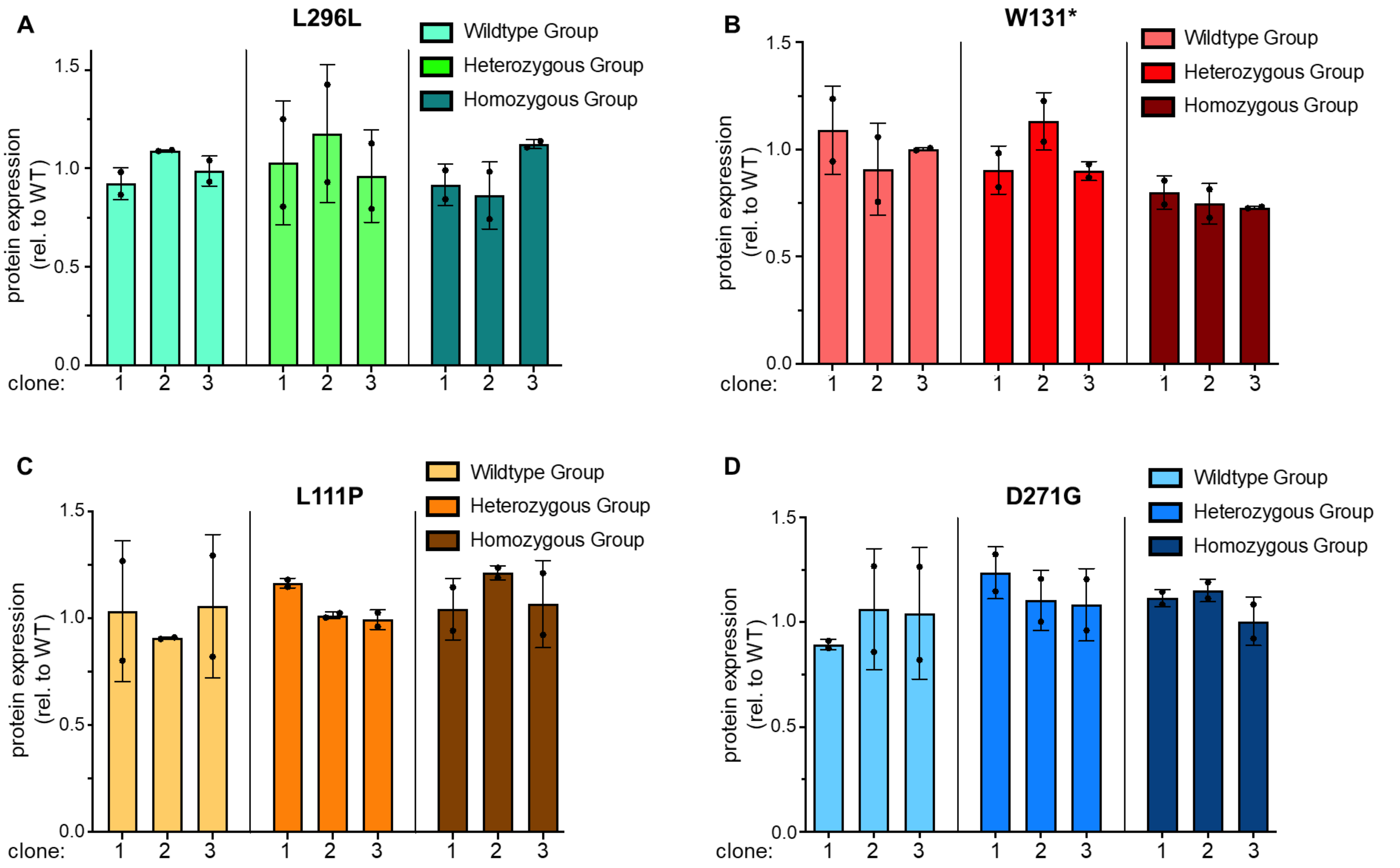

Supplementary Figure 20. Quantification of MUTYH protein expression levels in all isogenic cell lines. All 36 isogenic cell lines were lysed and analyzed for MUTYH protein expression levels by Western blot (see Supplementary Figures 20-21 for Western blots). Intensities of MUTYH protein bands were divided by that of total protein content. These values were then normalized to the average value of the three wild-type (null) cell lines for each variant. Values represent the average of two technical replicates for each isogenic cell line, with each replicate marked individually. Shown are normalized protein levels for the L296L (A), W131* (B), L111P (C), and D271G (D) clones. Within each panel (A-D), the first three clones are wild-type (null), the middle three clones are heterozygous, and the last three clones are homozygous. The color scheme ranges from lighter to darker shade, as indicated in the figure legends.

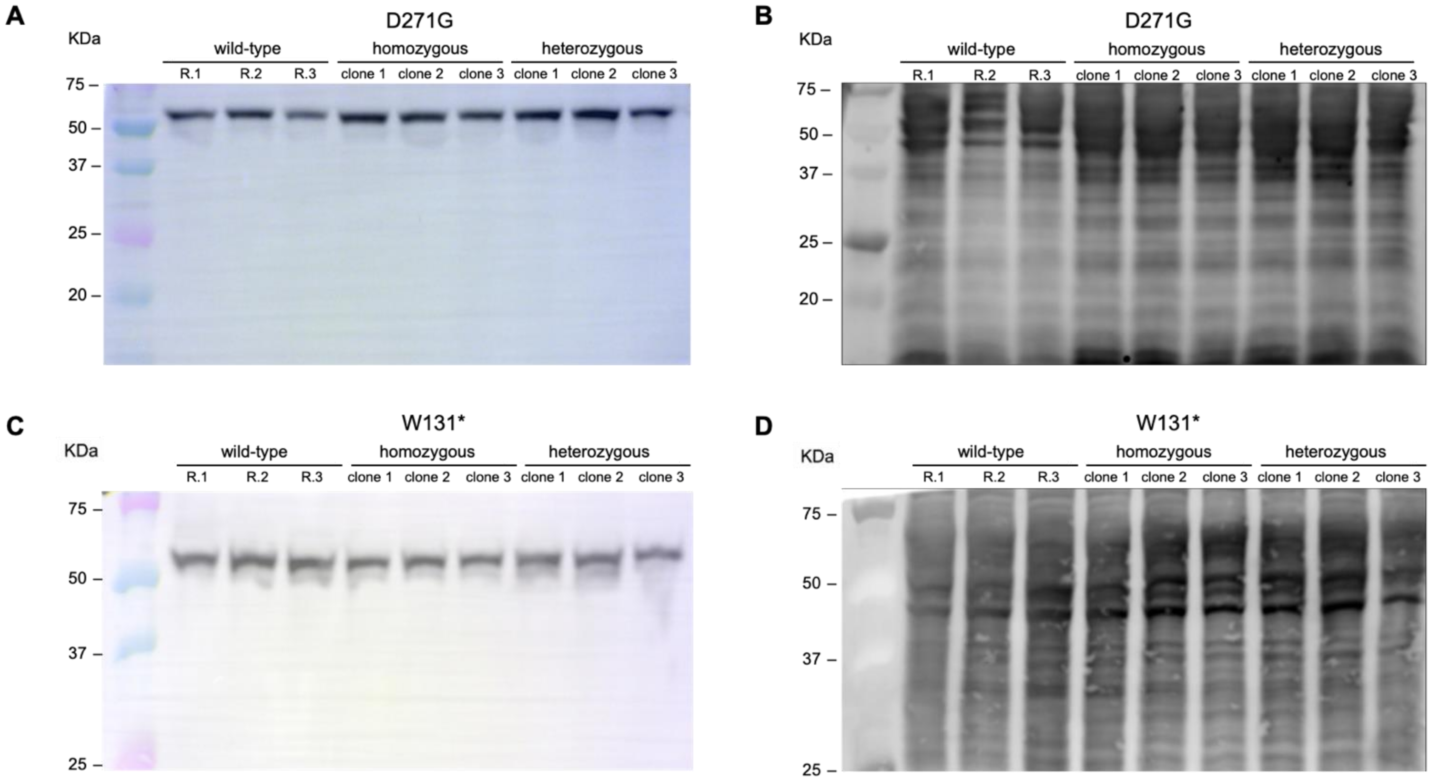

Supplementary Figure 21. Western blotting of MUTYH and total protein expression levels of D271G and W131* isogenic cell lines. D271G (A-B) and W131* (C-D) isogenic cell lines were lysed and stained for MUTYH (A and C) or total (B and D) protein expression levels.

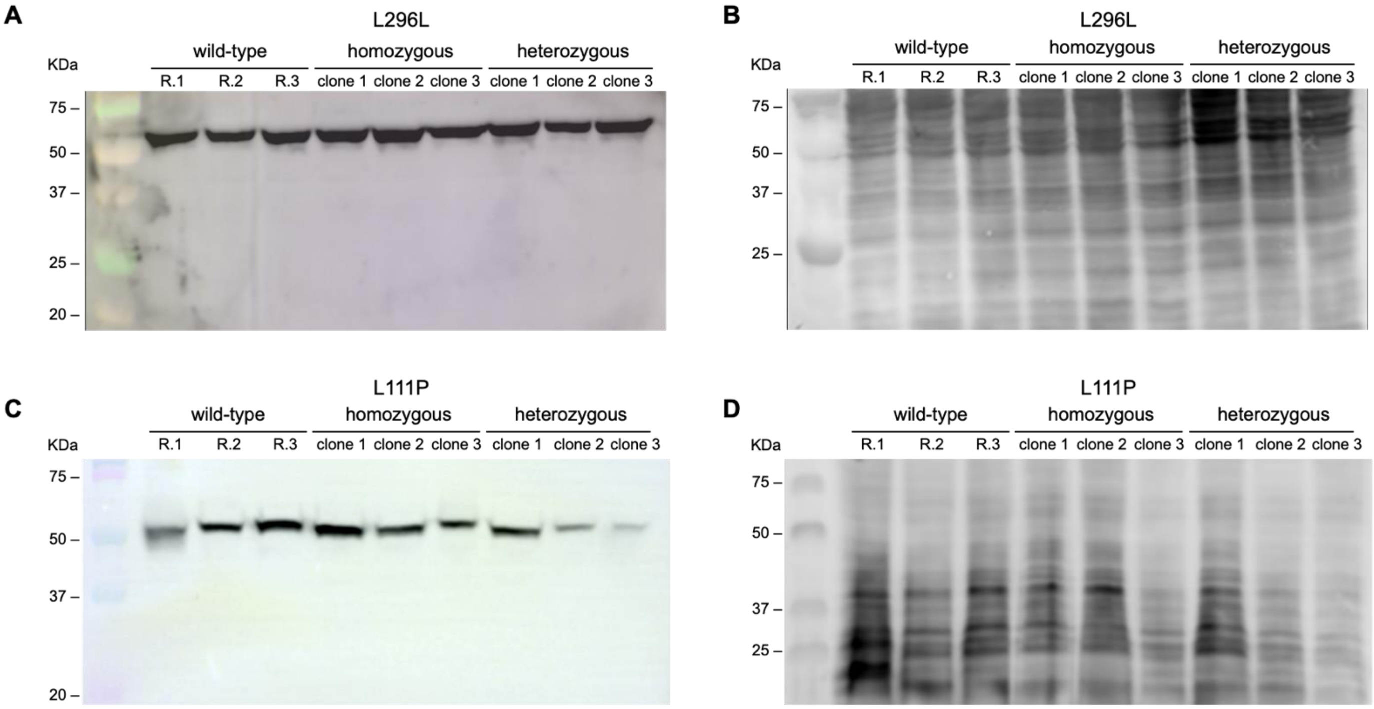

Supplementary Figure 22. Western blotting of MUTYH and total protein expression levels of L296L and L111P isogenic cell lines. L296L (A-B) and L111P (C-D) isogenic cell lines were lysed and stained for MUTYH (A and C) or total (B and D) protein expression levels.

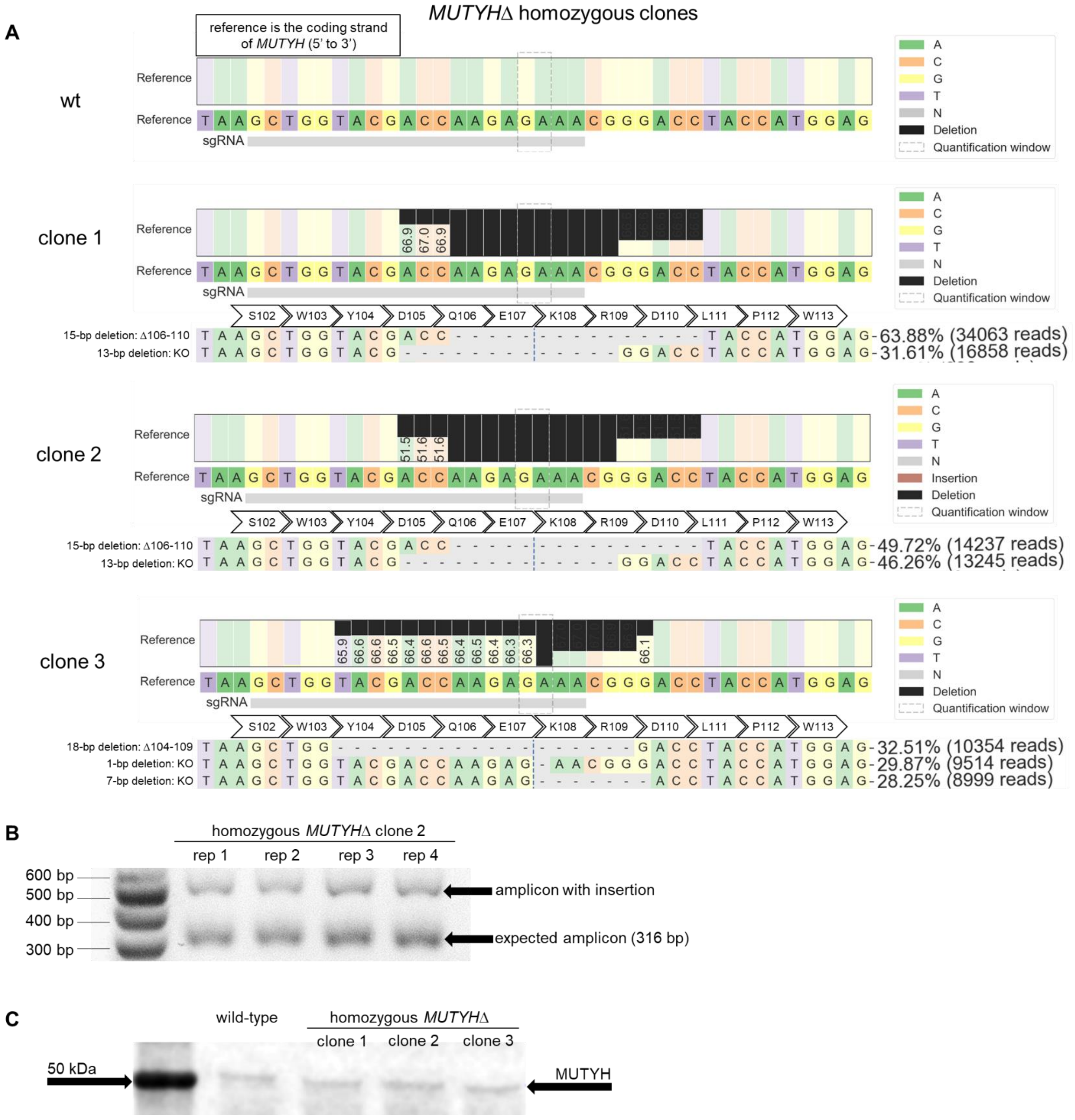

Supplementary Figure 23. NGS and western blot analysis of *MUTYH* deletion (*MUTYH*Δ) homozygous isogenic cell lines. Isogenic cell lines harboring deletion mutations in *MUTYH* near the region encoding amino acids 107-108 were generated as shown in Supplementary Figure 5C. (A) The *MUTYH* locus surrounding codon 107 (amino acid frame shown in boxed arrows) of the clones and wild-type HEK293T cells was sequenced with NGS. Shown are nucleotide percentage tables and allele sequences of the protospacer (indicated with grey line) and 17 base-pairs on the 3' side. The nucleotide percentage table shows the composition of each base at each position in the amplicon, according to the colors shown on the right. The allele plots show the individual deletion sequences and their corresponding frequencies. The gRNA sequence shown is in the 5' to 3' orientation, targeting the coding strand of *MUTYH*; reference shown is in the 5' to 3' orientation. (B) Agarose gel analysis of the PCR product following amplification of the *MUTYH* locus in the *MUTYH*Δ clone 2 (shown are four different replicates). Two different-sized bands were observed, one with the expected size (316 bp), and one that was

~200 bp larger, in an intensity ratio of 2:1. Only the band with the expected size was sequenced with NGS, as the larger band was too long for Illumina sequencing. (C) The cell lines were lysed and stained for MUTYH protein.

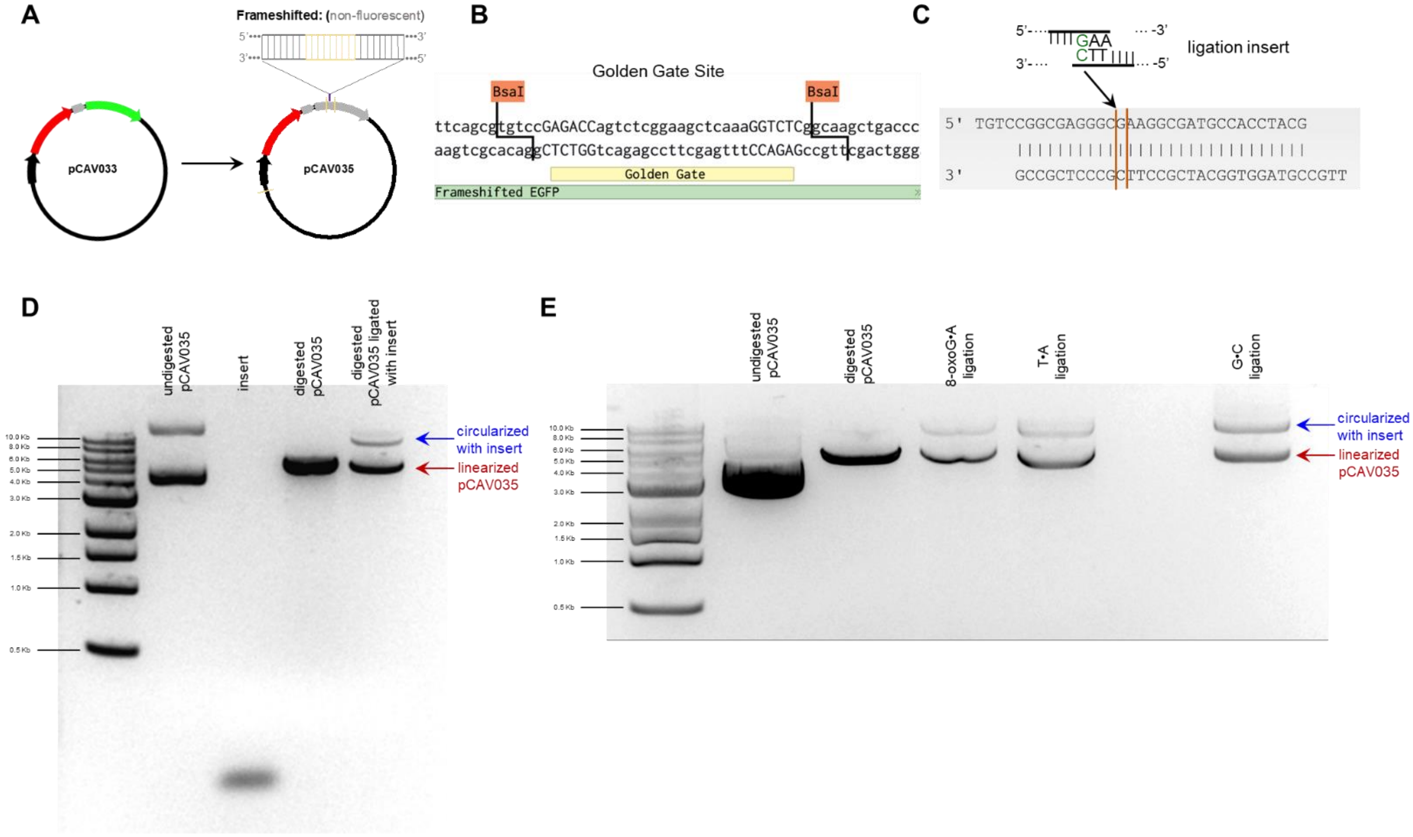

Supplementary Figure 24. Schematic and representative agarose gel images of generation of fluorescent reporter plasmids to measure 8-oxoG•A repair. (A) pCAV033, an mCherry-P2A-EGFP construct in which mCherry and EGFP are transcribed on the same mRNA transcript but translated into separate proteins, was modified into pCAV035 by incorporating a dual BsaI recognition sequence (Golden Gate site, or GG site) that frameshifts EGFP. (B) Sequence of the Golden Gate Site. (C) Digestion of pCAV035with BsaI produces two 4-bp overhangs which can be exploited to ligate custom oligonucleotides into the digested site. Shown is the sequence of the G•C base-pair-containing insert. (D) Agarose gel analysis showing the migration of pCAV035 pre-digestion, post-digestion, and post-ligation. Lane 1: 1kb+ Quickload DNA ladder (NEB), Lane 2: Supercoiled, undigested pCAV035 plasmid (4.9 kbp). Lane 3: Insert (33 bp). Lane 4: pCAV035 plasmid digested with BsaI, resulting in linearized DNA (4.9 kbp). Lane 5: Ligation of the insert into digested pCAV035. (E) Digestion analysis showing migration of pre-digested pCAV035, post-digested pCAV035, and several ligation products. Lane 1: 1kb+ Quickload DNA ladder (NEB), Lane 2: Supercoiled, undigested pCAV035 plasmid (4.9 kbp). Lane 3: pCAV035 plasmid digested with BsaI, resulting in linearized DNA (4.9 kbp). Lane 4: Ligation of 8-oxoG•A-containing insert into digested pCAV035. Lane 5: Ligation of T•A-containing insert into digested pCAV035. Lane 6: Ligation of G•C-containing insert into digested pCAV035.

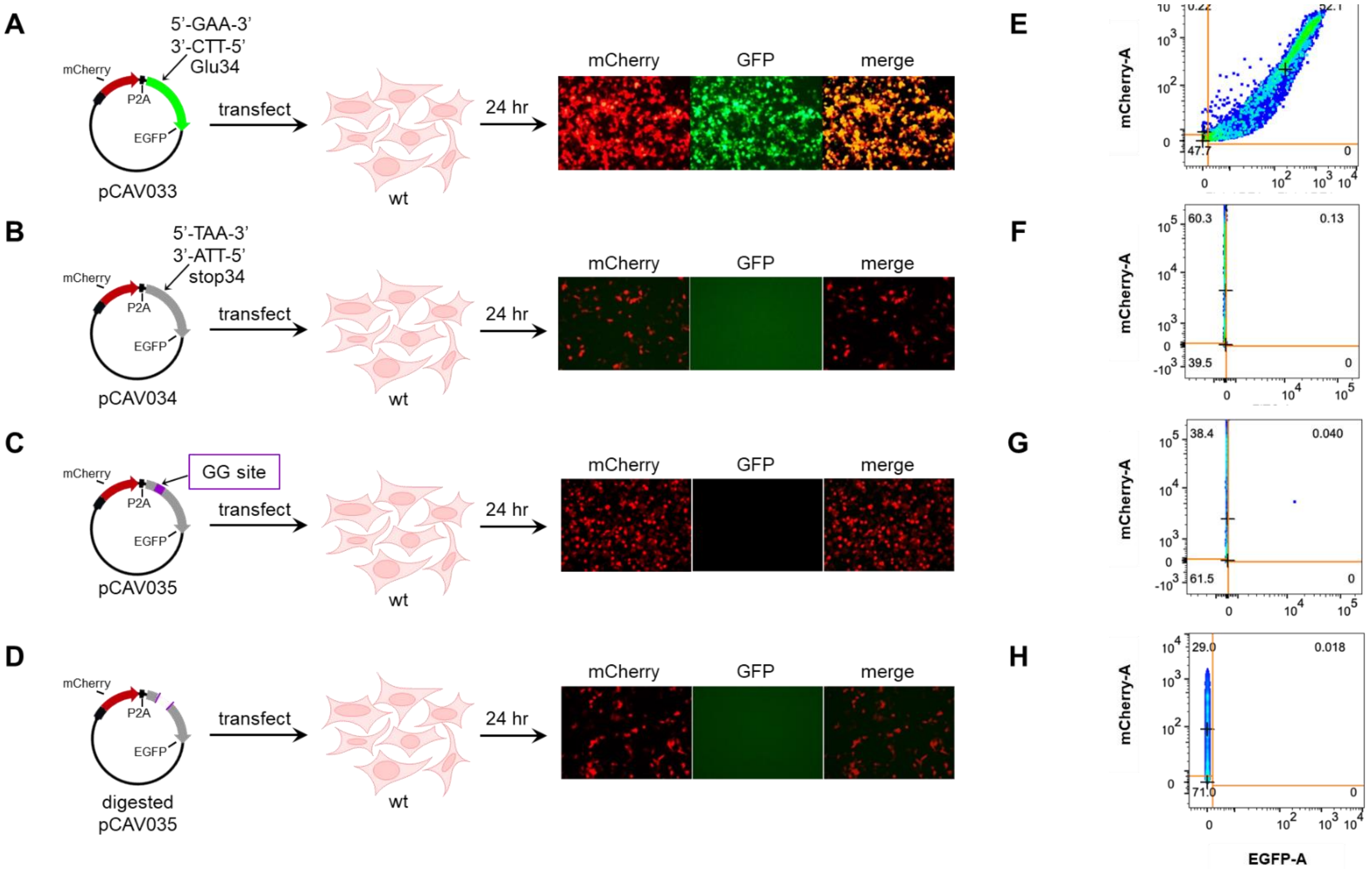

Supplementary Figure 25. Fluorescence microscopy imaging and flow cytometry plots of positive and negative control intact constructs for the 8-oxoG•A repair reporter in wild-type HEK293T cells. (A-D) Fluorescence microscopy images 24 hours after WT HEK293T cells were transfected with pCAV033 (harboring a mCherry-P2A-EGFP transcript, A), pCAV034 (harboring a mCherry-P2A-EGFP with a Stop at codon 34 of EGFP, B), pCAV035 (harboring a mCherry-P2A-frameshifted EGFP with BsaI-HFv2 recognition sequences on the N-terminus of EGFP, C), or pCAV035 digested with BsaI (D). (E-H) Flow cytometry analysis of the samples in A-D are shown. The plots show compensated red fluorescence intensity (*y*-axis) versus compensated EGFP fluorescence intensity (*x*-axis). The numbers in each quadrant represents the percentage of cells within that population. “+”’s in the quadrants indicate the median EGFP fluorescence intensity of EGFP-positive cells.

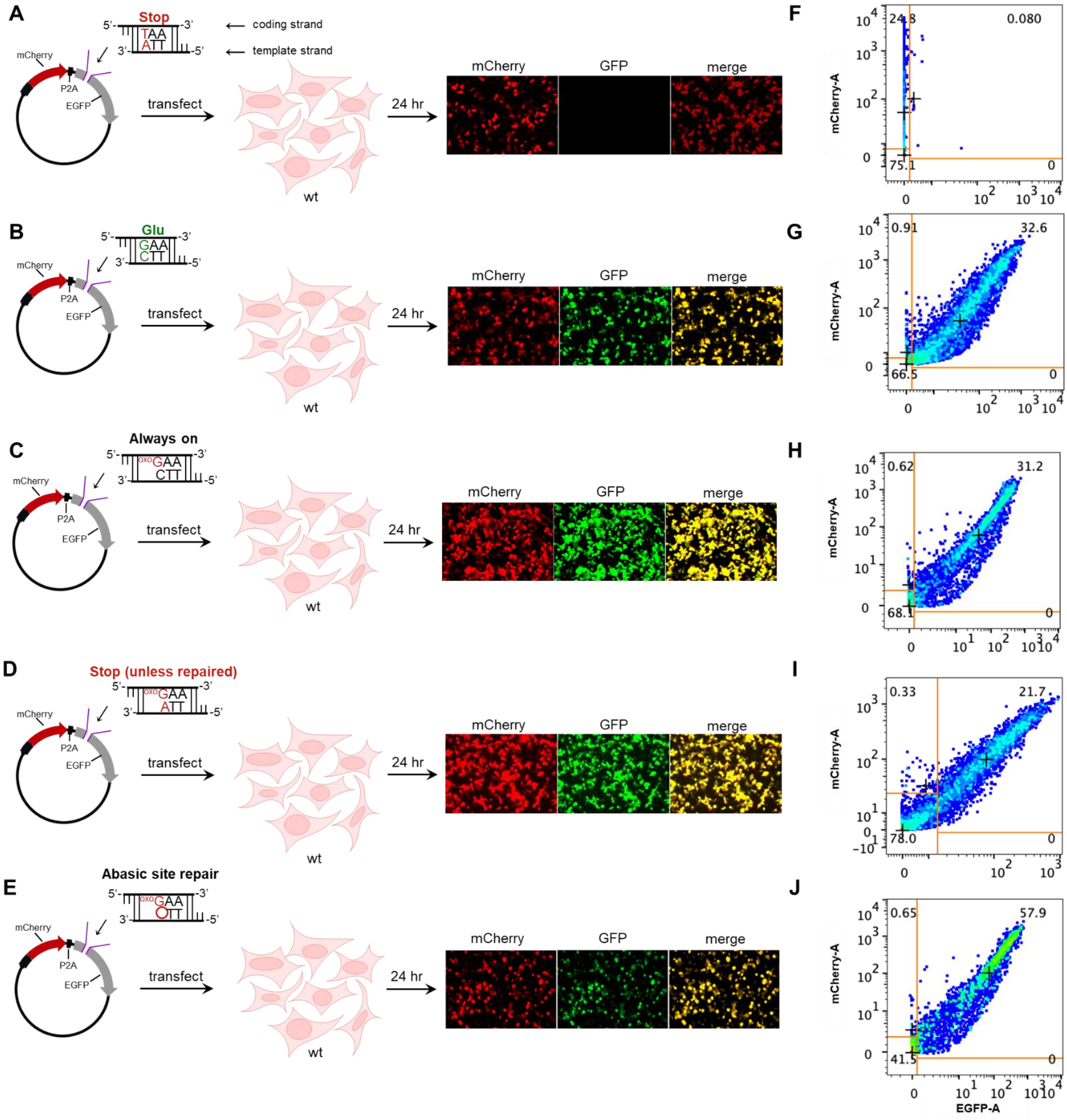

Supplementary Figure 26. Fluorescence microscopy imaging and flow cytometry plots of the 8-oxoG•A and 8-oxoG•[O] repair reporters, as well as positive and negative controls after insert ligation into the pCAV035 backbone in HEK293T cells. (A-E) Fluorescence microscopy images 24 hours after WT HEK293T cells were transfected with digested pCAV035 ligated with inserts containing a T·A (negative control, A), G·C (positive control, B), 8-oxoG•C (positive control, C), 8-oxoG•A (D), or 8-oxoG•[O] (abasic site, E) at codon 34. (F-J) Flow cytometry analysis of the samples in A-E are shown. The plots show compensated red fluorescence intensity (y-axis) versus compensated EGFP fluorescence intensity (*x*-axis). The numbers in each quadrant represents the percentage of cells within that population. "+"'s in the quadrants indicate the median EGFP fluorescence intensity of EGFP-positive cells. See Figure 3B for quantification of flow cytometry plots.

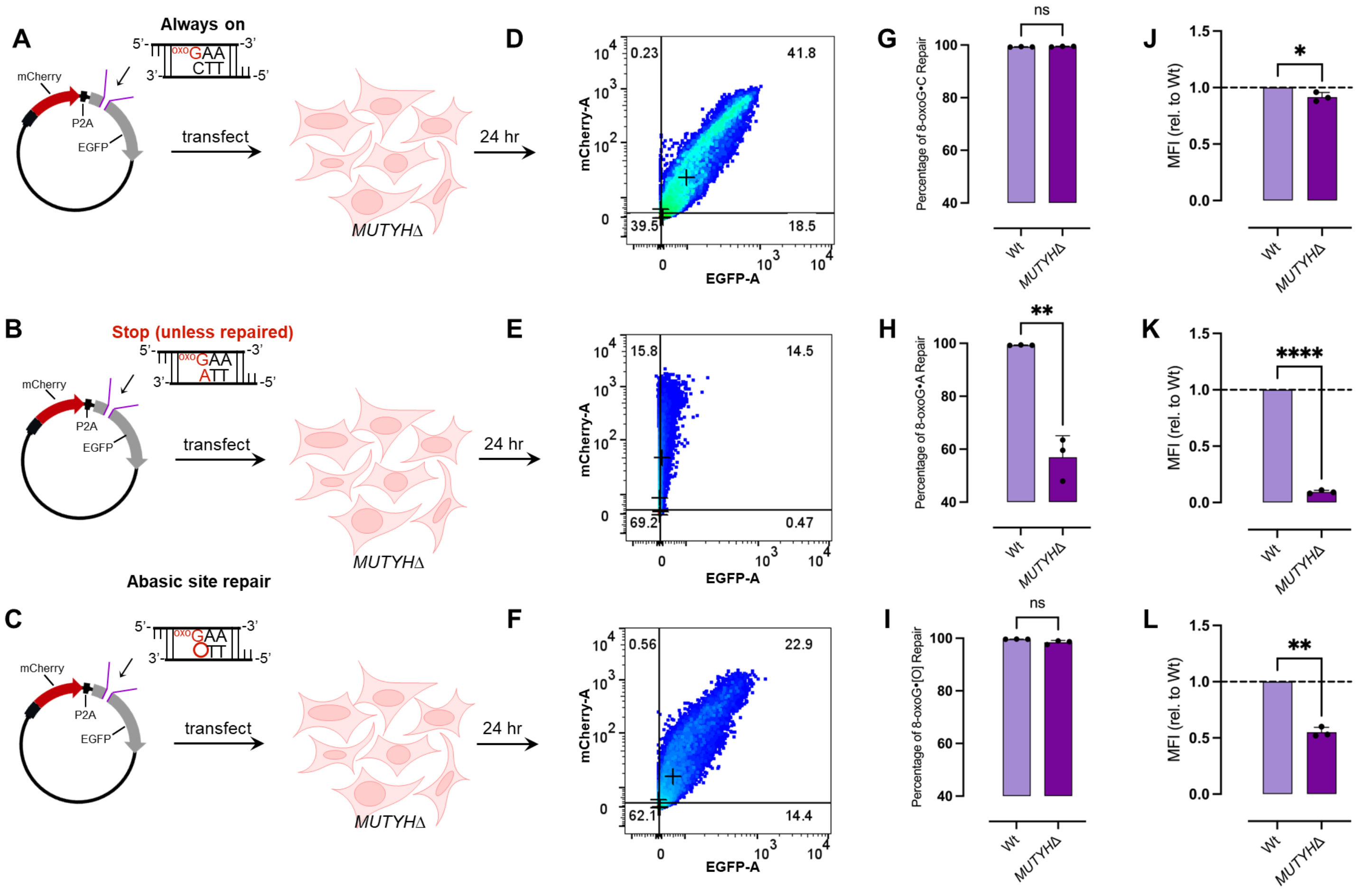

Supplementary Figure 27. Flow cytometry plots of the 8-oxoG•C, 8-oxoG•A, and 8-oxoG•[O] reporters in *MUTYH*Δ cells. (A-C) Schematic diagram of the fluorescent reporters for 8-oxoG•C (A), 8-oxoG•A (B), and 8-oxoG•[O] (C) repair. (D-F) Representative flow cytometry analysis of the samples in A-C are shown. The plots show compensated red fluorescence intensity (y-axis) versus compensated EGFP fluorescence intensity (*x*-axis). The numbers in each quadrant represents the percentage of cells within that population. "+"'s in the quadrants indicate the median EGFP fluorescence intensity of EGFP-positive cells. (G-I) Average repair of reporters for 8-oxoG•C (G), 8-oxoG•A (H), and 8-oxoG•[O] (I) repair in *MUTYH*Δ cell lines, calculated by dividing the percent of EGFP+ cells by the percent of transfected, or mCherry+, cells. (J-L) Average median fluorescence intensities (MFIs) of EGFP+ cells relative to that of the unedited HEK293T cells for 8-oxoG•C (J), 8-oxoG•A (K), and 8-oxoG•[O] (L) repair in *MUTYH*Δ cell lines. Values and error bars represent the mean and standard deviation of three biological replicates, respectively, with each replicate marked individually. Data were analyzed with unpaired, one-tailed, parametric t-tests (in which the heterozygous or homozygous lines were compared to their wild-type counterparts), and p values are marked as follows: ns=p≥0.05 not significant, and *p ≤ 0.05 , **p ≤ 0.01, and *** p ≤ 0.001 are significant.

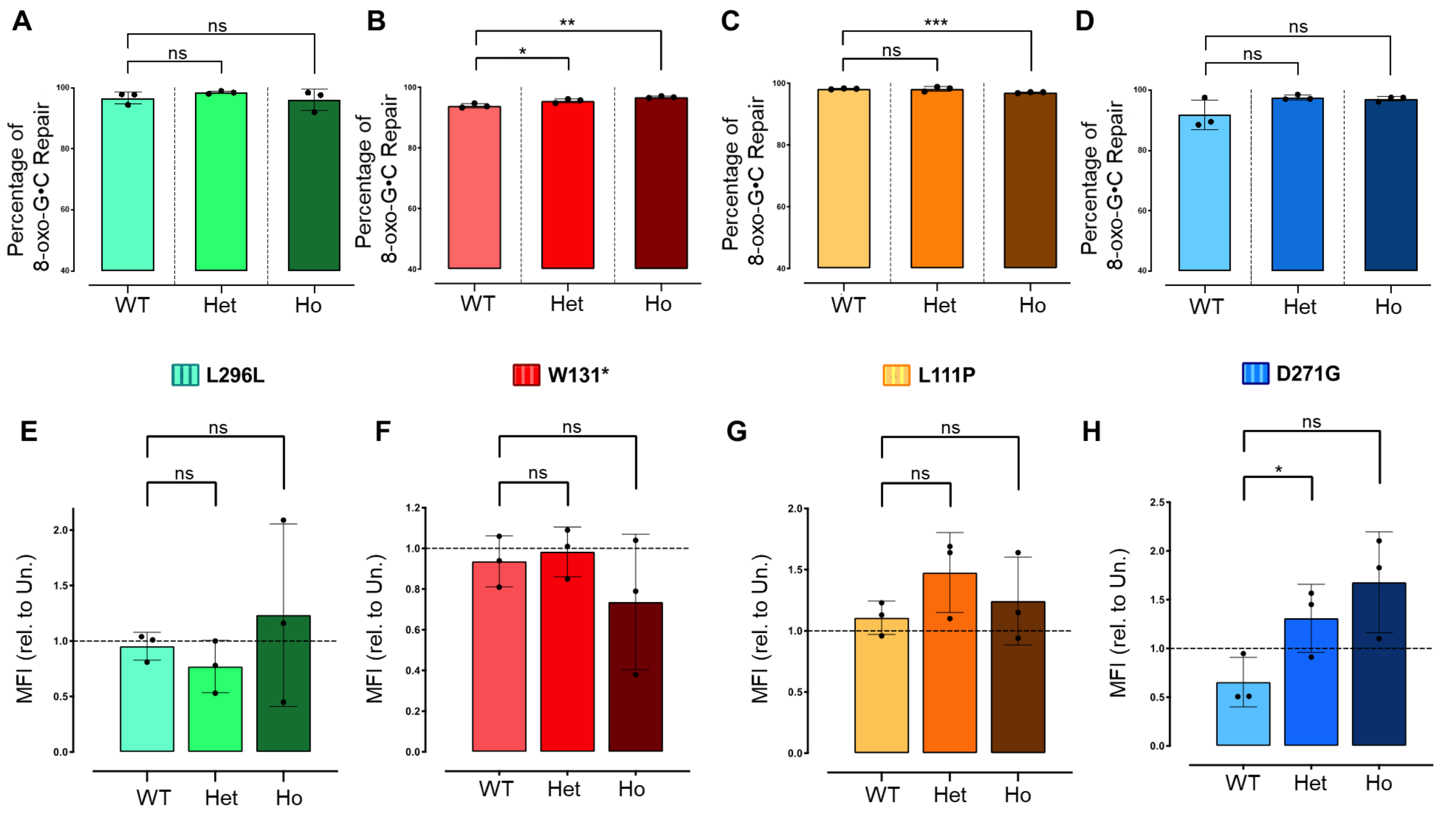

Supplementary Figure 28. Quantification of 8-oxoG•C repair in all isogenic cell lines. (A-D) The three isogenic cell line clones for each *MUTYH* variant genotype L296L (A), W131* (B), L111P (C), and D271G (D) were transfected with the 8-oxoG•C repair reporter plasmid as shown in Supplementary Figure 26C. Repair was quantified by flow cytometry after 24 hours by calculating the percent of EGFP+ cells divided by the transfected, or mCherry+, cells. (E-H) Average median fluorescence intensities (MFIs) of EGFP+ cells relative to that of the unedited HEK293T cells for 8-oxoG•C repair for all *MUTYH* variant genotypes. Values and error bars represent the mean and standard deviation of the three biological replicates, respectively, with each replicate marked individually. Data were analyzed with unpaired, one-tailed, parametric t-tests (in which the heterozygous or homozygous lines were compared to their wild-type counterparts), and p values are marked as follows: ns=p≥0.05 not significant, and *p ≤ 0.05 , **p ≤ 0.01, and *** p ≤ 0.001 are significant.

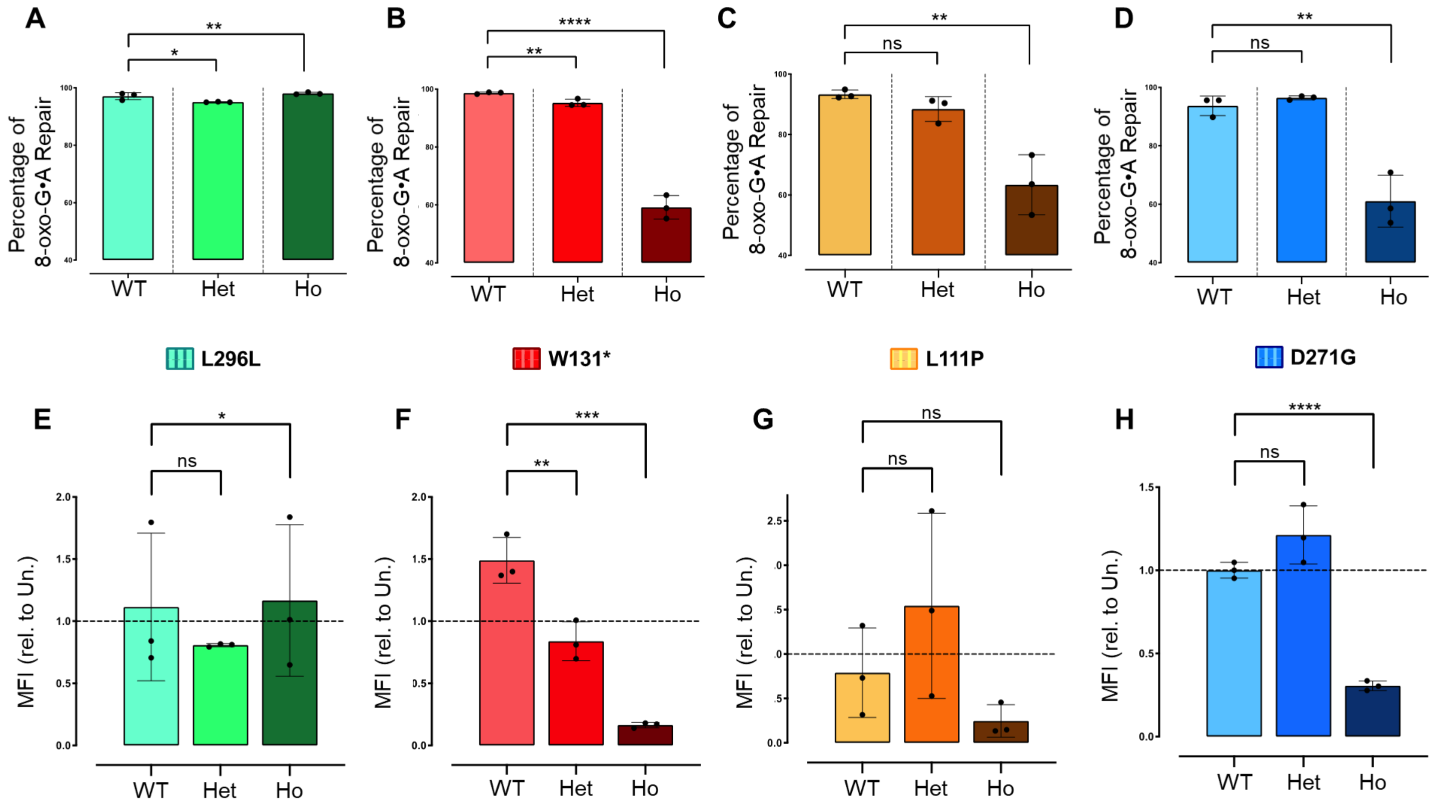

Supplementary Figure 29. Quantification of 8-oxoG•A repair in all isogenic cell lines. (A-D) The three isogenic cell line clones for each *MUTYH* variant genotype L296L (A), W131* (B), L111P (C), and D271G (D) were transfected with the 8-oxoG•A repair reporter plasmid as shown in Supplementary Figure 26D. Repair was quantified by flow cytometry after 24 hours by calculating the percent of EGFP+ cells divided by the transfected, or mCherry+, cells. (E-H) Average median fluorescence intensities (MFIs) of EGFP+ cells relative to that of the unedited HEK293T cells for 8-oxoG•A repair for all *MUTYH* variant genotypes. Values and error bars represent the mean and standard deviation of the three biological replicates, respectively, with each replicate marked individually. Data were analyzed with unpaired, one-tailed, parametric t-tests (in which the heterozygous or homozygous lines were compared to their wild-type counterparts), and p values are marked as follows: ns=p≥0.05 not significant, and *p ≤ 0.05 , **p ≤ 0.01, and *** p ≤ 0.001 are significant.

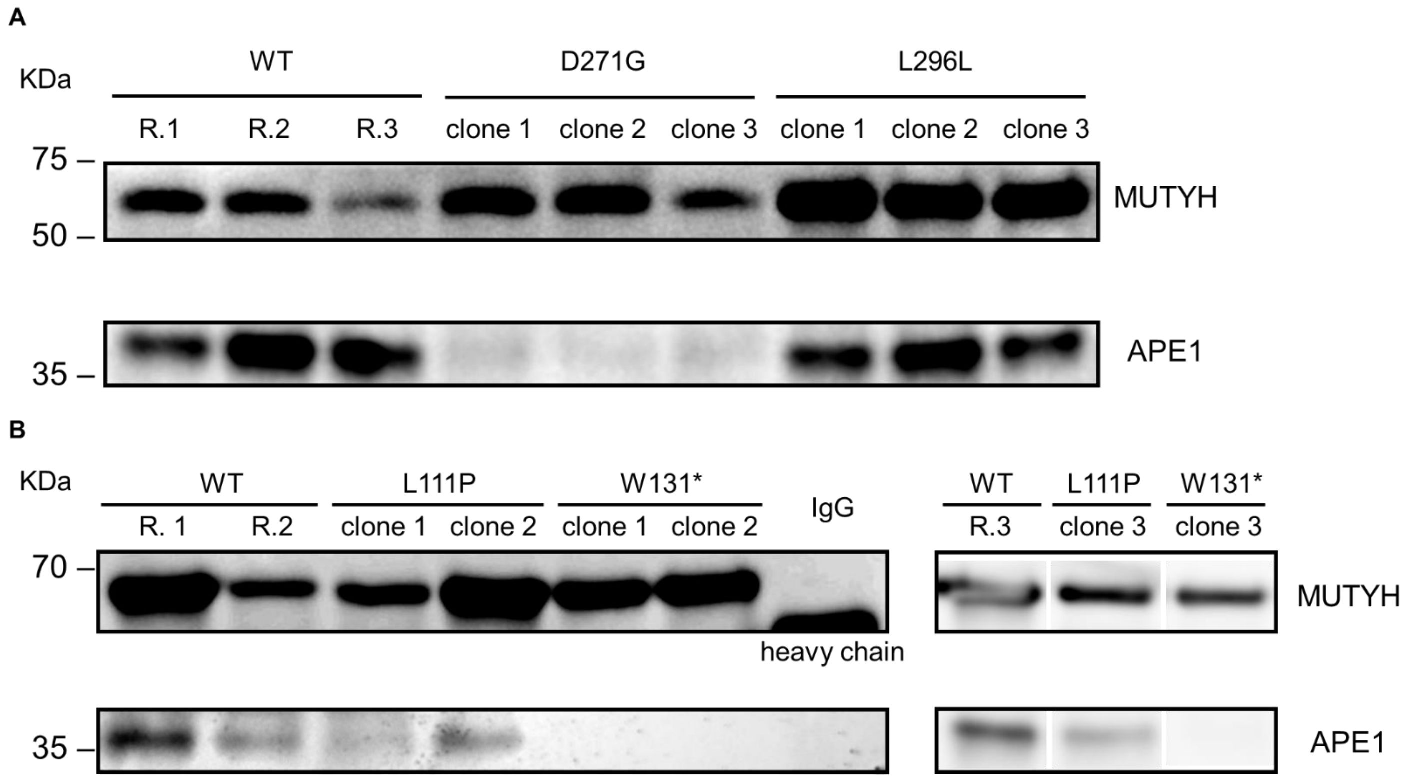

Supplementary Figure 30. Co-immunoprecipitation followed by western blot experiments to probe MUTYH-APE1 interactions in homozygous mutant MUTYH cell lines. The isogenic cell line lysates were prepared as described in the methods section. Lysates were subjected to co-immunoprecipitation (Co-IP) using MUTYH antibody. Eluted complexes were analyzed by western blot (WB) analysis using an antibody against MUTYH or APE1. (A) Untreated HEK293T cell lysates show anti-MUTYH antibody pulls down MUTYH and the interaction with APE1 is detected with anti-APE1 (*lanes 1-3*). Interaction of MUTYH with APE1 is affected by the D271G mutation in *MUTYH* (*lanes 4-6*) but not affected by the L296L *MUTYH* mutation (*lanes 7-9*). (B) Untreated HEK293T cell lysates show anti-MUTYH antibody pulls down MUTYH and the interaction with APE1 is detected with anti-APE1 (*lanes 1-2, 8*). Interaction of MUTYH with APE1 is not affected by the L111P *MUTYH* mutation (*lanes 3-4, 9*) but is affected by the W131* mutation in *MUTYH* (*lanes 5-6, 10*). As a control, lysates were also subjected to an anti-Mouse IgG antibody and the eluted complex were also probed against MUTYH and APE1 (*lane 7*). The three clones for each genotype represent n=3 biological replicates.

Supplementary Table 1. List of primers used for PCR amplification to produce gRNA plasmids

Use	Number	Primer	Sequence
gRNA Cloning	0	Universal FWD	GTTTTAGAGCTAGAAATAGCAAGTTAAAATAAGGC
	1	W12* gRNA REV	CCTGAGTCGTCTGTGGGTACGGTGTTTCGTCCTTTCCACAAG
	2	P18L gRNA REV	ACGGCTGCTCGTGGCTTCCTCGGTGTTTCGTCCTTTCCACAAG
	3	L111P gRNA REV	AGAGAAACGGGACCTACCATCGGTGTTTCGTCCTTTCCACAAG
	4	Y128H gRNA REV	GGACAGGCGGGCATATGCTGCGGTGTTTCGTCCTTTCCACAAG
	5	W131* gRNA REV	TTTCCCCCAGTGTGGGTCTCGGTGTTTCGTCCTTTCCACAAG
	6	Y179C gRNA REV	GGCCACGAGAATAGTAGCCCGGTGTTTCGTCCTTTCCACAAG
	7	R182C gRNA REV	GCAGCCGCCGGCCACGAGAACGGTGTTTCGTCCTTTCCACAAG
	8	G189E gRNA REV	GCGGCTGCAGGAGGGAGCTCGGTGTTTCGTCCTTTCCACAAG
	9	I223V gRNA REV	AAGGCGATAGAGGCAATGGCGGTGTTTCGTCCTTTCCACAAG
	10	R241W gRNA REV	GACACGGCACAGCACCCGTGCGGTGTTTCGTCCTTTCCACAAG
	11	R245C gRNA REV	CCAATGGCTCGGACACGGCACGGTGTTTCGTCCTTTCCACAAG
	12	V246I gRNA REV	CGGGTGCTGTGCCGTGTCCGCGGTGTTTCGTCCTTTCCACAAG
	13	Q260* gRNA REV	TCCTACCAGAGCTGCTGGGACGGTGTTTCGTCCTTTCCACAAG
	14	D271G gRNA REV	CCTGGCCGGGCTGGGTCCACGGTGTTTCGTCCTTTCCACAAG
	15	P295L gRNA REV	CTGGCTGCACAGTGGGCGCTCGGTGTTTCGTCCTTTCCACAAG
	16	L296L gRNA REV	GGGCACTGGCTGCACAGTGGCGGTGTTTCGTCCTTTCCACAAG
	17	E303E gRNA REV	GTGCCCTGTGGAGAGCCTGTCGGTGTTTCGTCCTTTCCACAAG
	18	S304N gRNA REV	GTGCCCTGTGGAGAGCCTGTCGGTGTTTCGTCCTTTCCACAAG

Underlined sequences anneal to the gRNA plasmid backbone.

Supplementary Table 2. List of primers used for Sanger sequencing and Next-generation sequencing (NGS) for *MUTYH* genomic DNA

Use	Number	Primer	Sequence
Sanger Sequencing	19	gRNA plasmid sequencing (U6)	TACGTGACGTAGAAAGTAAT
	20	Sanger FWD: MUTYH codons 1-12	TCTCCCAGAGCGCAGAGGCTTT
	21	Sanger REV: MUTYH codons 1-12	CTCCTAGTCTAACTCCTGGGCGTGC
	22	Sanger FWD: MUTYH codons 16-55	GCAGAGAAACCGCCTACCCCCA
	23	Sanger REV: MUTYH codons 16-55	CTACAGACGCTCACCACCACGC
	24	Sanger FWD: MUTYH codons 56-168	AGCCAGTAGTACCACCCTGAGA
	25	Sanger REV: MUTYH codons 56-168	GCCCAGAGTTGATTCACCTCCT
	26	Sanger FWD: MUTYH codons 176-332	AGGAGGTGAATCAACTCTGGGC
	27	Sanger REV: MUTYH codons 176-332	CCGAACCCTACTCAAGCCAAGA
NGS	28	NGS rd1 adapter FWD	ACACTCTTTCCCTACACGACGCTCTTCCGATCTNNNN
	29	NGS rd1 adapter REV	TGGAGTTCAGACGTGTGCTCTTCCGATCT
	30	NGS rd1 *MUTYH* D271G and L296L FWD	ACACTCTTTCCCTACACGACGCTCTTCCGATCTNNNNAGCAGCTCTGGTAGGATGTTGG
	31	NGS rd1 *MUTYH* D271G and L296L REV	TGGAGTTCAGACGTGTGCTCTTCCGATCTCCCAGTAGGCTTACTCTCTGGC
	32	NGS rd1 *MUTYH* W131* FWD	ACACTCTTTCCCTACACGACGCTCTTCCGATCTNNNNCAGGCGGGCATATGCTGGTCAG
	33	NGS rd1 *MUTYH* W131* REV	TGGAGTTCAGACGTGTGCTCTTCCGATCTCCCCTGGAGTCACCTGCATCCA

Supplementary Table 3. List of oligos used to ligate into the fluorescent reporter backbone (pCAV035)

Use	Number	Oligo	Sequence
Anneal with Complimentary Oligo then Ligate into BsaI-Digested pCAV035 backbone	34 (compatible with 37)	FWD oligo to introduce T•A ligation at codon 34 of EGFP (T bp on the first bp of codon 34)	tgtccggcgagggc**t**aaggcgatgccacctacg
	35 (compatible with 38)	FWD oligo to introduce G•C ligation at codon 34 of EGFP (G bp on the first bp of codon 34)	tgtccggcgagggc**g**aaggcgatgccacctacg
	36 (compatible with 37, 38, and 39)	FWD oligo to introduce 8-oxoG•A, or 8-oxoG•C, or 8-oxoG•[O] ligation at codon 34 of EGFP (8-oxoG bp on the first bp of codon 34)	tgtccggcgagggc/**8oxodG**/aaggcgatgccacctacg
	37	REV oligo to introduce T•A or 8-oxoG•A ligation at codon 34 of EGFP (A bp on the complimentary strand opposite of the first bp of codon 34)	ttgccgtaggtggcatcgcctt**a**gccctcgccg
	38	REV oligo to introduce G•C or or 8-oxoG•C ligation at codon 34 of EGFP (C bp on the complimentary strand opposite of the first bp of codon 34)	ttgccgtaggtggcatcgcctt**c**gccctcgccg
	39	REV oligo to introduce a 8-oxoG•[O] ligation at codon 34 of EGFP (abasic site on the complimentary strand opposite of the first bp of codon 34, after digestion with UDG)	ttgccgtaggtggcatcgcctt/**ideoxyU**/gccctcgccg

Supplementary Table 4. Off-target loci for the *MUTYH* variants featured in this study.

Targeted off-target amplicon sequencing							
MUTYH variant	protospacer	Site No.	Off-target sequence	PAM	Score	Gene	Locus
**L111P**	ATGGTAGGTCCCGTTTCTCT	1	ATGGTAGCTACCGTTTCTCC	TG	1.3		chr12:-64177925
		2	ATGGGAGTTCCCCTTTCTCT	AG	1.1	RP11-1133J7.1 (ENSG00000255259)	chr11:+129296316
		3	ACGGTTGGCCCCGTTTCTCT	GG	1	KATNB1	chr16:-57743572
		4	CGGGCAGGTCCCGTTTCTCC	AG	0.9	KRTAP10-3 (ENSG00000212935)	chr21:-44558033
**W131***	GAGACCCACACTGGGGGAAA	1	CAACCCCACACTGGGGGAAA	TG	2.4		chr4:-182825360
		2	GACACCTCCACTGGGGGAAA	GG	1.7		chr14:+49683032
		3	CAGACCAAAACTGGGGGAAA	AG	1.2	RP11-727A23.5 (ENSG00000247137)	chr11:+83192800
		4	CAGTGCCACACCGGGGGAAA	GG	0.8	CTD-2501E16.1 (ENSG00000259513)	chr15:+60558142
**D271G**	GTGGACCCAGCCCGGCCAGG	1	GGGGATCCAGCCCGGCCAGG	AG	3.6	PALM (ENSG00000099864)	chr19:+746531
		2	GAGGACCCAGCCAGGCCAGG	AG	3.6		chr22:-20194545
		3	GGGGACCCAGCCGGGCCAGG	CG	3.6		chr1:+3554377
		4	GCGGGCCCGGCCCGGCCAGG	TG	1.7	MYADML2 (ENSG00000185105)	chr17:-81941279
**L296L**	CCACTGTGCAGCCAGTGCCC	1	CCACTGTGCTGCCAGTGCCC	CG	92.1		chr1:+3018439
		2	CCTCTCTGCAGCCAGTGCCC	AG	3.4		chr1:-47888455
		3	CTGCTGTACTGCCAGTGCCC	GG	1.4	ARIH1 (ENSG00000166233)	chr15:-72590119
		4	CTGATGTGCTGCCAGTGCCC	TG	1.4	FNTB (ENSG00000257365)	chr14:+65053317

Red bases indicate mismatches from the protospacer. The "score" value is the predicted off-target score for each site (evaluated using the Hsu et al. off-target prediction algorithm in combination with the Doench et al. on-target efficiency calculator algorithm), which ranges from 0-100 where 100 is a perfect match.

Supplementary Table 5. List of primers used for NGS of off-target loci

MUTYH Variant	Off-Target Site #	FWD Primer	REV Primer
L111P	1	ACACTCTTTCCCTACACGACGCTCTTCCGATCTNNNNTGTGCATGGGACAACCTTCACCT	TGGAGTTCAGACGTGTGCTCTTCCGATCTTCTTGGTGCTAGCTGTGCATGT
	2	ACACTCTTTCCCTACACGACGCTCTTCCGATCTNNNNCTCCTGATGCCTGGTGGACCCA	TGGAGTTCAGACGTGTGCTCTTCCGATCTCGTTATGCAGGGGTGGCTTCCC
	3	ACACTCTTTCCCTACACGACGCTCTTCCGATCTNNNNGCAAGGGTGCCAGGGGTCAGTA	TGGAGTTCAGACGTGTGCTCTTCCGATCTGAGGCTGGTGTGTGTGAGCCAG
	4	ACACTCTTTCCCTACACGACGCTCTTCCGATCTNNNNACTCAGACAGGGCTCAGGGCTG	TGGAGTTCAGACGTGTGCTCTTCCGATCTGTCCACCTGCTGTGTGCCCATC
W131*	1	ACACTCTTTCCCTACACGACGCTCTTCCGATCTNNNNTGGAATGAGCCACGCACTGCTG	TGGAGTTCAGACGTGTGCTCTTCCGATCTAGGACTCTGCAGCACCCAGTGA
	2	ACACTCTTTCCCTACACGACGCTCTTCCGATCTNNNN GCCACTAGGAGAGACAAAGTGACCA	TGGAGTTCAGACGTGTGCTCTTCCGATCTTCGGCCTCCCAAAGTGCTAGGA
	3	ACACTCTTTCCCTACACGACGCTCTTCCGATCTNNNNACCTCAAGAGAGTAGCGGTAGGA	TGGAGTTCAGACGTGTGCTCTTCCGATCTGGATTCCCCTCTCGGCCCAGTT
	4	ACACTCTTTCCCTACACGACGCTCTTCCGATCTNNNNTGGAGAGCAGGATGTTTGGCCT	TGGAGTTCAGACGTGTGCTCTTCCGATCTAGGCCTCCTGACCTGTCCTCCA
D271G	1	ACACTCTTTCCCTACACGACGCTCTTCCGATCTNNNNAGCTCCTCCGAGGTGGACGAAC	TGGAGTTCAGACGTGTGCTCTTCCGATCTAGCACCTTCTTGGTCTCGGCCT
	2	ACACTCTTTCCCTACACGACGCTCTTCCGATCTNNNNAACCCATCCCCCTGGACATGGG	TGGAGTTCAGACGTGTGCTCTTCCGATCTAAAACCCTTGCCTGGCGTGGAC
	3	ACACTCTTTCCCTACACGACGCTCTTCCGATCTNNNNGTTCCAACACTGGGCTGGGAGC	TGGAGTTCAGACGTGTGCTCTTCCGATCTCTGCCTCCTGTAGCAGCTGGGA
	4	ACACTCTTTCCCTACACGACGCTCTTCCGATCTNNNNGGACGATCTTGAGGAGCCCCGA	TGGAGTTCAGACGTGTGCTCTTCCGATCTGTGAGTTCACACGGCTCCACGG
L296L	1	ACACTCTTTCCCTACACGACGCTCTTCCGATCTNNNNGCATCTCGGCCACGACTCTCCT	TGGAGTTCAGACGTGTGCTCTTCCGATCTCCTGCCAGTCCCCGTTTTGGAG
	2	ACACTCTTTCCCTACACGACGCTCTTCCGATCTNNNNGCAGGGGGCTTTCTGTTTGGAGG	TGGAGTTCAGACGTGTGCTCTTCCGATCTGGGACAGGAGGTAGCGATGGCA
	3	ACACTCTTTCCCTACACGACGCTCTTCCGATCTNNNNTGCCACTGCTCCTAGCTCTTCCA	TGGAGTTCAGACGTGTGCTCTTCCGATCTGCCTTGGGCAATCCACCGAGTC
	4	ACACTCTTTCCCTACACGACGCTCTTCCGATCTNNNNGGATCTGCCGGGCCCTTACTGA	TGGAGTTCAGACGTGTGCTCTTCCGATCTTGCTCTCTCCTCCCCTCTCCCT

Supplementary Sequences

Full-length MUTYH (numbering system used in this work):
Mitochondrial targeting sequence
Alternative splicing extra sequences

MTPLVSRLSRLWAIMRKPRAAVGSGHRKQAASQEGRQKHAKNNSQAKPSACDACAGMIAECPGAPAGLARQ
PEEVVLQASVSSYHLFRDVAEVTAFRGSLLSWYDQEKRDLPWRRRAEDEMDLDRRAYAVWVSEVMLQQTQV
ATVINYYTGWMQKWPTLQDLASASLEEVNQLWAGLGYYSRGRRLQEGARKVVEELGGHMPRTAETLQQLLP
GVGRYTAGAIASIAFGQATGVVDGNVARVLCRVRAIGADPSSTLVSQQLWGLAQQLVDPARPGDFNQAAMEL
GATVCTPQRPLCSQCPVESLCRARQRVEQEQLLASGSLSGSPDVEECAPNTGQCHLCLPPSEPWDQTLGVV
NFPRKASRKPPREESSATCVLEQPGALGAQILLVQRPNSGLLAGLWEFPSVTWEPSEQLQRKALLQELQRWA
GPLPATHLRHLGEVVHTFSHIKLTYQVYGLALEGQTPVTTVPPGARWLTQEEFHTAAVSTAMKKVFRVYQGQQ
PGTCMGSKRSQVSSPCSRKKPRMGQQVLDNFFRSHISTDAHSLNSAAQ

Mammalian reporters used for measuring DNA repair:

mCherry

P2A

EGFP

NLS

Golden Gate Site

E = Glutamic Acid at codon 34 (codon:GAA)

pCAV033 (mCherry-P2A-EGFP)

MVSKGEEDNMAIIKEFMRFKVHMEGSVNGHEFEIEGEGEGRPYEGTQTAKLKVTKGGPLPFAWDILSPQFMY
GSKAYVKHPADIPDYLKLSFPEGFKWERVMNFEDGGVVTVTQDSSLQDGEFIYKVKLRGTNFPSDGPVMQKK
TMGWEASSERMYPEDGALKGEIKQRLKLKDGGHYDAEVKTTYKAKKPVQLPGAYNVNIKLDITSHNEDYTIVE
QYERAEGRHSTGGMDELYKSGATNFSLLKQAGDVEENPGPMVSKGEELFTGVVPILVELDGDVNGHKFSVSG
EGEGDATYGKLTLKFICTTGKLPVPWPTLVTTLTYGVQCFSRYPDHMKQHDFFKSAMPEGYVQERTIFFKDDG
NYKTRAEVKFEGDTLVNRIELKGIDFKEDGNILGHKLEYNYNSHNVYIMADKQKNGIKVNFKIRHNIEDGSVQLA
DHYQQNTPIGDGPVLLPDNHYLSTQSALSKDPNEKRDHMVLLEFVTAAGITLGMDELYKSGGSPKKKRKV

pCAV035 (mCherry-P2A-frameshiftedEGFP; GG site; Addgene plasmid #219807)

MVSKGEEDNMAIIKEFMRFKVHMEGSVNGHEFEIEGEGEGRPYEGTQTAKLKVTKGGPLPFAWDILSPQFMY
GSKAYVKHPADIPDYLKLSFPEGFKWERVMNFEDGGVVTVTQDSSLQDGEFIYKVKLRGTNFPSDGPVMQKK
TMGWEASSERMYPEDGALKGEIKQRLKLKDGGHYDAEVKTTYKAKKPVQLPGAYNVNIKLDITSHNEDYTIVE
QYERAEGRHSTGGMDELYKSGATNFSLLKQAGDVEENPGPMVSKGEELFTGVVPILVELDGDVNGHKFSVSE
TSLGSSKVSAS*P*SSSAPPASCPCPGPPS*PP*PMECSASAATPTT*SSTTSSSPPCPKATSRSAPSSSRTTAT
TRPAPR*SSRATPW*TASS*RASTSRRTATSWGTSWSTTTTATTSISWPTSRRTASR*TSRSATTSRTAACSSP
TTTSRTPPSATAPCCCPTTTT*APSPP*AKTPTRSAITWSCWSS*PPPGSLSAWTSCTSGGSPKKKRKV
